# Effects of transient intravascular volume overload on endothelial glycocalyx thickness in healthy anesthetized cats

**DOI:** 10.3389/fvets.2026.1742967

**Published:** 2026-10-14

**Authors:** Ivayla D. Yozova, Neroli Thomson, Mitsuhiro Irie, Rebecca Owen, John S. Munday

**Affiliations:** 1 School of Veterinary Science, Massey University, Palmerston North, New Zealand; 2 Shikoku Veterinary Medical Center, Miki, Japan

**Keywords:** capillaries, feline, fluid overload, GlycoCheckTM, intravenous fluids, microcirculation, perfused boundary region, sidestream dark field videomicroscopy

## Abstract

**Introduction:**

Previous studies suggest that the endothelial glycocalyx is damaged by volume overload. This study assessed the effects of transient intravascular volume overload on estimated endothelial glycocalyx thickness in healthy cats, using sidestream dark field videomicroscopy and the Glycocheck™ software.

**Methods:**

This was a prospective blinded cross-over single-center experimental study on twenty healthy cats. Cats were randomized to either receive 30 mL/kg/10 min of Hartman’s solution (volume overload group) or no fluids (control) with a washout of 7 days. Sublingual mucosa videomicroscopy images were acquired at T0 (baseline), T1 (immediately after intervention), T2 (3 h after end of intervention) and T3 (24 h after end of intervention) with three measurements performed per timepoint under propofol anesthesia. The perfused boundary region (PBR), an inverse estimate for endothelial glycocalyx thickness, for vessels with diameter of 5–25, 5–9, 10–19 and 20–25 μm, respectively, was then automatically calculated with the Glycocheck™ software. Packed cell volume (PCV) was measured at each timepoint. Data normality was assessed using the Shapiro–Wilk test and visual inspection. Linear mixed-effects regression modelling, with cat as a random effect, analyzed interactions between GlycoCheck™ and other parameters.

**Results:**

PBR 5–25 μm, 5–9 μm and 10–19 μm increased over time, reaching significance at T2 (*p = 0.001*, *p = 0.017* and *p = 0.008*, respectively). A significant group x timepoint interaction was identified for PBR 5–9 with decreased values at T2 (*p = 0.028*) and T3 (*p = 0.010*) in the volume overload group. Decreases in PCV were significantly associated with increases in PBR 5–25 μm (*p = 0.003*) and PBR 10–19 μm (*p = 0.001*).

**Conclusion:**

This study did not demonstrate decreases in estimated endothelial glycocalyx thickness after transient volume overload in healthy cats. The PBR did increase over time, suggesting potential anesthetic effects. Changes in PCV affected GlycoCheck™ parameters and this finding should be further explored. Further studies designed with understanding of feline volume kinetics are required to investigate the effects of IV fluids on the feline endothelial glycocalyx.

## Introduction

Intravascular volume overload (VO) and the resulting fluid overload are harmful, as they contribute to interstitial oedema, impair oxygen delivery to cells, and ultimately promote multi-organ dysfunction and mortality (1). Volume overload and fluid overload are commonly iatrogenic, resulting from overzealous intravenous (IV) fluid administration, particularly in critically ill patients with reduced adaptive capacity (2, 3). Volume overload is counteracted by a group of molecules called natriuretic peptides (NP), secreted primarily by cardiac muscle (4, 5). Their action results in natriuresis, inhibition of the renin-angiotensin-aldosterone system, vasorelaxation and increased vascular permeability (5). Vascular permeability increases by NP-induced damage to the endothelial glycocalyx (EG), thus allowing fluid to shift into the interstitial space (4). While these mechanisms are protective in healthy individuals, they can become maladaptive, especially in critically ill patients, potentially exacerbating pre-existing EG damage (2, 6).

Numerous experimental and clinical studies have investigated the effects of VO on the EG with conflicting results, primarily by measuring shed EG damage biomarkers. While some report NP release and EG damage with VO and aggressive IV fluid administration (7–13), others (typically more recent) fail to demonstrate such effects (14–18). Whether these findings are seen mostly in stable patients or critically ill patients with pre-existing EG damage remains controversial (3, 6). Additionally, most studies have focused on the immediate effects of VO, and information on medium- and long-term consequences is lacking.

Cats may be particularly predisposed to VO due to their relatively smaller blood volume compared to dogs and people (19–21), a high incidence of asymptomatic heart disease (22–24) and lower fluid elimination rates (25, 26). For these reasons, cats offer a compelling model to explore effects of IV fluid-induced VO on the EG.

Two methodologies for investigating EG damage are applicable to the clinical setting. The first consists of measuring EG biomarkers presumably shed after damage. However, result interpretation remains challenging - particularly in the context of IV fluid administration, as elevations are often inconsistent, of low magnitude, and influenced by study design (3, 6, 27). Real-time sidestream dark field videomicroscopy (SDFV) of mucosal blood vessels (6) combined with the proprietary GlycoCheck™ software, offers another clinically applicable measure for estimating EG thickness. The software analyses SDFV-acquired images and calculates the perfused boundary region (PBR) an inverse estimate of EG thickness in vessels ranging from 5 to 25 μm in diameter. Higher PBR values indicate thinner (damaged) EG while lower value indicates a thicker (healthy) layer. Tolerance intervals for GlycoCheck™ parameters have been established in cats (28) and feasibility and associations with illness severity have been investigated in an exploratory study in cats with naturally occurring disease (29). Moreover, our group has previously evaluated the immediate effects of a fluid challenge on the EG in heathy anesthetized cats using the GlycoCheck™, though findings have been inconclusive (30).

The aim of this study was to investigate the effects of a transient IV fluid-induced VO on the EG thickness in healthy anesthetized cats using SDFV and the GlycoCheck™ software (31). We hypothesized that VO induced by rapid administration of 30 mL/kg of buffered balanced isotonic crystalloid solution given over 10 minutes would lead to transient increase in PBR of vessels with diameters 5–25 μm and 5–9, 10–19 and 20–25 μm subgroups, indicating decreased EG thickness, compared to a control group. Additionally, decreases in PCV (due to hemodilution and/or anesthetic effects) may affect RBC lateralization in assessed vessels which would increase the above-mentioned PBR.

## Materials and methods

### Study population

This was a prospective, randomized, blinded, cross-over experimental study, approved by Massey University’s Animal Ethics Committee (MUAEC Protocol 22/81) and adhering to the Animal Research Reporting of *In Vivo* Experiments guidelines (32). Twenty healthy research-purposed cats were enrolled. Cats met inclusion criteria if they were healthy based on history, no weight loss, physical examination findings, complete blood count, serum biochemistry and T4 concentrations (performed during the first week of the study prior to intervention) and the two-minute focused echocardiography exam to screen for occult heart disease (33) performed up to two weeks prior to the start of the study. No animals met predefined exclusion criteria for enrolment. Predefined humane endpoints and stopping criteria included clinically relevant anesthetic complications (e.g., cardiovascular instability, respiratory compromise), procedural limitations (e.g., IV catheter dislodgement, resistance to low-stress handling) or any adverse event deemed to compromise animal welfare. In such cases, animals were recovered as appropriate, and no further data were collected at subsequent timepoints.

### Intervention protocol

The cats were placed in individual cages and food withheld 12 h prior to the beginning of each intervention with *ad libitum* access to water. All cats were sedated with gabapentin (Neurontin™, Viatris Specialty LLC) 100 mg PO 90 min prior and butorphanol (Torbugesic, Zoetis Inc.) 0.3 mg/kg SC 30 min prior to intravenous catheter placement in the left or right cephalic vein. Propofol (Provive 1%, Baxter Healthcare Ltd.) was then administered IV to effect (relaxation of jaw tone). Anesthesia was maintained using a continuous rate infusion of propofol (0.05–0.2 mg/kg/min) for the duration of image acquisitions. The cats were spontaneously breathing and not intubated. Anesthesia was overseen by an experienced veterinary technician and anesthetic variables - heart rate (HR), pulse quality, mucous membrane color, capillary refill time, temperature, respiratory rate, pulse oximetry (SpO_2_, Radical 7, Masimo Corporation, Irvine, CA, USA), and oscillometric blood pressure (PetTrust, BioCare Corporation, Taoyuan, Taiwan) were frequently monitored (every 5 min for clinical parameters, 10 min for blood pressure and continuous for pulse oximetry). The timeline of the intervention is presented in Figure 1.

**Figure 1:**
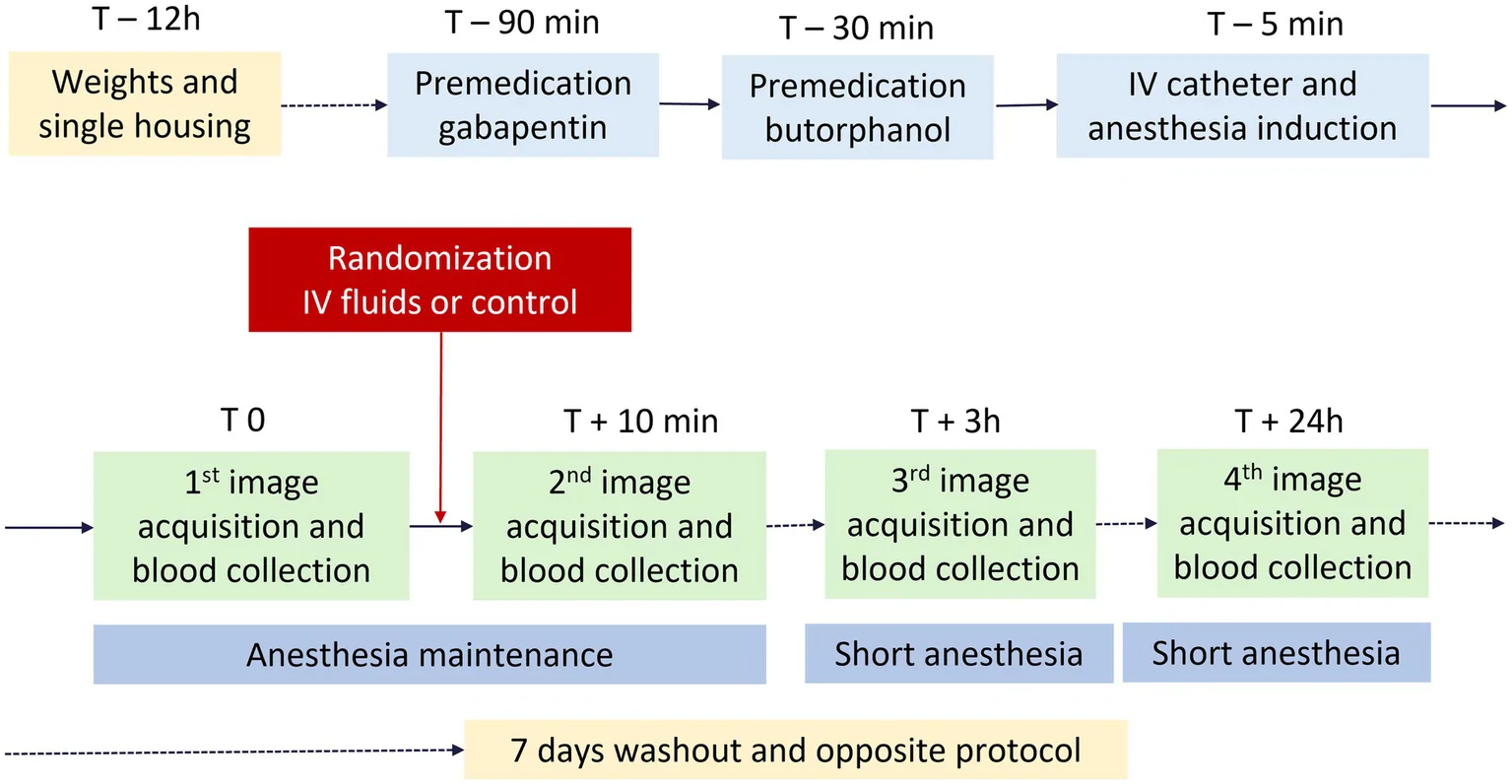
Intervention protocol timeline for a crossover study involving 20 healthy anesthetized cats, randomized to receive either 30 mL/kg of Hartman’s solution over 10 min (volume overload group) or no fluids (control group), with a 7-day washout period between interventions.

Each cat was randomized using an online randomization tool (https://www.randomizer.org/) to either receive 30 mL/kg over 10 min of a buffered balanced isotonic crystalloid solution (Hartman’s solution, Baxter Healthcare Ltd.) via a fluid infusion pump (Niki V4 Volumetric Infusion Pump, Caesarea Medical Electronics Ltd., Israel) at room temperature – volume overload (VO) group; or no fluids - control group. All cats remained under propofol anesthesia for the initial image acquisition (T0), IV fluid administration (or control) and second image acquisition (T1) after which they were allowed to recover and placed in their individual cages to be re-anesthetized briefly with propofol IV for image acquisitions at T2 (3 h) and T3 (24 h) timepoints. After the T2 image acquisition, cats were placed in their individual cages with the IV catheters maintained in place, fed *ad libitum* and food removed 12 h prior to the T3 image acquisition the following day with *at libitum* access to water. At the end of the 24 h period, the IV catheters were removed and the cats returned to their usual group housing. Blood for packed cell volume (PCV) and total solids (TS) measurements was collected under anesthesia via jugular venipuncture at each timepoint. Each cat then underwent a washout period of 7 days after which it received the opposite intervention.

### Image acquisition

All image acquisitions were performed by the same operator (IY), who was blinded to subject allocation by being present only for image acquisitions and leaving the intervention area prior to IV fluids being administered (or not) and returning after completion of the administration. Cats were placed in lateral recumbency, and the base of the tongue exposed such as the sublingual mucosa was accessible for the videomicroscope as previously described (28). A hand-held SDFV camera connected to a laptop with GlycoCheck™ software (MicroVascular Health Solutions, USA) (34) was placed in contact with the sublingual mucosa by the operator. Three video recordings were taken from different sublingual mucosal sites at each of four different time points – T0 (baseline), T1 (end of volume overload or 10 min after baseline for the control group), T2 (3 h after end of intervention) and T3 (24 h after end of intervention).

### GlycoCheck™ analysis

The method for measuring and calculating GlycoCheck™ parameters has been previously described (28). Briefly, the SDFV camera emits green light at 540 nm wavelength which is absorbed by hemoglobin in circulating red blood cells (RBC). Therefore, vessels appear dark on a bright background.

Video recordings are displayed in real-time to allow the operator to assess image quality, including focus, pressure and motion artifacts (35). The software additionally provides linear quality indicator bars for motion, intensity and focus with green segments denoting acceptable ranges and records images only when predefined quality criteria are met. Automatic adjustments for motion, focus and intensity are applied to optimize image acquisition.

Each measurement is performed from approximately 10 video recordings containing 40 frames each (31). The software automatically identifies vessels that are less than 30 μm in diameter by analysing the contrast between the RBC and the background. The software then subdivides vessels into major (10 μm) and subsequently minor (0.5 μm) vascular segments in length and further determines the percentage of minor segments within a major segment with sufficient contrast for analysis (Figure 2). A total of approximately 3,000 major vascular segments including vessels ranging from ~ 3–30 μm diameter are recorded during each measurement. The counts for major vascular segments of each analysed diameter are additionally recorded. Major segments with > 60% of minor segments with sufficient contrast are considered valid vascular segments (VVS) and are used for analysis. Valid vessel density (VVD) per mm^2^ is then expressed by multiplying 10 μm by the number of VVS identified.

**Figure 2:**
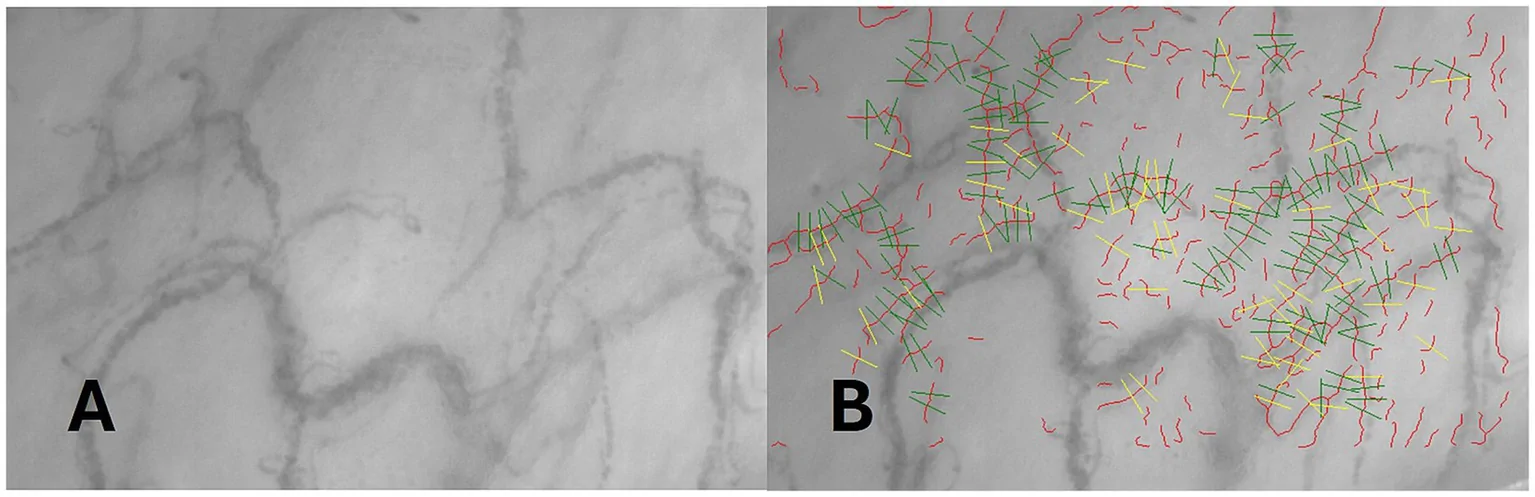
Sidestream dark field videomicroscopy and GlycoCheck™ images of feline sublingual capillaries without **(A)** and with **(B)** marker lines indicating vascular segments. Red wavy lines represent vascular segments, green straight lines indicate valid segments, and yellow straight lines denote invalid segments.

Secondly, RBC column widths are assessed for minimal column width, column position, and signal-to-noise ratio. From the measured column widths, the software then calculates the percentage of RBC filling (%RBC filling) based on the proportion of time a particular VVS contains RBC throughout all recordings per site. The median RBC column width (Median P50) is calculated using the intensity profiles of the dynamic lateral positions of RBCs in all measured column widths.

Finally, a linear regression line is fitted through the obtained Median P50 curve to derive the perfused diameter of vascular segments. From that, the PBR (perfused boundary region), an inverse estimate of EG thickness, is calculated as the distance between the Median P50 and the perfused diameter ([Perfused diameter – median RBC column width]/2). Reported GlycoCheck™ parameters, therefore, include VVD, RBC filling %, Median P50 and PBR for vessels with diameter from 5 to 25 μm and for subgroups of vessels with diameters from 5–9 μm, 10–19 μm and 20–25 μm. Higher PBR is, therefore, an indication for a thinner EG and a lower PBR indicates a thicker EG.

### Packed cell volume and total solids

Packed cell volume and total solids (TS) were measured at each timepoint for each cat using centrifuged capillary microhematocrit tubes and a hand-held refractometer, respectively, by the same operator.

### Statistical analysis

Sample size calculation was based on a study in anesthetized rabbits receiving volume expansion with hypertonic saline (36). The reported mean difference for PBR 5–25 μm was 0.19 μm with reported baseline and post-intervention standard deviation (SD) of 0.30 and 0.18, respectively. As paired-difference data were not available from that study, the SD of the within-subject differences for the present crossover design was estimated using these reported SDs and an assumed within-subject correlation. Using a two-sided α of 0.05 and 80% power, this yielded an estimated sample size of approximately 20 cats.

Triplicates from measured GlycoCheck™ parameters were averaged for further analysis as recommended in previous reports in healthy and critically ill people (37, 38). Data normality was tested using Shapiro–Wilk test and visually inspected using histograms and Q-Q plots (39–41). Normally distributed data is presented as mean ± standard deviation (SD), not normally distributed data is presented as median (Interquartile Range, IQR) (41).

Linear mixed-effects regression modelling was used throughout to evaluate treatment with cat as a random effect (42). GlycoCheck™ parameters were evaluated as dependent variables against group, timepoint and their interaction (model 1.1–1.7), and separately against PCV (model 2.1–2.7). Packed cell volume and TS were similarly evaluated as dependent variables against group, timepoint and their interaction (model 3 and 4, respectively). As a sensitivity analysis, the group x timepoint models for PBR were repeated with PCV included as an additional independent variable (model 5.1–5.4).

Associations between PBR (5–25 μm and the 5–9 μm, 10–19 μm, and 20–25 μm subgroups) and HR, mean arterial pressure (MAP), and temperature were evaluated in separate models (Models 6.1–6.4, 7.1–7.4 and 8.1–8.4, respectively). Finally, HR, systolic arterial pressure, MAP, diastolic arterial pressure, and temperature were evaluated separately against group, timepoint, and their interaction (model 9.1–9.5, respectively). Details for each model including the dependent and independent variables included are summarized in Supplementary Table 1.

Model assumptions were assessed by evaluating normality and heteroscedasticity using the Shapiro–Wilk test and visual inspection of residual histograms. Fixed-effect estimates from the linear mixed-effects models are presented with approximate 95% confidence intervals derived using the Wald method, based on the estimated coefficient ± 1.96 × standard error (42). No formal adjustment for multiple comparisons was applied; model-derived estimates are reported and interpreted in an exploratory context. Outliers were investigated using the interquartile range method, with lower boundary set as Quartile 1 minus 1.5 times IQR and upper boundary – Quartile 3 plus 1.5 times IQR. Modelling was performed with both outliers included and excluded to compare model accuracy and fit.

Correlation between PCV and GlycoCheck™ parameters was calculated using Pearson’s test for normally distributed data and Kendall’s tau for non-normally distributed data. All statistical analyses were conducted in R (Team 2023) (43). The complete dataset, including individual values for all variables at each timepoint, is provided in Supplementary material 1.

## Results

Twenty cats were included in the analysis: nine male neutered, seven female entire, and four female spayed. The median age was 3.21 years (IQR: 1.68), and the mean body weight was 3.77 ± 0.76 kg. Images from a total of 156 timepoints were analyzed. Four image acquisitions in three cats were not completed during the second week due to short-term complications. One cat developed a transient oxygen desaturation during anesthesia at the end of T1 image acquisition. The anesthesia was discontinued and the cat recovered uneventfully, showing no further clinical signs. Two cats experienced IV catheter dislodgment prior to final datapoint collection, and IV catheters could not be replaced. This resulted in an overall success rate of 97%.

Anesthetic variable results and statistical analysis are presented in Supplementary material 2. Group and timepoint had varying effects on anesthetic variables, including a decrease in MAP from baseline at T1. However, values largely remained within acceptable limits for clinical monitoring. One cat experienced hypotension (defined as MAP < 60 mmHg) (44) at T1 while in the VO group. This cat demonstrated consistently slightly lower blood pressure across groups and timepoints and therefore was considered a biological outlier. Hypothermia (defined as T° < 36.7 °C) (45) was recorded in 28% of various timepoints, with the lowest recorded temperature being 34.8 °C. Heart rate was consistently slightly lower than the reported for the species (46). However, this was typical for research cats from this colony and was not considered abnormal. All individual anesthetic variable results can be consulted in Supplementary material 1. Heart rate, MAP and temperature had no significant effect on PBR (Supplementary material 2).

Both sets of models investigating GlycoCheck™ parameters (with and without outliers) yielded similar findings and exhibited consistent overall trends. Models with outliers included were used for reporting results. Inclusion of PCV in Model 1 did not materially alter the group x timepoint effects for any PBR parameter. Summary statistics for GlycoCheck™ parameters are presented in Table 1. A statistical report is presented as Supplementary material 3. None of the analyzed GlycoCheck™ parameters were significantly different at baseline.

**Table 1:** GlycoCheck™ parameters in 20 healthy anesthetized cats subjected to transient volume overload (30 mL/kg Hartman’s solution over 10 min) or control (no intravenous fluids), measured at four timepoints over 24 h.

	Volume overload group				Control group			
	Baseline	10 min	3 h	24 h	Baseline	10 min	3 h	24 h
Parameter	Mean ± SD	Mean ± SD	Mean ± SD	Mean ± SD	Mean ± SD	Mean ± SD	Mean ± SD	Mean ± SD
VVD (mm^2^/mm^2^)	452.96 ± 98.08	405.98 ± 106.42	429.56 ± 100.86	444.63 ± 109.12	466.93 ± 134.28	448.82 ± 87.40	442.03 ± 81.30	463.09 ± 88.81
%RBC filling (%)	0.60 ± 0.07	0.59 ± 0.04	0.59 ± 0.06	0.62 ± 0.06	0.62 ± 0.06	0.59 ± 0.04	0.57 ± 0.06	0.62 ± 0.05
Median P50 (μm)	9.11 ± 1.52	8.95 ± 1.28	8.49 ± 1.07	8.41 ± 1.06	8.69 ± 1.53	8.91 ± 1.38	8.73 ± 1.39	8.24 ± 1.33
PBR 5–25 (μm)	2.58 ± 0.24	2.62 ± 0.11	2.64 ± 0.17	2.46 ± 0.18	2.48 ± 0.21	2.55 ± 0.19	2.63* ± 0.13	2.48 ± 0.20
PBR 5–9 (μm)	1.40 ± 0.13	1.38 ± 0.12	**1.38**^**§**^ **± 0.09**	**1.29**^**§**^ **± 0.14**	1.34 ± 0.15	1.38 ± 0.10	1.42* ± 0.10	1.36 ± 0.09
PBR 10–19 (μm)	2.93 ± 0.28	2.99 ± 0.13	3.00 ± 0.17	2.79 ± 0.20	2.83 ± 0.23	2.92 ± 0.21	3.00 ± 0.15	2.83 ± 0.23

	Median (IQR)	Median (IQR)	Median (IQR)	Median (IQR)	Median (IQR)	Median (IQR)	Median (IQR)	Median (IQR)
PBR 20–25 (μm)	3.03 (0.30)	3.05 (0.32)	3.22 (0.29)	2.98 (0.44)	2.95 (0.41)	2.99 (0.44)	3.18 (0.24)	2.87 (0.64)
PCV (%)	34.00 (3.50)	25.00^*§^ **(3.00)**	31.00^*§^ **(5.50)**	35.00^*^ **(2.75)**	33.00 (4.25)	29.00^*^ **(5.00)**	34.00^*^ **(3.00)**	35.00^*^ **(4.50)**
TS (g/L)	70.00 *(8.00)	55.00^*§^ **(6.50)**	67.00^*§^ **(4.50)**	70.00^*^ **(6.00)**	70.00 (6.00)	67.50^*^ **(6.00)**	73.00^*^ **(4.50)**	70.00^*^ **(6.00)**

IQR, interquartile range; PBR, perfused boundary region; PCV, packed cell volume; RBC, red blood cells; SD, standard deviation; TS, total solids; VVD, valid vessel density. Values in bold represent statistically significant results (*p* < 0.05).

^*^Statistically significant time effects.

^§^Statistically significant time x group interaction effect.

Perfused boundary region trended upward over time, with apparent peak values at T2 (3 h post-intervention). Mixed-effects modeling identified a significant effect of time for PBR 5–25 μm, PBR 5–9 μm and PBR 10–19 μm. For PBR 5–25 μm and PBR 10–19 μm model estimate values at T2 were higher relative to baseline (estimate 0.150, 95% CI 0.037 to 0.264, *p* = 0.001 and estimate 0.175, 95% CI 0.047 to 0.303, *p* = 0.008, respectively).

For PBR 5–9 μm, a significant effect of time was also observed, with increased values at T2 relative to baseline (estimate 0.0887, 95% CI 0.016 to 0.161, *p* = 0.017). However, significant group x timepoint interactions were identified at T2 (estimate −0.116, 95% CI −0.219 to −0.013, *p* = 0.028) and T3 (estimate −0.138, 95% CI −0.0242 to −0.033, *p* = 0.010), indicating that this time-dependent increase was attenuated in the VO group, resulting in lower PBR 5–9 μm compared to the control group at these timepoints. Group and timepoint interactions for PBR are illustrated in Figure 3. Individual PBR values for each group and timepoint are presented as Supplementary material 1. Individual trajectories for PBR 5–25 μm are presented in Figure 4 and for PBR 5–9, 10–19 and 20–25 μm as Supplementary material 4.

**Figure 3:**
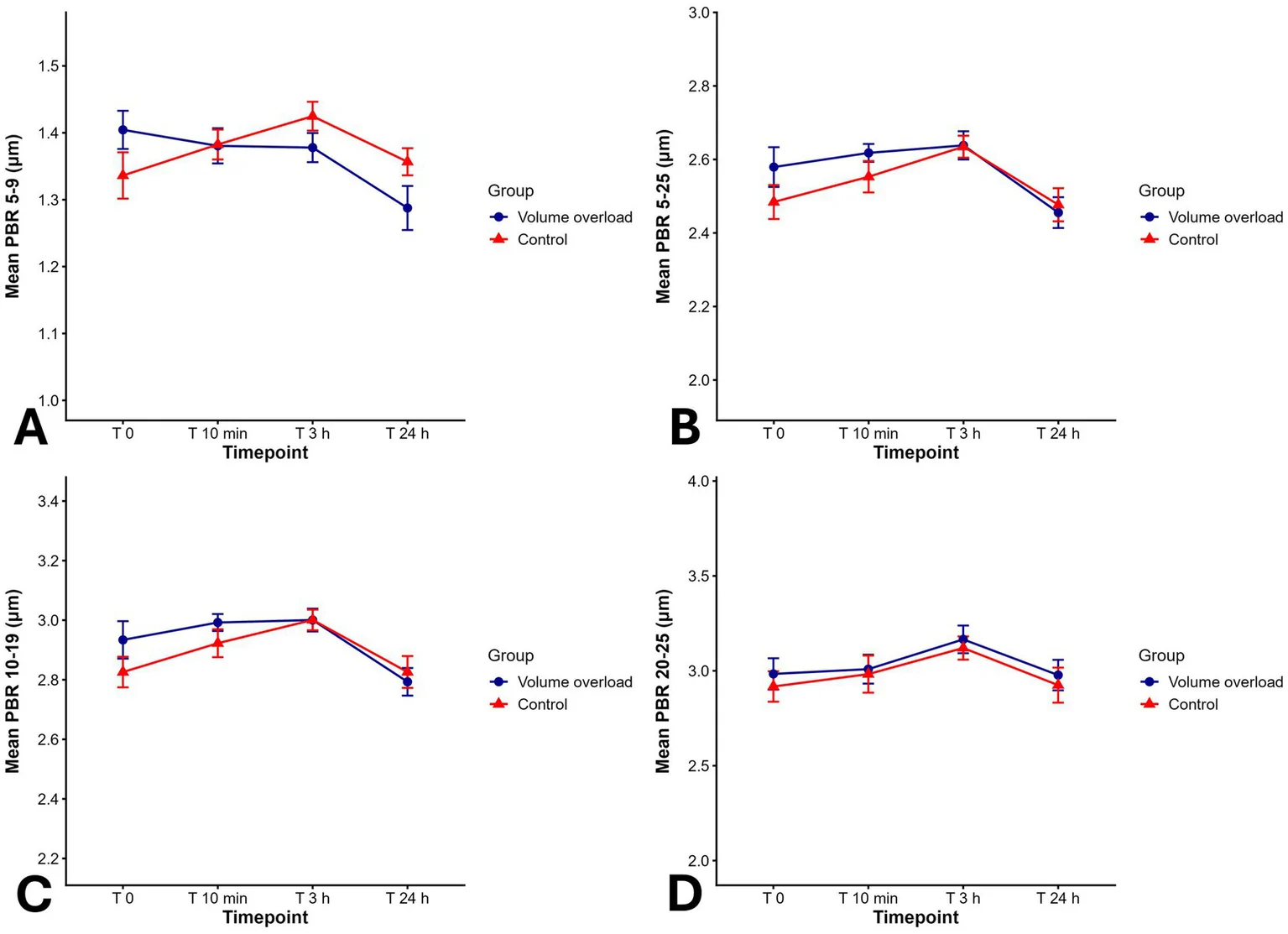
Changes in perfused boundary regions over time in vessels with diameters of 5–25 μm **(A)**, 5–9 μm **(B)**, 10–19 μm **(C)**, and 20–25 μm **(D)** in 20 healthy anesthetized cats with transient volume overload (navy circles) or control (red triangles), measured at four timepoints over 24 h: T0 (baseline), T1 (10 min), T2 (3 h), and T3 (24 h). Symbols represent mean values, with error bars indicating standard errors. The lower and upper limit of the y-axis represent established tolerance intervals for the species (28).

**Figure 4:**
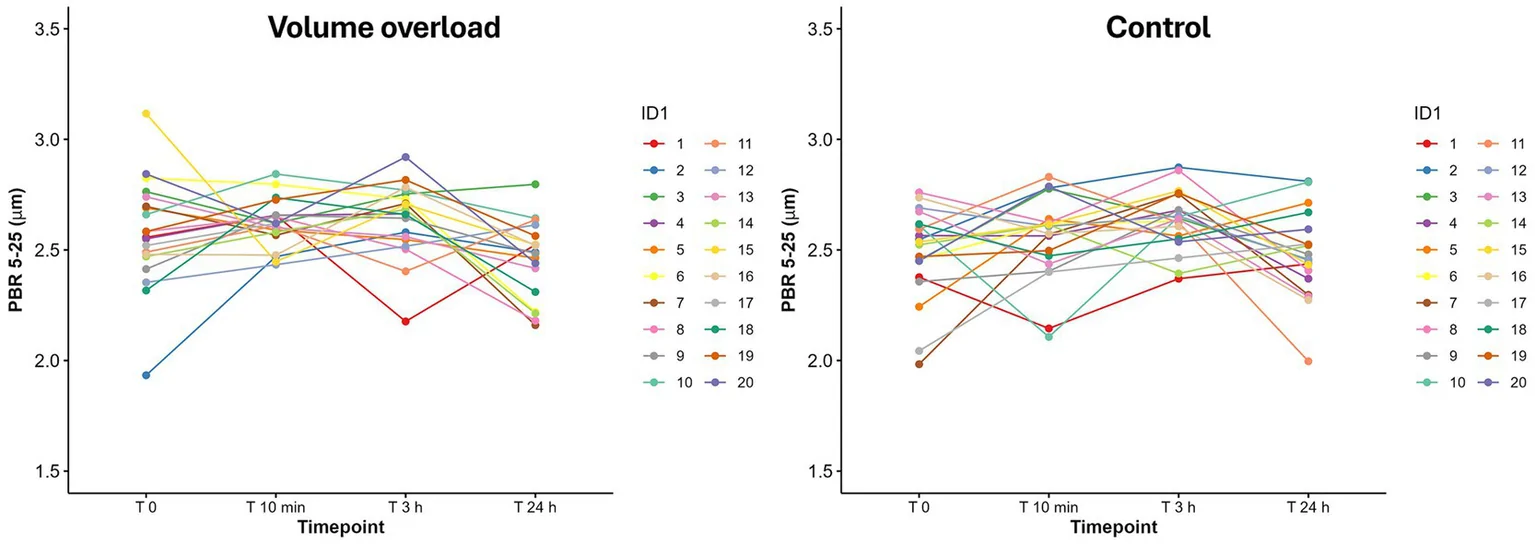
Individual changes in perfused boundary regions over time in vessels with diameters of 5–25 μm in 20 healthy anesthetized cats with transient volume overload or control.

The median (IQR) count for major (10 μm) vascular segments with diameters 5–25 μm was 1520.05 (356.50). Within this range, median counts for the 5–9 μm, 10–19 μm, and 20–25 μm subgroups were 910.32 (348.75), 566.91 (172.83), and 42.82 (33.83), respectively (Figure 5).

**Figure 5:**
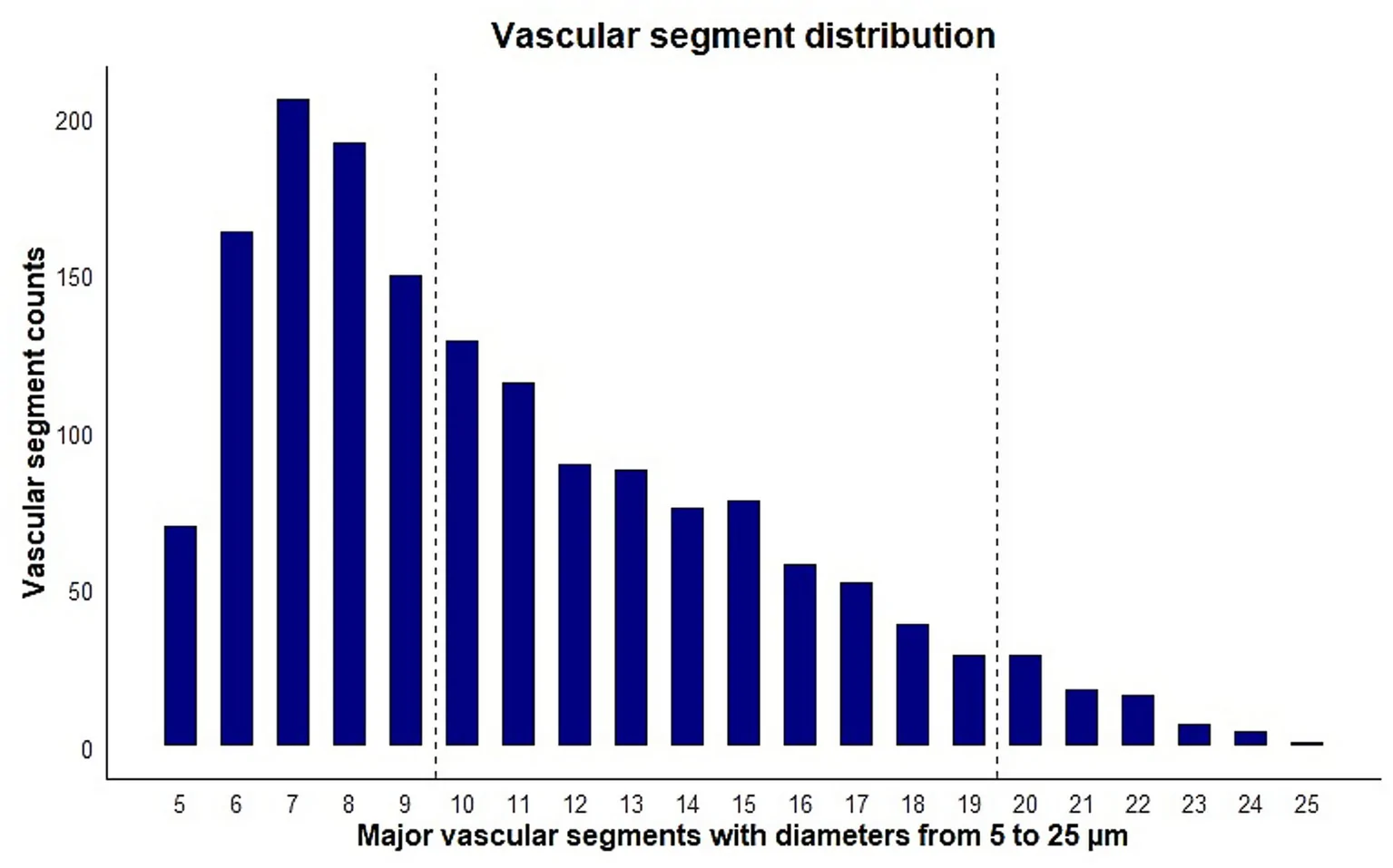
Distribution of major vascular segment counts by vessel diameter (5–25 μm) in healthy anesthetized cats. Bars represent the number of valid vascular segments identified per diameter subgroup using GlycoCheck™ analysis. Vertical dashed lines separate the predefined vessel size categories: 5–9 μm, 10–19 μm, and 20–25 μm.

Packed cell volume fluctuated significantly over time, with mixed effects modeling identifying a significant effect of timepoint. Relative to baseline, model-estimated PCV was lower at T1 (estimate −3.350, 95% CI −4.863 to −1.837, *p* < 0.001), and higher at T2 (estimate 1.950, 95% CI 0.437 to 3.463, *p* = 0.012) and T3 (estimate 2.234, 95% CI 0.699 to 3.767, *p* = 0.005). Significant group x timepoint interactions were identified at T1 (estimate −4.200, 95% CI −6.339 to −2.061, *p* < 0.001) and T2 (estimate −2.554, 95% CI −4.709 to −0.399, *p* = 0.021), indicating lower PCV in the VO group compared to the control group at these timepoints.

Similarly, TS varied over time, with mixed-effects modelling identifying a significant effect of timepoint. Relative to baseline, model-estimated TS was lower at T1 (estimate −2.200, 95% CI −4.292 to −0.108, *p* = 0.04) and higher at T2 (estimate 2.600, 95% CI 0.508 to 4.692, *p* = 0.016). Significant group x timepoint interactions were identified at T1 (estimate −12.450 95% CI −15.409 to −9.491, *p* < 0.001) and T2 (estimate −5.812, 95% CI −8.793 to −2.831, *p* < 0.001), indicating lower TS values in the VO group compared to the control group at these timepoints.

Packed cell volume showed positive associations with VVD (estimate 5.747, 95% CI 1.956 to 9.537, *p* = 0.003) and %RBC filling (estimate 0.004, 95% CI 0.002 to 0.006, *p* < 0.001), and negative associations with PBR 5–25 μm (estimate −0.011, 95% CI –0.018 to −0.004, *p* = 0.003) and PBR 10–19 μm (estimate −0.013, 95% CI −0.021 to −0.005, *p* = 0.001). Correlation coefficients for PCV and GlycoCheck™ parameters are presented in Table 2 and, although statistically significant, were overall weak. Full fixed-effect results from all linear mixed-effects models are presented as Supplementary material 5.

**Table 2:** Correlation between packed cell volume and GlycoCheckTM parameters using Pearson’s test (except for perfused boundary region 20–25 μm).

Parameter	*r*	95% CI	*p –* value
VVD	0.235	0.081—0.378	**0.003**
%RBC filling	0.277	0.126—0.416	**< 0.001**
Median P50	−0.071	−0.225 — 0.088	0.382
PBR 5–25 μm	−0.238	−0.381 — −0.0841	**0.003**
PBR 5–9 μm	−0.102	−0.255 — 0.056	0.203
PBR 10–19 μm	−0.255	−0.396 — −0.102	**0.001**
PBR 20–25 μm	τ = −0.111	-	**0.048**

CI, confidence interval; Median P50, median red blood cell column diameter; PBR, perfused boundary region; RBC, red blood cells; VVD, valid vessel density.

*Correlation calculated using Kendall’s tau method for non-parametric data. Values in bold represent statistically significant results (*p* < 0.05).

## Discussion

Results from this study suggest that rapid IV administration of 30 mL/kg of buffered balanced crystalloid solution does not decrease estimated EG thickness in healthy cats. However, the PBR 5–25 μm, PBR 5–9 μm and PBR 10–19 μm, increased 3 h after intervention, suggesting a transient decrease in EG thickness potentially secondary to anesthetic effects. Interestingly, the PBR of small vessels (5–9 μm) was lower over time in the VO group at later timepoints, suggesting these effects might have been mitigated by the administration of IV fluids.

Transient volume overload did not result in increases in PBR in this group of healthy cats. This aligns with findings from a previous study in healthy cats from our group, which showed no increase in PBR following a fluid challenge (10 mL/kg over 20 min) using either Hartman’s solution or 6% hydroxyethyl starch 130/0.4 (30). Similar results have been reported in anesthetized rabbits, where only a hypertonic saline bolus (5 mL/kg of 10% NaCl) increased PBR, while volume loading with Lactated Ringer’s solution (30 mL/kg/h) had no effect (17, 36).

In canine haemorrhagic shock models, PBR generally remained unchanged during haemorrhage and autotransfusion in the absence of IV fluids (47). However, one study showed increased PBR following resuscitation with 30 mL/kg of LRS or 7.5 mL/kg of hydroxyethyl starch compared to natural or synthetic blood products (13). Likewise, in septic children, fluid boluses with unbalanced versus balanced crystalloids led to PBR increases 6 h post-intervention (48). Taken together, these studies suggest that IV fluids alone may not alter PBR in healthy animals, while more severe interventions (e.g., haemorrhage) or underlying disease states (e.g., sepsis) may predispose to detectable EG alterations. Nevertheless, differences in study design, species, and methodology limit direct comparison or translational conclusions.

The PBR 5–25 μm, 5–9 μm and 10–19 μm increased over time in the control group and returned to baseline by 24 h in this study. These changes cannot be attributed to IV fluid-associated VO. Therefore, alternative explanations should be considered. Anesthesia-related changes in blood volume are well documented in the literature and primarily attributed to fluid shifts from the interstitial into the IV space in response to anesthesia-induced hypotension (18, 49, 50). In addition, most anesthetics, including propofol, cause splenic relaxation in dogs and cats, leading to sequestration of RBC and transient reductions in PCV (20, 51–55). In the present study both PCV and TS decreased transiently across both groups, supporting anesthesia-related changes in blood volume, particularly during the initial longer anesthetic period (T0 and T1 combined).

Whether such changes in blood volume could be associated with EG damage remains uncertain. A recent study examining plasma volume changes in hypotensive versus normotensive human surgery patients demonstrated a tenfold increase in plasma volume in the hypotensive group. However, this was not associated with increased NP production or EG shedding (18), suggesting that hypotension-induced volume expansion does not damage the EG. More importantly, although MAP decreased at T1 in the present study, only one cat experienced mild hypotension. Therefore, anesthesia-related hypotension and its associated plasma volume expansion are unlikely to explain the observed changes in PBR.

More broadly, hemodynamic anesthetic variables (HR, MAP and temperature) were not associated with PBR, consistent with previous studies in healthy cats using similar anesthetic protocols (28, 30). Nonetheless, neither study was designed to investigate anesthesia-related effects. Blood pressure was monitored non-invasively primarily for anesthetic safety. Proper exploration of hemodynamic anesthetic effects on the EG would require a more targeted setup, including direct blood pressure measurement, cardiovascular point-of-care ultrasound, and collection of additional hemodynamic parameters.

Another anesthesia-related factor worth considering is the choice of anesthetic agent itself, particularly propofol, which may have direct effects on the endothelium and its glycocalyx. Studies using SDFV have reported effects of propofol on the microcirculation in people, generally showing trends toward decreased perfused vascular density and other measures of microcirculatory function over time or compared with other sedatives and anesthetics (56–60). It is therefore possible that propofol may also affect the feline microcirculation and consequently influence estimated EG thickness. However, these studies included heterogeneous cohorts, predominantly critically ill patients, and varied substantially in study design, precluding direct comparison with the present study. Furthermore, they did not assess estimated EG thickness, as the methodologies used did not support a PBR calculation.

Outside of microcirculatory imaging, both *in vitro* and *in vivo* experimental studies have demonstrated endothelial cell damage and EG shedding after propofol overdose (61–63). In contrast, clinical studies comparing the effects of propofol and volatile anesthetics in people have not consistently demonstrated increased EG shedding following propofol administration (64–66). These effects appear to be dose-dependent and time-related, and confounding factors such as underlying EG-damaging illnesses limit the conclusions that can be drawn from clinical data.

Propofol has been used in previous studies evaluating PBR in cats (28, 30), though these studies did not include repeated propofol administration within 3 h. Namely, in a previous study from our group evaluating effects of an IV fluid challenge on GlycoCheck™ parameters in healthy cats, increases in PBR 10–19 μm were found at the end of infusion, compared to baseline (30), suggesting comparable effects. Considering the relative feline “sensitivity” to propofol-related oxidative stress (67–69), longer half-life and slower metabolism of phenolic compounds in cats (51), it is possible that propofol may have contributed to a transient reduction in EG thickness, especially considering the repeated administrations between T0 and T2. Further studies comparing effects of repeated administration and use of different anesthetics are necessary to investigate this possibility.

Surprisingly, the PBR 5–9 μm was lower at later timepoints (T2 and T3) in the VO group. It is possible that IV fluid administration mitigated some of the negative effects, specifically in the smallest analyzed vessels. A previous study from our group, investigating the immediate effects of an IV fluid challenge using Hartman’s solution or hydroxyethyl starch in healthy cats similarly reported decreases in PBR in the hydroxyethyl starch group, though in larger vessels (10–19 μm) (30). This may have resulted from dilution of anesthetic agents or potentially from “rehydrating” the EG. Further studies specifically designed to investigate anesthetic influences on the EG are warranted to support this hypothesis.

This study demonstrated weak associations between PCV and GlycoCheck™ parameters. Associations between PCV and PBR have been previously found in adult and neonate human patients (70, 71). These findings are broadly consistent with a previous study from our group, which evaluated the effects of a fluid challenge on GlycoCheck™ parameters in cats and found that VVD and %RBC filling decreased and PBR increased with decreasing PCV (30).

Although these associations are weak, they warrant further investigation given that all GlycoCheck™ parameters rely on identification of RBCs against a contrasting background, with PBR calculations dependent on the presence of vessels meeting criteria for “valid” density and adequate % RBC filling (31). If a consistent influence of PCV on PBR is confirmed with specifically designed studies, adjustment for PCV may be warranted when interpreting PBR measurements, particularly in clinical research.

Interestingly, most GlycoCheck™ parameters returned to baseline by 24 h, suggesting that any EG disturbance was either transient or rapidly resolved. However, this contrasts with current understanding of EG shedding, turnover, and repair (72). While EG shedding can occur rapidly (within minutes to hours) following a significant stimulus, the biosynthesis of new components typically requires several hours to days, depending on the molecule and mechanism involved (72, 73). Therefore, the return to baseline at 24 h may represent dimensional recuperation or functional rehydration, rather than full structural restoration. It is conceivable that IV fluids and anesthetic stimuli caused a transient EG “desiccation” or “contraction,” rather than substantial loss of EG components. Although incomplete structural recovery could theoretically challenge the adequacy of the 7-day washout period, normalization of GlycoCheck™ measurements within 24 h makes a meaningful carryover effect unlikely. Unfortunately, EG shedding biomarkers were not assessed in this study. Given their limitations in detecting fluid-induced changes to the EG, the reliability of such additional analyses remains uncertain.

In considering the potential stimulus for EG change, the selected IV fluid dose warrants further reflection. Beyond representing empirically “high” IV fluid rate, the dose chosen for this VO model was based on a previous study evaluating cardiac ultrasound and caudal vena cava parameters in healthy cats with induced VO (74). In the referenced study, rapid infusion of 30 mL/kg of Hartman’s solution resulted in a mild but statistically significant increase in cardiac chamber and caudal vena cava size, suggesting cardiac stretch on imaging. However, whether this stretch was sufficient to trigger NP production remains unclear. By comparison, a canine study using a much higher overall dose over a longer time (90 mL/kg of isotonic crystalloids over 1 h) demonstrated marked increases in NP (75), confirming that IV fluids can stimulate NP release under certain conditions. Considering the feline predisposition to volume overload, the highest fluid rate considered ethically and physiologically acceptable was used to potentially affect the EG without causing harm. However, NP concentrations were not measured in this study, so any involvement of NP in the EG modulation could not be confirmed. Further investigations should incorporate species-specific volume kinetic data to guide fluid dosing strategies in cats for studies related to microcirculation or other IV fluid research (25, 76).

Although not statistically significant, there was notable variability in PBR measurements in this study, especially at baseline, despite the cross over design and averaging three measurements as per current recommendations in people (37, 38). This is consistent with previous findings in a large cohort of healthy cats (28). Such variability may reflect both biological variation and limitations associated with applying the GlycoCheck™ system to feline microcirculation. The software was developed for human use and has not been specifically adapted for use in cats, therefore species-specific characteristics of the feline microcirculation may influence some aspects of its derived parameters. Differences in capillary bed density have been reported across mammalian species (77) including studies using SDFV to assess perfused vessel density in cats, dogs, and horses (78–81). Subjectively, cats also appear to have less dense capillary beds when examined using SDFV (personal observation). Importantly, VVD has been reported to be considerably lower in cats compared to dogs and people (28, 31, 82). As VVD is a source parameter used in the calculation of PBR, lower vessel density may reduce the number of datapoints available for analysis, potentially increasing measurement ‘noise’ and contributing to PBR variability.

Intra- and inter-individual variability in GlycoCheck™ measurements is not inherent to cats. As discussed above, variability has also been specifically investigated in healthy and critically ill people (37, 38, 83). Although averaging multiple measurements improves reproducibility, it does not eliminate variability. Inter-operator variability has also been reported, although some studies have demonstrated acceptable transferability between operators (83, 84).

Beyond measurement variability, GlycoCheck™ has several methodological limitations. Although image analysis is automated, image acquisition remains operator dependent and susceptible to factors such as pressure artefacts. Measurements may also be influenced by physiological and examination conditions (38), highlighting the importance of standardized acquisition protocols. Differences between software versions may limit direct comparison between studies (38). Importantly, PBR is a surrogate of EG thickness rather than a direct measurement of EG structural integrity. It therefore does not directly quantify loss of structural components or more importantly assess EG function. Some of these limitations are not specific to GlycoCheck™ but are inherent to sublingual microcirculatory imaging using SDFV and should be considered when interpreting and comparing findings obtained using this methodology (85–87).

Collectively, these limitations suggest that evaluating individual longitudinal changes in PBR may be challenging in feline patients using the current version of the software. Nevertheless, the GlycoCheck™ may remain useful for investigating group-level changes in estimated EG thickness, provided these limitations are acknowledged, particularly given the lack of alternative methods for non-invasive investigation of the EG in cats.

Additional limitations in this study include the relatively small sample size and somewhat arbitrary timepoints. Notably, T0 and T1 measurements were obtained during the same anesthetic episode, whereas separate, shorter anesthesia was used at T2 and T3, potentially resulting in differences in the physiological context between measurements. The T0–T1 anesthesia was the longest of the study. However, its duration remained relatively short (~20 min). Furthermore, no relevant changes in PBR were observed at T1, the timepoint most likely to have been affected by the longer anesthetic duration.

Butorphanol and gabapentin, selected for their mild sedative and cardiovascular effects were administered primarily to facilitate IV catheter placement, and therefore not repeated at T2 and T3, although residual gabapentin effects may have persisted at T2. As their effects on the feline microcirculation are unknown, differences in their administration between timepoints represent a potential confounding factor that cannot be excluded.

Lastly, this was a small-scale experimental study conducted in the context of limited prior data in this species, with much of the existing evidence originating from the same research group. The findings should therefore be interpreted as exploratory, providing a basis for further investigation and independent replication rather than definitive conclusions or direct clinical application.

## Conclusion

This study did not demonstrate decreases in estimated EG thickness after transient, mild VO. However, changes in PBR over time may suggest potential anesthesia-related effects. As short anesthesia is inevitably required for SDFV imaging in small animals, further studies specifically designed to optimize anesthetic protocols are essential. Changes in PCV were associated with alterations in GlycoCheck™ parameters, and the clinical significance of these associations warrants further investigation. Given the known predisposition of cats to VO, the effects of IV fluids on the feline microcirculation should continue to be explored, ideally through study designs informed by a more detailed understanding of volume kinetics in this species.

## Data Availability

The raw data supporting the conclusions of this article is available in Supplementary material.
