# Supplementary material for: Effects of transient intravascular volume overload on endothelial glycocalyx thickness in healthy anesthetized cats
Source: Front Vet Sci. 2026 Oct 14;13:1742967. doi: 10.3389/fvets.2026.1742967 (PMC13713276; doi:10.3389/fvets.2026.1742967)
Supplement: Supplementary file 1 [file Data_Sheet_1.PDF]

Supplementary Material - Anesthetic variables analysis

Table 1. Anesthetic variables per group and timepoint in 20 cats with transient intravascular volume overload or control

Variable	Baseline				10 min				3 h				24 h			
	n	Median	Min-Max	Mean ± SD	n	Median	Min-Max	Mean ± SD	n	Median	Min-Max	Mean ± SD	n	Median	Min-Max	Mean ± SD
**Volume overload group**																
**HR**	20	110	89-145	115.78 ± 16.84	19	117	95-142.67	118.48 ± 15.37	19	131.5	106.5-159.5	132.02 ± 15.84	18	140.67	110.5-189	141.96 ± 18.28
**SAP**	17	110	85-131.5	110.53 ± 11.9	20	105	82.5-170	113.02 ± 18.47	19	115	98.5-142	116.25 ± 11.98	18	124	109-152.5	125.38 ± 10.39
**MAP**	17	85	65-102.5	83.03 ± 11.87	20	75	54-97	77.14 ± 11.53	19	80	66-111.5	81.98 ± 10.61	18	87.5	76-105	88.02 ± 9.2
**DAP**	17	65	45-90	67.15 ± 13.69	20	55	40-78.33	57.83 ± 11.19	19	65	48-96.5	64.98 ± 11.39	18	65.75	51.67-90.5	69.31 ± 11.22
**Temp**	19	37.2	36-38.1	37.04 ± 0.6	19	36.6	34.8-38.1	36.55 ± 0.87	17	37.6	36.2-39	37.51 ± 0.82	18	37.7	36.57-38.6	37.64 ± 0.47
**Control group**																
**HR**	20	117.17	85-160	116.13 ± 17.66	20	110.75	87.5-136	112.06 ± 14.29	20	127.08	98.5-159.5	127.31 ± 16.46	19	147	112.3-177.5	147.39 ± 17.4
**SAP**	20	117.75	95-160	121.47 ± 17.19	20	110	98-128.5	112.32 ± 8.39	20	113.75	93.5-137.5	114.83 ± 12.22	19	129	102.5-169	127.81 ± 15.78
**MAP**	20	86.5	70-118	88.73 ± 11.53	20	77.25	67.5-95	78.4 ± 7.84	20	80.25	62-107.5	81.68 ± 11.02	19	86	70.5-123	90.59 ± 14.36
**DAP**	20	67.5	45-105	71.58 ± 14.64	20	61.67	48.75-95	63.68 ± 12.32	20	62.5	46-95	65.38 ± 12.21	19	70	50.5-105.5	71.96 ± 14.89
**Temp**	20	36.8	35.3-37.6	36.8 ± 0.6	20	36.45	34.9-37.6	36.51 ± 0.79	19	37.4	35.7-38.2	37.21 ± 0.78	19	37.6	36-39	37.51 ± 0.68

DAP – diastolic arterial pressure; HR – heart rate; MAP – mean arterial pressure; n – number of observations; SAP – systolic arterial pressure; Temp – rectal temperature;

Statistical analysis results

When assessing the dataset for normality, some deviations were identified by Shapiro–Wilk testing. However, graphical assessment using histograms and Q–Q plots suggested that these were minor.

No significant effects of HR, MAP, or Temp on any PBR were identified by mixed effects modelling.

Mixed effects modelling demonstrated varying effects of group and timepoint on anesthetic variables. Relative to baseline, model-estimated HR was higher at T2 (estimate 11.18, 95% CI 2.84 to 19.51, $p = 0.010$) and T3 (estimate 32.18, 95% CI 23.72 to 40.63, $p < 0.001$). At baseline, model-estimated SAP was lower in the VO group compared with the control group (estimate −11.02, 95% CI -19.59 to -2.46, $p = 0.013$). Relative to baseline, SAP was lower at T1 (estimate -9.15, 95% CI -17.34 to -0.95, $p = 0.030$). A significant group x timepoint interaction was identified at T2 (estimate 12.56, 95% CI 0.64 to 24.48, $p = 0.041$), indicating that the change in SAP from baseline to T2 differed between groups. Relative to baseline, model-estimated MAP was lower at T1 (estimate -10.33, 95% CI -16.60 to -4.07, $p = 0.002$) and T2 (estimate -7.05, 95% CI -13.31 to -0.79, $p = 0.029$). Relative to baseline, model-estimated DAP was lower at T1 (estimate -7.90, 95% CI -14.91 to -0.90, $p = 0.029$). Relative to baseline, model-estimated temperature was higher at T2 (estimate 0.43, 95% CI 0.03 to 0.82, $p = 0.037$) and T3 (estimate 0.70, 95% CI 0.31 to 1.10, $p < 0.001$).
