# Supplementary material for: Effects of transient intravascular volume overload on endothelial glycocalyx thickness in healthy anesthetized cats
Source: Front Vet Sci. 2026 Oct 14;13:1742967. doi: 10.3389/fvets.2026.1742967 (PMC13713276; doi:10.3389/fvets.2026.1742967)
Supplement: Supplementary file 2 [file Data_Sheet_2.PDF]

Unformatted statistical report
Effects of transient intravascular volume overload on endothelial glycocalyx thickness in healthy anaesthetized cats

GlycoCheck™ parameters - Time and Group interactions

1. Outliers included

```
> library(nlme)
> library(readxl)
> VO_combined_data$Group <- relevel(as.factor(VO_combined_data$Group), ref
= "2")
> VO_combined_data$Timepoint <- as.factor(VO_combined_data$Timepoint)
>
```

Density - VVD

```
> mixed_model_density <- lme(density ~ Timepoint * Group, random = ~1 | ID
1, data = VO_combined_data)
> summary(mixed_model_density)

Linear mixed-effects model fit by REML
  Data: VO_combined_data
       AIC      BIC    logLik
  1832.401 1862.373 -906.2005

Random effects:
 Formula: ~1 | ID1
        (Intercept) Residual
StdDev:    15.63678   100.76

Fixed effects:  density ~ Timepoint * Group
                    Value Std.Error  DF   t-value p-value
(Intercept)      466.9333  22.80032 129 20.479241  0.0000
Timepoint2       -18.1167  31.86312 129 -0.568578  0.5706
Timepoint3       -24.9000  31.86312 129 -0.781468  0.4360
Timepoint4        -4.2469  32.28818 129 -0.131531  0.8956
Group1           -13.9750  31.86312 129 -0.438595  0.6617
Timepoint2:Group1 -28.8583  45.06126 129 -0.640424  0.5230
Timepoint3:Group1   1.8452  45.36295 129  0.040676  0.9676
Timepoint4:Group1  -3.8867  45.99444 129 -0.084503  0.9328
 Correlation: 
                  (Intr) Tmpnt2 Tmpnt3 Tmpnt4 Group1 Tm2:G1 Tm3:G1
Timepoint2        -0.699                                          
Timepoint3        -0.699  0.500                                   
Timepoint4        -0.690  0.493  0.493                            
Group1            -0.699  0.500  0.500  0.493                     
Timepoint2:Group1  0.494 -0.707 -0.354 -0.349 -0.707              
Timepoint3:Group1  0.491 -0.351 -0.702 -0.347 -0.702  0.497       
Timepoint4:Group1  0.484 -0.346 -0.346 -0.702 -0.693  0.490  0.487

Standardized Within-Group Residuals:
        Min          Q1         Med          Q3         Max 
-2.99293378 -0.56827922 -0.01485611  0.67390426  2.40367389 

Number of Observations: 156
Number of Groups: 20


> residuals_density <- resid(mixed_model_density)
> hist(residuals_density, breaks = 20, main = "Histogram of Model Residual
s - Density",
+      xlab = "Residuals", col = "skyblue", border = "black")
> print(shapiro.test(residuals_density))

	Shapiro-Wilk normality test
```

```
data:  residuals_density
W = 0.98843, p-value = 0.2258


> #CI
> intervals(mixed_model_density, which = "fixed", level = 0.95)
Approximate 95% confidence intervals

 Fixed effects:
                        lower        est.     upper
(Intercept)         421.82233 466.933333 512.04433
Timepoint2          -81.15864 -18.116667  44.92531
Timepoint3          -87.94197 -24.900000  38.14197
Timepoint4          -68.12986  -4.246897  59.63606
Group1              -77.01697 -13.975000  49.06697
Timepoint2:Group1  -118.01314 -28.858333  60.29648
Timepoint3:Group1   -87.90650   1.845205  91.59691
Timepoint4:Group1   -94.88780  -3.886661  87.11448
```

% RBC filling

```
> mixed_model_rbcfilling <- lme(rbcfilling ~ Timepoint * Group, random = ~
1 | ID1, data = VO_combined_data)
> summary(mixed_model_rbcfilling)

Linear mixed-effects model fit by REML
  Data: VO_combined_data
        AIC       BIC   logLik
  -396.0863 -366.1141 208.0431

Random effects:
 Formula: ~1 | ID1
         (Intercept)   Residual
StdDev: 2.445572e-06 0.05475434

Fixed effects:  rbcfilling ~ Timepoint * Group
                       Value  Std.Error  DF   t-value p-value
(Intercept)        0.6254083 0.01224344 129 51.08108  0.0000
Timepoint2        -0.0287083 0.01731484 129 -1.65802  0.0997
Timepoint3        -0.0496417 0.01731484 129 -2.86700  0.0048
Timepoint4        -0.0100750 0.01754119 129 -0.57436  0.5667
Group1            -0.0251000 0.01731484 129 -1.44962  0.1496
Timepoint2:Group1  0.0152000 0.02448689 129  0.62074  0.5359
Timepoint3:Group1  0.0343684 0.02464746 129  1.39440  0.1656
Timepoint4:Group1  0.0304333 0.02498305 129  1.21816  0.2254
 Correlation:
                  (Intr) Tmpnt2 Tmpnt3 Tmpnt4 Group1 Tm2:G1 Tm3:G1
Timepoint2        -0.707
Timepoint3        -0.707  0.500
Timepoint4        -0.698  0.494  0.494
Group1            -0.707  0.500  0.500  0.494
Timepoint2:Group1  0.500 -0.707 -0.354 -0.349 -0.707
Timepoint3:Group1  0.497 -0.351 -0.703 -0.347 -0.703  0.497
Timepoint4:Group1  0.490 -0.347 -0.347 -0.702 -0.693  0.490  0.487

Standardized Within-Group Residuals:
        Min          Q1         Med          Q3         Max
-2.37488163 -0.68852984 -0.03629849  0.49448131  3.01163316

Number of Observations: 156
Number of Groups: 20
>
> residuals_rbcfilling <- resid(mixed_model_rbcfilling)
> hist(residuals_rbcfilling, breaks = 20, main = "Histogram of Model Resid
uals - RBC Filling",
+      xlab = "Residuals", col = "skyblue", border = "black")
> print(shapiro.test(residuals_rbcfilling))

       Shapiro-Wilk normality test
```

```
data:  residuals_rbcfilling
W = 0.97786, p-value = 0.01302

#CI
> intervals(mixed_model_rbcfilling, which = "fixed", level = 0.95)
Approximate 95% confidence intervals

 Fixed effects:
                         lower        est.        upper
(Intercept)         0.60118438  0.62540833  0.649632287
Timepoint2         -0.06296618 -0.02870833  0.005549510
Timepoint3         -0.08389951 -0.04964167 -0.015383824
Timepoint4         -0.04478068 -0.01007500  0.024630677
Group1             -0.05935784 -0.02510000  0.009157843
Timepoint2:Group1 -0.03324791  0.01520000  0.063647906
Timepoint3:Group1 -0.01439718  0.03436842  0.083134022
Timepoint4:Group1 -0.01899625  0.03043333  0.079862918
```

Median P50

```
> mixed_model_medianp50 <- lme(medianp50 ~ Timepoint * Group, random = ~1
| ID1, data = VO_combined_data)
> summary(mixed_model_medianp50)
Linear mixed-effects model fit by REML
  Data: VO_combined_data
       AIC      BIC    logLik
  536.0327 566.0048 -258.0164

Random effects:
 Formula: ~1 | ID1
        (Intercept) Residual
StdDev:    0.609075 1.188552

Fixed effects:  medianp50 ~ Timepoint * Group
                      Value Std.Error  DF   t-value p-value
(Intercept)        8.686000 0.2986326 129 29.085910  0.0000
Timepoint2         0.220500 0.3758532 129  0.586665  0.5585
Timepoint3         0.045833 0.3758532 129  0.121945  0.9031
Timepoint4        -0.416029 0.3812183 129 -1.091314  0.2772
Group1             0.420917 0.3758532 129  1.119897  0.2648
Timepoint2:Group1 -0.381417 0.5315366 129 -0.717574  0.4743
Timepoint3:Group1 -0.619370 0.5353764 129 -1.156887  0.2495
Timepoint4:Group1 -0.220011 0.5434062 129 -0.404874  0.6862
 Correlation:
                  (Intr) Tmpnt2 Tmpnt3 Tmpnt4 Group1 Tm2:G1 Tm3:G1
Timepoint2        -0.629
Timepoint3        -0.629  0.500
Timepoint4        -0.620  0.493  0.493
Group1            -0.629  0.500  0.500  0.493
Timepoint2:Group1  0.445 -0.707 -0.354 -0.349 -0.707
Timepoint3:Group1  0.442 -0.351 -0.702 -0.346 -0.702  0.496
Timepoint4:Group1  0.435 -0.346 -0.346 -0.702 -0.692  0.489  0.487

Standardized Within-Group Residuals:
       Min         Q1        Med         Q3        Max
-2.1315915 -0.5513739  0.1227832  0.4588826  2.9834159

Number of Observations: 156
Number of Groups: 20
>
> residuals_medianp50 <- resid(mixed_model_medianp50)
> hist(residuals_medianp50, breaks = 20, main = "Histogram of Model Residu
als - Median P50",
+      xlab = "Residuals", col = "skyblue", border = "black")
> print(shapiro.test(residuals_medianp50))

	Shapiro-Wilk normality test
```

```
data:  residuals_medianp50
W = 0.98446, p-value = 0.0775


> #CI
> intervals(mixed_model_medianp50, which = "fixed", level = 0.95)
Approximate 95% confidence intervals

 Fixed effects:
                        lower         est.     upper
(Intercept)         8.0951481  8.68600000 9.2768519
Timepoint2         -0.5231347  0.22050000 0.9641347
Timepoint3         -0.6978013  0.04583333 0.7894680
Timepoint4         -1.1702785 -0.41602878 0.3382210
Group1             -0.3227180  0.42091667 1.1645513
Timepoint2:Group1 -1.4330749 -0.38141667 0.6702416
Timepoint3:Group1 -1.6786252 -0.61936991 0.4398853
Timepoint4:Group1 -1.2951535 -0.22001108 0.8551314
```

PBR 5-25

```
> mixed_model_prb525 <- lme(prb525 ~ Timepoint * Group, random = ~1 | ID1,
data = VO_combined_data)
> summary(mixed_model_prb525)
Linear mixed-effects model fit by REML
  Data: VO_combined_data
        AIC       BIC   logLik
  -40.27585 -10.30372 30.13792

Random effects:
 Formula: ~1 | ID1
        (Intercept)  Residual
StdDev:   0.0234169 0.1807351

Fixed effects:  prb525 ~ Timepoint * Group
                        Value  Std.Error  DF  t-value p-value
(Intercept)         2.4842500 0.04075140 129 60.96109  0.0000
Timepoint2          0.0686667 0.05715346 129  1.20144  0.2318
Timepoint3          0.1504167 0.05715346 129  2.63180  0.0095
Timepoint4         -0.0073353 0.05791175 129 -0.12666  0.8994
Group1              0.0950000 0.05715346 129  1.66219  0.0989
Timepoint2:Group1 -0.0300833 0.08082720 129 -0.37219  0.7104
Timepoint3:Group1 -0.0920411 0.08136528 129 -1.13121  0.2601
Timepoint4:Group1 -0.1171447 0.08249122 129 -1.42009  0.1580
 Correlation:
                  (Intr) Tmpnt2 Tmpnt3 Tmpnt4 Group1 Tm2:G1 Tm3:G1
Timepoint2        -0.701
Timepoint3        -0.701  0.500
Timepoint4        -0.692  0.493  0.493
Group1            -0.701  0.500  0.500  0.493
Timepoint2:Group1  0.496 -0.707 -0.354 -0.349 -0.707
Timepoint3:Group1  0.493 -0.351 -0.702 -0.347 -0.702  0.497
Timepoint4:Group1  0.486 -0.346 -0.346 -0.702 -0.693  0.490  0.487

Standardized Within-Group Residuals:
        Min          Q1         Med          Q3         Max
-3.57752391 -0.47902690  0.05507735  0.57512708  2.91549369

Number of Observations: 156
Number of Groups: 20
>
> residuals_prb525 <- resid(mixed_model_prb525)
> hist(residuals_prb525, breaks = 20, main = "Histogram of Model Residuals
- PBR 5-25",
+      xlab = "Residuals", col = "skyblue", border = "black")
> print(shapiro.test(residuals_prb525))
```

```
	Shapiro-Wilk normality test

data:  residuals_prb525
W = 0.96762, p-value = 0.001002


> #CI
> intervals(mixed_model_prb525, which = "fixed", level = 0.95)
Approximate 95% confidence intervals

Fixed effects:
                         lower          est.      upper
(Intercept)         2.40362235  2.484250000 2.56487765
Timepoint2         -0.04441286  0.068666667 0.18174619
Timepoint3          0.03733714  0.150416667 0.26349619
Timepoint4         -0.12191514 -0.007335325 0.10724449
Group1             -0.01807953  0.095000000 0.20807953
Timepoint2:Group1 -0.19000194 -0.030083333 0.12983527
Timepoint3:Group1 -0.25302432 -0.092041121 0.06894208
Timepoint4:Group1 -0.28035559 -0.117144679 0.04606623
```

PBR 5-9

```
> mixed_model_prb59 <- lme(prb59 ~ Timepoint * Group, random = ~1 | ID1, d
ata = VO_combined_data)
> summary(mixed_model_prb59)
Linear mixed-effects model fit by REML
  Data: VO_combined_data
        AIC       BIC   logLik
  -171.4534 -141.4813 95.72671

Random effects:
 Formula: ~1 | ID1
        (Intercept)  Residual
StdDev:   0.0189048 0.1155377

Fixed effects:  prb59 ~ Timepoint * Group
                       Value  Std.Error  DF  t-value p-value
(Intercept)        1.3361667 0.02617858 129 51.04046  0.0000
Timepoint2         0.0463333 0.03653624 129  1.26815  0.2070
Timepoint3         0.0886667 0.03653624 129  2.42681  0.0166
Timepoint4         0.0211819 0.03702456 129  0.57210  0.5682
Group1             0.0682500 0.03653624 129  1.86801  0.0640
Timepoint2:Group1 -0.0702500 0.05167004 129 -1.35959  0.1763
Timepoint3:Group1 -0.1156205 0.05201666 129 -2.22276  0.0280
Timepoint4:Group1 -0.1377854 0.05274228 129 -2.61243  0.0101
 Correlation: 
                  (Intr) Tmpnt2 Tmpnt3 Tmpnt4 Group1 Tm2:G1 Tm3:G1
Timepoint2        -0.698                                          
Timepoint3        -0.698  0.500                                   
Timepoint4        -0.689  0.493  0.493                            
Group1            -0.698  0.500  0.500  0.493                     
Timepoint2:Group1  0.493 -0.707 -0.354 -0.349 -0.707              
Timepoint3:Group1  0.490 -0.351 -0.702 -0.347 -0.702  0.497       
Timepoint4:Group1  0.483 -0.346 -0.346 -0.702 -0.693  0.490  0.487

Standardized Within-Group Residuals:
        Min          Q1         Med          Q3         Max 
-3.04050806 -0.51066263  0.05730926  0.55800425  3.12389782 

Number of Observations: 156
Number of Groups: 20 
> 
> residuals_prb59 <- resid(mixed_model_prb59)
> hist(residuals_prb59, breaks = 20, main = "Histogram of Model Residuals
- PBR 5-9",
+      xlab = "Residuals", col = "skyblue", border = "black")
```

```
> print(shapiro.test(residuals_prb59))

        Shapiro-Wilk normality test

data:  residuals_prb59
W = 0.9743, p-value = 0.005159

> #CI
> intervals(mixed_model_prb59, which = "fixed", level = 0.95)
Approximate 95% confidence intervals

 Fixed effects:
                         lower        est.       upper
(Intercept)        1.284371710  1.33616667  1.38796162
Timepoint2        -0.025954509  0.04633333  0.11862118
Timepoint3         0.016378824  0.08866667  0.16095451
Timepoint4        -0.052072065  0.02118193  0.09443592
Group1            -0.004037843  0.06825000  0.14053784
Timepoint2:Group1 -0.172480448 -0.07025000  0.03198045
Timepoint3:Group1 -0.218536754 -0.11562052 -0.01270429
Timepoint4:Group1 -0.242137263 -0.13778537 -0.03343348
```

PBR 10-19

```
> mixed_model_prb1019 <- lme(prb1019 ~ Timepoint * Group, random = ~1 | ID
1, data = VO_combined_data)
> summary(mixed_model_prb1019)
Linear mixed-effects model fit by REML
  Data: VO_combined_data
        AIC      BIC   logLik
  -4.972156 24.99997 12.48608

Random effects:
 Formula: ~1 | ID1
        (Intercept)  Residual
StdDev: 0.004590146 0.2051904

Fixed effects:  prb1019 ~ Timepoint * Group
                       Value  Std.Error  DF  t-value p-value
(Intercept)        2.8258333 0.04589345 129 61.57378  0.0000
Timepoint2         0.0968333 0.06488691 129  1.49234  0.1381
Timepoint3         0.1750000 0.06488691 129  2.69700  0.0079
Timepoint4         0.0001329 0.06573556 129  0.00202  0.9984
Group1             0.1080000 0.06488691 129  1.66443  0.0985
Timepoint2:Group1 -0.0384167 0.09176394 129 -0.41865  0.6762
Timepoint3:Group1 -0.1083331 0.09236598 129 -1.17287  0.2430
Timepoint4:Group1 -0.1408503 0.09362430 129 -1.50442  0.1349
 Correlation:
                  (Intr) Tmpnt2 Tmpnt3 Tmpnt4 Group1 Tm2:G1 Tm3:G1
Timepoint2        -0.707
Timepoint3        -0.707  0.500
Timepoint4        -0.698  0.494  0.494
Group1            -0.707  0.500  0.500  0.494
Timepoint2:Group1  0.500 -0.707 -0.354 -0.349 -0.707
Timepoint3:Group1  0.497 -0.351 -0.702 -0.347 -0.702  0.497
Timepoint4:Group1  0.490 -0.347 -0.347 -0.702 -0.693  0.490  0.487

Standardized Within-Group Residuals:
        Min          Q1         Med          Q3         Max
-3.21927296 -0.56918640  0.06396134  0.61467095  2.61126699

Number of Observations: 156
Number of Groups: 20
>
> residuals_prb1019 <- resid(mixed_model_prb1019)
> hist(residuals_prb1019, breaks = 20, main = "Histogram of Model Residual
s - PBR 10-19",
+      xlab = "Residuals", col = "skyblue", border = "black")
> print(shapiro.test(residuals_prb1019))
```

```
        Shapiro-Wilk normality test

data:  residuals_prb1019
W = 0.97861, p-value = 0.01586


> #CI
> intervals(mixed_model_prb1019, which = "fixed", level = 0.95)
Approximate 95% confidence intervals

 Fixed effects:
                        lower          est.      upper
(Intercept)        2.73503202  2.8258333333 2.91663465
Timepoint2        -0.03154700  0.0968333333 0.22521367
Timepoint3         0.04661966  0.1750000000 0.30338034
Timepoint4        -0.12992650  0.0001329166 0.13019233
Group1            -0.02038034  0.1080000000 0.23638034
Timepoint2:Group1 -0.21997388 -0.0384166667 0.14314055
Timepoint3:Group1 -0.29108141 -0.1083330506 0.07441531
Timepoint4:Group1 -0.32608831 -0.1408503430 0.04438763
```

PBR 20-25

```
> mixed_model_prb2025 <- lme(prb2025 ~ Timepoint * Group, random = ~1 | ID
1, data = VO_combined_data)
> summary(mixed_model_prb2025)
Linear mixed-effects model fit by REML
  Data: VO_combined_data
       AIC      BIC    logLik
  159.4672 189.4393 -69.7336

Random effects:
 Formula: ~1 | ID1
        (Intercept)  Residual
StdDev:  0.06590715 0.3522188

Fixed effects:  prb2025 ~ Timepoint * Group
                       Value  Std.Error  DF  t-value p-value
(Intercept)        2.9173333 0.08012548 129 36.40956  0.0000
Timepoint2         0.0651667 0.11138138 129  0.58508  0.5595
Timepoint3         0.2028333 0.11138138 129  1.82107  0.0709
Timepoint4         0.0077944 0.11287809 129  0.06905  0.9451
Group1             0.0663333 0.11138138 129  0.59555  0.5525
Timepoint2:Group1 -0.0401667 0.15751705 129 -0.25500  0.7991
Timepoint3:Group1 -0.0224101 0.15857975 129 -0.14132  0.8878
Timepoint4:Group1 -0.0157326 0.16080504 129 -0.09784  0.9222
 Correlation:
                  (Intr) Tmpnt2 Tmpnt3 Tmpnt4 Group1 Tm2:G1 Tm3:G1
Timepoint2        -0.695
Timepoint3        -0.695  0.500
Timepoint4        -0.686  0.493  0.493
Group1            -0.695  0.500  0.500  0.493
Timepoint2:Group1  0.491 -0.707 -0.354 -0.349 -0.707
Timepoint3:Group1  0.488 -0.351 -0.702 -0.347 -0.702  0.497
Timepoint4:Group1  0.481 -0.346 -0.346 -0.702 -0.693  0.490  0.487

Standardized Within-Group Residuals:
        Min          Q1         Med          Q3         Max
-2.69017494 -0.52257745  0.09269972  0.60262678  2.70291389

Number of Observations: 156
Number of Groups: 20
>
> residuals_prb2025 <- resid(mixed_model_prb2025)
> hist(residuals_prb2025, breaks = 20, main = "Histogram of Model Residual
s - PBR 20-25",
+      xlab = "Residuals", col = "skyblue", border = "black")
> print(shapiro.test(residuals_prb2025))
```

```
        Shapiro-Wilk normality test

data:  residuals_prb2025
W = 0.9846, p-value = 0.08047

> #CI
> intervals(mixed_model_prb2025, which = "fixed", level = 0.95)
Approximate 95% confidence intervals

 Fixed effects:
                        lower         est.     upper
(Intercept)        2.75880310  2.917333333 3.0758636
Timepoint2        -0.15520411  0.065166667 0.2855374
Timepoint3        -0.01753745  0.202833333 0.4232041
Timepoint4        -0.21553764  0.007794417 0.2311265
Group1            -0.15403745  0.066333333 0.2867041
Timepoint2:Group1 -0.35181801 -0.040166667 0.2714847
Timepoint3:Group1 -0.33616399 -0.022410074 0.2913438
Timepoint4:Group1 -0.33388930 -0.015732581 0.3024241
```

Sensitivity analysis for PBR vs group and timepoint + PCV

```
> model_prb525_original <- lme(
+   fixed = prb525 ~ Group * Timepoint,
+   random = ~1 | ID1,
+   data = Glycocheck_volume_overload_cats_dataset,
+   na.action = na.omit,
+   method = "REML"
+ )
>
> summary(model_prb525_original)
Linear mixed-effects model fit by REML
  Data: Glycocheck_volume_overload_cats_dataset
        AIC       BIC   logLik
  -40.27585 -10.30372 30.13792

Random effects:
 Formula: ~1 | ID1
        (Intercept)  Residual
StdDev:   0.0234169 0.1807351

Fixed effects:  prb525 ~ Group * Timepoint
                       Value  Std.Error  DF  t-value p-value
(Intercept)        2.4842500 0.04075140 129 60.96109  0.0000
Group1             0.0950000 0.05715346 129  1.66219  0.0989
Timepoint2         0.0686667 0.05715346 129  1.20144  0.2318
Timepoint3         0.1504167 0.05715346 129  2.63180  0.0095
Timepoint4        -0.0073353 0.05791175 129 -0.12666  0.8994
Group1:Timepoint2 -0.0300833 0.08082720 129 -0.37219  0.7104
Group1:Timepoint3 -0.0920411 0.08136528 129 -1.13121  0.2601
Group1:Timepoint4 -0.1171447 0.08249122 129 -1.42009  0.1580
 Correlation:
                  (Intr) Group1 Tmpnt2 Tmpnt3 Tmpnt4 Gr1:T2 Gr1:T3
Group1            -0.701
Timepoint2        -0.701  0.500
Timepoint3        -0.701  0.500  0.500
Timepoint4        -0.692  0.493  0.493  0.493
Group1:Timepoint2  0.496 -0.707 -0.707 -0.354 -0.349
Group1:Timepoint3  0.493 -0.702 -0.351 -0.702 -0.347  0.497
```

```
Group1:Timepoint4  0.486 -0.693 -0.346 -0.346 -0.702  0.490  0.487

Standardized Within-Group Residuals:
        Min          Q1         Med          Q3         Max 
-3.57752391 -0.47902690  0.05507735  0.57512708  2.91549369 

Number of Observations: 156
Number of Groups: 20 
> model_prb525_PCV <- lme(
+   fixed = prb525 ~ Group * Timepoint + PCV,
+   random = ~1 | ID1,
+   data = Glycocheck_volume_overload_cats_dataset,
+   na.action = na.omit,
+   method = "REML"
+ )
> 
> summary(model_prb525_PCV)
Linear mixed-effects model fit by REML
  Data: Glycocheck_volume_overload_cats_dataset 
        AIC       BIC   logLik
  -36.62822 -3.733457 29.31411

Random effects:
 Formula: ~1 | ID1
        (Intercept)  Residual
StdDev:  0.01383807 0.1779303

Fixed effects:  prb525 ~ Group * Timepoint + PCV 
                       Value  Std.Error  DF   t-value p-value
(Intercept)        2.9298310 0.17003620 128 17.230631  0.0000
Group1             0.1004505 0.05630281 128  1.784112  0.0768
Timepoint2         0.0230185 0.05875923 128  0.391742  0.6959
Timepoint3         0.1769880 0.05712329 128  3.098351  0.0024
Timepoint4         0.0224480 0.05807777 128  0.386516  0.6998
PCV               -0.0136263 0.00505465 128 -2.695803  0.0080
Group1:Timepoint2 -0.0873139 0.08235611 128 -1.060200  0.2911
Group1:Timepoint3 -0.1287994 0.08127394 128 -1.584757  0.1155
Group1:Timepoint4 -0.1311409 0.08137357 128 -1.611591  0.1095
 Correlation: 
                  (Intr) Group1 Tmpnt2 Tmpnt3 Tmpnt4 PCV    Gr1:T2 Gr1:T3
Group1            -0.130                                                 
Timepoint2        -0.439  0.468                                          
Timepoint3         0.005  0.498  0.422                                   
Timepoint4         0.026  0.491  0.409  0.510                            
PCV               -0.972 -0.036  0.288 -0.173 -0.191                     
Group1:Timepoint2 -0.138 -0.692 -0.580 -0.381 -0.380  0.258              
Group1:Timepoint3 -0.050 -0.698 -0.283 -0.711 -0.368  0.170  0.517       
Group1:Timepoint4  0.050 -0.693 -0.312 -0.352 -0.700  0.066  0.489  0.490

Standardized Within-Group Residuals:
        Min          Q1         Med          Q3         Max 
-3.33081701 -0.51413754  0.09787178  0.57860817  2.99012259 

Number of Observations: 156
Number of Groups: 20 
> intervals(model_prb525_PCV, which = "fixed")
Approximate 95% confidence intervals
```

```
 Fixed effects:
                          lower         est.        upper
(Intercept)          2.59338534  2.92983101  3.266276681
Group1              -0.01095419  0.10045053  0.211855258
Timepoint2          -0.09324672  0.02301846  0.139283634
Timepoint3           0.06395983  0.17698801  0.290016197
Timepoint4          -0.09246881  0.02244798  0.137364758
PCV                 -0.02362781 -0.01362633 -0.003624848
Group1:Timepoint2 -0.25026956 -0.08731392  0.075641714
Group1:Timepoint3 -0.28961376 -0.12879940  0.032014969
Group1:Timepoint4 -0.29215244 -0.13114092  0.029870592
> model_prb59_original <- lme(
+   fixed = prb59 ~ Group * Timepoint,
+   random = ~1 | ID1,
+   data = Glycocheck_volume_overload_cats_dataset,
+   na.action = na.omit,
+   method = "REML"
+ )
>
> summary(model_prb59_original)
Linear mixed-effects model fit by REML
  Data: Glycocheck_volume_overload_cats_dataset
        AIC       BIC   logLik
  -171.4534 -141.4813 95.72671

Random effects:
 Formula: ~1 | ID1
        (Intercept)  Residual
StdDev:   0.0189048 0.1155377

Fixed effects:  prb59 ~ Group * Timepoint
                       Value  Std.Error  DF  t-value p-value
(Intercept)          1.3361667 0.02617858 129 51.04046  0.0000
Group1               0.0682500 0.03653624 129  1.86801  0.0640
Timepoint2           0.0463333 0.03653624 129  1.26815  0.2070
Timepoint3           0.0886667 0.03653624 129  2.42681  0.0166
Timepoint4           0.0211819 0.03702456 129  0.57210  0.5682
Group1:Timepoint2 -0.0702500 0.05167004 129 -1.35959  0.1763
Group1:Timepoint3 -0.1156205 0.05201666 129 -2.22276  0.0280
Group1:Timepoint4 -0.1377854 0.05274228 129 -2.61243  0.0101
 Correlation:
                  (Intr) Group1 Tmpnt2 Tmpnt3 Tmpnt4 Gr1:T2 Gr1:T3
Group1            -0.698
Timepoint2        -0.698  0.500
Timepoint3        -0.698  0.500  0.500
Timepoint4        -0.689  0.493  0.493  0.493
Group1:Timepoint2  0.493 -0.707 -0.707 -0.354 -0.349
Group1:Timepoint3  0.490 -0.702 -0.351 -0.702 -0.347  0.497
Group1:Timepoint4  0.483 -0.693 -0.346 -0.346 -0.702  0.490  0.487

Standardized Within-Group Residuals:
        Min          Q1         Med          Q3         Max
-3.04050806 -0.51066263  0.05730926  0.55800425  3.12389782

Number of Observations: 156
Number of Groups: 20
> model_prb59_PCV <- lme(
+   fixed = prb59 ~ Group * Timepoint + PCV,
```

```
+   random = ~1 | ID1,
+   data = Glycocheck_volume_overload_cats_dataset,
+   na.action = na.omit,
+   method = "REML"
+ )
>
> summary(model_prb59_PCV)
Linear mixed-effects model fit by REML
  Data: Glycocheck_volume_overload_cats_dataset
        AIC       BIC   logLik
  -161.3585 -128.4638 91.67926

Random effects:
 Formula: ~1 | ID1
        (Intercept)  Residual
StdDev:  0.01828673 0.1154391

Fixed effects:  prb59 ~ Group * Timepoint + PCV
                       Value  Std.Error  DF   t-value p-value
(Intercept)        1.4689363 0.11290620 128 13.010236  0.0000
Group1             0.0698741 0.03652976 128  1.912799  0.0580
Timepoint2         0.0327315 0.03820002 128  0.856846  0.3931
Timepoint3         0.0965841 0.03708803 128  2.604186  0.0103
Timepoint4         0.0300888 0.03772502 128  0.797583  0.4266
PCV               -0.0040602 0.00335902 128 -1.208757  0.2290
Group1:Timepoint2 -0.0873030 0.05351886 128 -1.631256  0.1053
Group1:Timepoint3 -0.1265691 0.05275857 128 -2.399025  0.0179
Group1:Timepoint4 -0.1420085 0.05281297 128 -2.688895  0.0081
 Correlation:
                  (Intr) Group1 Tmpnt2 Tmpnt3 Tmpnt4 PCV    Gr1:T2 Gr1:T3
Group1            -0.126
Timepoint2        -0.441  0.467
Timepoint3         0.013  0.498  0.418
Timepoint4         0.034  0.491  0.405  0.511
PCV               -0.973 -0.037  0.295 -0.177 -0.196
Group1:Timepoint2 -0.146 -0.691 -0.574 -0.382 -0.382  0.264
Group1:Timepoint3 -0.056 -0.698 -0.280 -0.711 -0.369  0.172  0.517
Group1:Timepoint4  0.047 -0.693 -0.311 -0.352 -0.700  0.067  0.489  0.490

Standardized Within-Group Residuals:
        Min          Q1         Med          Q3         Max
-2.97658860 -0.52680523  0.05359696  0.59412439  3.12728992

Number of Observations: 156
Number of Groups: 20
> model_prb1019_original <- lme(
+   fixed = prb1019 ~ Group * Timepoint,
+   random = ~1 | ID1,
+   data = Glycocheck_volume_overload_cats_dataset,
+   na.action = na.omit,
+   method = "REML"
+ )
>
> summary(model_prb1019_original)
Linear mixed-effects model fit by REML
  Data: Glycocheck_volume_overload_cats_dataset
        AIC      BIC   logLik
  -4.972156 24.99997 12.48608
```

```
Random effects:
 Formula: ~1 | ID1
        (Intercept)  Residual
StdDev: 0.004590147 0.2051904

Fixed effects:  prb1019 ~ Group * Timepoint
                       Value  Std.Error  DF  t-value p-value
(Intercept)        2.8258333 0.04589345 129 61.57378  0.0000
Group1             0.1080000 0.06488691 129  1.66443  0.0985
Timepoint2         0.0968333 0.06488691 129  1.49234  0.1381
Timepoint3         0.1750000 0.06488691 129  2.69700  0.0079
Timepoint4         0.0001329 0.06573556 129  0.00202  0.9984
Group1:Timepoint2 -0.0384167 0.09176394 129 -0.41865  0.6762
Group1:Timepoint3 -0.1083331 0.09236598 129 -1.17287  0.2430
Group1:Timepoint4 -0.1408503 0.09362430 129 -1.50442  0.1349
 Correlation:
                  (Intr) Group1 Tmpnt2 Tmpnt3 Tmpnt4 Gr1:T2 Gr1:T3
Group1            -0.707
Timepoint2        -0.707  0.500
Timepoint3        -0.707  0.500  0.500
Timepoint4        -0.698  0.494  0.494  0.494
Group1:Timepoint2  0.500 -0.707 -0.707 -0.354 -0.349
Group1:Timepoint3  0.497 -0.702 -0.351 -0.702 -0.347  0.497
Group1:Timepoint4  0.490 -0.693 -0.347 -0.347 -0.702  0.490  0.487

Standardized Within-Group Residuals:
        Min          Q1         Med          Q3         Max
-3.21927296 -0.56918640  0.06396134  0.61467094  2.61126699

Number of Observations: 156
Number of Groups: 20
> model_prb1019_PCV <- lme(
+   fixed = prb1019 ~ Group * Timepoint + PCV,
+   random = ~1 | ID1,
+   data = Glycocheck_volume_overload_cats_dataset,
+   na.action = na.omit,
+   method = "REML"
+ )
>
> summary(model_prb1019_PCV)
Linear mixed-effects model fit by REML
  Data: Glycocheck_volume_overload_cats_dataset
        AIC      BIC   logLik
  -1.757557 31.1372 11.87878

Random effects:
 Formula: ~1 | ID1
         (Intercept)  Residual
StdDev: 6.729742e-06 0.2009216

Fixed effects:  prb1019 ~ Group * Timepoint + PCV
                      Value  Std.Error  DF   t-value p-value
(Intercept)        3.330247 0.19039189 128 17.491538  0.0000
Group1             0.114170 0.06357728 128  1.795770  0.0749
Timepoint2         0.045158 0.06630391 128  0.681075  0.4971
Timepoint3         0.205080 0.06448780 128  3.180132  0.0018
Timepoint4         0.033986 0.06555444 128  0.518447  0.6050
```

```
PCV                 -0.015425 0.00565795 128 -2.726337  0.0073
Group1:Timepoint2 -0.103204 0.09294406 128 -1.110385  0.2689
Group1:Timepoint3 -0.150484 0.09175759 128 -1.640012  0.1035
Group1:Timepoint4 -0.157361 0.09187618 128 -1.712749  0.0892
 Correlation: 
                  (Intr) Group1 Tmpnt2 Tmpnt3 Tmpnt4 PCV    Gr1:T2 Gr1:T3
Group1            -0.132                                                 
Timepoint2        -0.438  0.469                                          
Timepoint3         0.002  0.498  0.423                                   
Timepoint4         0.022  0.491  0.410  0.510                            
PCV               -0.972 -0.036  0.286 -0.171 -0.189                     
Group1:Timepoint2 -0.134 -0.692 -0.582 -0.381 -0.380  0.256              
Group1:Timepoint3 -0.048 -0.698 -0.284 -0.711 -0.368  0.169  0.516       
Group1:Timepoint4  0.051 -0.693 -0.312 -0.352 -0.700  0.066  0.490  0.490

Standardized Within-Group Residuals:
        Min          Q1         Med          Q3         Max 
-2.98793451 -0.62995692  0.07197374  0.62986376  2.66085931 

Number of Observations: 156
Number of Groups: 20 
> model_prb2025_original <- lme(
+   fixed = prb2025 ~ Group * Timepoint,
+   random = ~1 | ID1,
+   data = Glycocheck_volume_overload_cats_dataset,
+   na.action = na.omit,
+   method = "REML"
+ )
> 
> summary(model_prb2025_original)
Linear mixed-effects model fit by REML
  Data: Glycocheck_volume_overload_cats_dataset 
       AIC      BIC    logLik
  159.4672 189.4393 -69.7336

Random effects:
 Formula: ~1 | ID1
        (Intercept)  Residual
StdDev:  0.06590715 0.3522188

Fixed effects:  prb2025 ~ Group * Timepoint 
                       Value  Std.Error  DF  t-value p-value
(Intercept)        2.9173333 0.08012548 129 36.40956  0.0000
Group1             0.0663333 0.11138138 129  0.59555  0.5525
Timepoint2         0.0651667 0.11138138 129  0.58508  0.5595
Timepoint3         0.2028333 0.11138138 129  1.82107  0.0709
Timepoint4         0.0077944 0.11287809 129  0.06905  0.9451
Group1:Timepoint2 -0.0401667 0.15751705 129 -0.25500  0.7991
Group1:Timepoint3 -0.0224101 0.15857975 129 -0.14132  0.8878
Group1:Timepoint4 -0.0157326 0.16080504 129 -0.09784  0.9222
 Correlation: 
                  (Intr) Group1 Tmpnt2 Tmpnt3 Tmpnt4 Gr1:T2 Gr1:T3
Group1            -0.695                                          
Timepoint2        -0.695  0.500                                   
Timepoint3        -0.695  0.500  0.500                            
Timepoint4        -0.686  0.493  0.493  0.493                     
Group1:Timepoint2  0.491 -0.707 -0.707 -0.354 -0.349              
Group1:Timepoint3  0.488 -0.702 -0.351 -0.702 -0.347  0.497       
```

```
Group1:Timepoint4  0.481 -0.693 -0.346 -0.346 -0.702  0.490  0.487

Standardized Within-Group Residuals:
        Min          Q1         Med          Q3         Max 
-2.69017494 -0.52257745  0.09269972  0.60262678  2.70291389 

Number of Observations: 156
Number of Groups: 20 
> model_prb2025_PCV <- lme(
+   fixed = prb2025 ~ Group * Timepoint + PCV,
+   random = ~1 | ID1,
+   data = Glycocheck_volume_overload_cats_dataset,
+   na.action = na.omit,
+   method = "REML"
+ )
> 
> summary(model_prb2025_PCV)
Linear mixed-effects model fit by REML
  Data: Glycocheck_volume_overload_cats_dataset 
     AIC      BIC    logLik
  161.01 193.9048 -69.50502

Random effects:
 Formula: ~1 | ID1
        (Intercept)  Residual
StdDev:  0.07318152 0.3428502

Fixed effects:  prb2025 ~ Group * Timepoint + PCV 
                      Value Std.Error  DF   t-value p-value
(Intercept)        3.858703 0.3422427 128 11.274755  0.0000
Group1             0.077849 0.1084953 128  0.717529  0.4744
Timepoint2        -0.031273 0.1136637 128 -0.275139  0.7837
Timepoint3         0.258970 0.1102238 128  2.349492  0.0203
Timepoint4         0.071514 0.1121625 128  0.637593  0.5249
PCV               -0.028788 0.0101879 128 -2.825713  0.0055
Group1:Timepoint2 -0.161077 0.1591859 128 -1.011877  0.3135
Group1:Timepoint3 -0.099980 0.1567681 128 -0.637755  0.5248
Group1:Timepoint4 -0.045934 0.1569004 128 -0.292758  0.7702
 Correlation: 
                  (Intr) Group1 Tmpnt2 Tmpnt3 Tmpnt4 PCV    Gr1:T2 Gr1:T3
Group1            -0.122                                                 
Timepoint2        -0.443  0.465                                          
Timepoint3         0.020  0.498  0.415                                   
Timepoint4         0.042  0.491  0.401  0.512                            
PCV               -0.973 -0.038  0.300 -0.180 -0.201                     
Group1:Timepoint2 -0.154 -0.691 -0.569 -0.383 -0.383  0.269              
Group1:Timepoint3 -0.060 -0.698 -0.278 -0.712 -0.369  0.174  0.518       
Group1:Timepoint4  0.044 -0.693 -0.309 -0.352 -0.700  0.067  0.489  0.490

Standardized Within-Group Residuals:
        Min          Q1         Med          Q3         Max 
-2.58055385 -0.55120364  0.09651569  0.56761118  2.94973677 

Number of Observations: 156
Number of Groups: 20 
```

2. Outliers excluded

Density – VVD

```
> cleaned_data <- VO_combined_data[-outlier_rows, ]
> cleaned_data$Group <- relevel(as.factor(cleaned_data$Group), ref = "2")
> cleaned_data$Timepoint <- as.factor(cleaned_data$Timepoint)
> mixed_model <- lme(density ~ Timepoint * Group, random = ~1 | ID1, data
= cleaned_data)

> summary(mixed_model)
Linear mixed-effects model fit by REML
  Data: cleaned_data
      AIC      BIC    logLik
  1793.93 1823.766 -886.9651

Random effects:
 Formula: ~1 | ID1
        (Intercept) Residual
StdDev:    23.57783 94.61211

Fixed effects:  density ~ Timepoint * Group
                    Value Std.Error  DF   t-value p-value
(Intercept)      483.7332  22.35945 127 21.634397  0.0000
Timepoint2       -34.9166  30.32692 127 -1.151339  0.2518
Timepoint3       -41.6999  30.32692 127 -1.375013  0.1715
Timepoint4       -21.5553  30.73125 127 -0.701414  0.4843
Group1           -30.7749  30.32692 127 -1.014772  0.3121
Timepoint2:Group1  0.1226  42.88745 127  0.002859  0.9977
Timepoint3:Group1 18.9708  42.88792 127  0.442335  0.6590
Timepoint4:Group1 13.5137  43.49121 127  0.310721  0.7565
 Correlation:
                  (Intr) Tmpnt2 Tmpnt3 Tmpnt4 Group1 Tm2:G1 Tm3:G1
Timepoint2        -0.696
Timepoint3        -0.696  0.513
Timepoint4        -0.687  0.507  0.507
Group1            -0.696  0.513  0.513  0.507
Timepoint2:Group1  0.492 -0.707 -0.363 -0.358 -0.707
Timepoint3:Group1  0.492 -0.363 -0.707 -0.358 -0.707  0.500
Timepoint4:Group1  0.485 -0.358 -0.358 -0.707 -0.697  0.493  0.494

Standardized Within-Group Residuals:
        Min          Q1         Med          Q3         Max
-2.62362164 -0.56845325  0.03629202  0.65403600  2.36773561

Number of Observations: 154
Number of Groups: 20

> residuals_model <- resid(mixed_model)
>
> hist(residuals_model, breaks = 20, main = "Histogram of Model Residuals"
,
+      xlab = "Residuals", col = "skyblue", border = "black")
>
> shapiro_test <- shapiro.test(residuals_model)
> print(shapiro_test)

        Shapiro-Wilk normality test

data:  residuals_model
W = 0.99352, p-value = 0.7218

> #CI
> intervals(mixed_model_density, which = "fixed", level = 0.95)
Approximate 95% confidence intervals

 Fixed effects:
```

```
                         lower        est.      upper
(Intercept)          421.82233 466.933333 512.04433
Timepoint2           -81.15864 -18.116667  44.92531
Timepoint3           -87.94197 -24.900000  38.14197
Timepoint4           -68.12986  -4.246897  59.63606
Group1               -77.01697 -13.975000  49.06697
Timepoint2:Group1 -118.01314 -28.858333  60.29648
Timepoint3:Group1  -87.90650   1.845205  91.59691
Timepoint4:Group1  -94.88780  -3.886661  87.11448
```

% RBC filling

```
> outlier_rows <- c(9, 23, 36, 54, 113, 127)
> cleaned_data <- VO_combined_data[-outlier_rows, ]
> cleaned_data$Group <- relevel(as.factor(cleaned_data$Group), ref = "2")
> cleaned_data$Timepoint <- as.factor(cleaned_data$Timepoint)
> mixed_model <- lme(rbcfilling ~ Timepoint * Group, random = ~1 | ID1,
data = cleaned_data)
> summary(mixed_model)
Linear mixed-effects model fit by REML
  Data: cleaned_data
       AIC       BIC  logLik
  -422.734 -393.1757 221.367

Random effects:
 Formula: ~1 | ID1
        (Intercept)   Residual
StdDev:  0.00743106 0.04632137

Fixed effects:  rbcfilling ~ Timepoint * Group
                       Value  Std.Error  DF  t-value p-value
(Intercept)        0.6112066 0.01105579 123 55.28383  0.0000
Timepoint2        -0.0145066 0.01505832 123 -0.96336  0.3373
Timepoint3        -0.0443816 0.01524963 123 -2.91034  0.0043
Timepoint4         0.0040721 0.01524963 123  0.26703  0.7899
Group1            -0.0196197 0.01524963 123 -1.28657  0.2007
Timepoint2:Group1  0.0097197 0.02114517 123  0.45967  0.6466
Timepoint3:Group1  0.0451480 0.02157286 123  2.09282  0.0384
Timepoint4:Group1  0.0173114 0.02173865 123  0.79634  0.4274
 Correlation:
                  (Intr) Tmpnt2 Tmpnt3 Tmpnt4 Group1 Tm2:G1 Tm3:G1
Timepoint2        -0.718
Timepoint3        -0.709  0.520
Timepoint4        -0.709  0.520  0.514
Group1            -0.709  0.520  0.514  0.514
Timepoint2:Group1  0.511 -0.712 -0.371 -0.371 -0.721
Timepoint3:Group1  0.502 -0.368 -0.707 -0.364 -0.707  0.510
Timepoint4:Group1  0.498 -0.365 -0.361 -0.702 -0.702  0.506  0.497

Standardized Within-Group Residuals:
        Min          Q1         Med          Q3         Max
-2.20237621 -0.68930964 -0.02002747  0.60310662  2.52502933

Number of Observations: 150
Number of Groups: 20

> residuals_model <- resid(mixed_model)
> hist(residuals_model, breaks = 20, main = "Histogram of Model Residuals
RBC filling",
+      xlab = "Residuals", col = "skyblue", border = "black")

> shapiro_test <- shapiro.test(residuals_model)
> print(shapiro_test)

      Shapiro-Wilk normality test

data:  residuals_model
W = 0.99218, p-value = 0.5843
```

```
> #CI
> intervals(mixed_model_rbcfilling, which = "fixed", level = 0.95)
Approximate 95% confidence intervals

 Fixed effects:
                        lower        est.        upper
(Intercept)        0.60118438  0.62540833  0.649632287
Timepoint2        -0.06296618 -0.02870833  0.005549510
Timepoint3        -0.08389951 -0.04964167 -0.015383824
Timepoint4        -0.04478068 -0.01007500  0.024630677
Group1            -0.05935784 -0.02510000  0.009157843
Timepoint2:Group1 -0.03324791  0.01520000  0.063647906
Timepoint3:Group1 -0.01439718  0.03436842  0.083134022
Timepoint4:Group1 -0.01899625  0.03043333  0.079862918
```

Median P50

```
> cleaned_data <- VO_combined_data[-outlier_rows, ]
>
> cleaned_data$Group <- relevel(as.factor(cleaned_data$Group), ref = "2")
>
> cleaned_data$Timepoint <- as.factor(cleaned_data$Timepoint)
>
> mixed_model <- lme(medianp50 ~ Timepoint * Group, random = ~1 | ID1, dat
a = cleaned_data)
>
> summary(mixed_model)

Linear mixed-effects model fit by REML
  Data: cleaned_data
       AIC      BIC    logLik
  512.4334 542.2695 -246.2167

Random effects:
 Formula: ~1 | ID1
        (Intercept) Residual
StdDev:   0.5489017 1.126412

Fixed effects:  medianp50 ~ Timepoint * Group
                      Value Std.Error  DF   t-value p-value
(Intercept)        8.686000 0.2801873 127 31.000695  0.0000
Timepoint2         0.012855 0.3613616 127  0.035574  0.9717
Timepoint3         0.045833 0.3562029 127  0.128672  0.8978
Timepoint4        -0.415156 0.3612724 127 -1.149149  0.2527
Group1             0.245390 0.3612724 127  0.679239  0.4982
Timepoint2:Group1  0.001754 0.5109389 127  0.003434  0.9973
Timepoint3:Group1 -0.464860 0.5110199 127 -0.909671  0.3647
Timepoint4:Group1 -0.066179 0.5185591 127 -0.127621  0.8987
 Correlation:
                  (Intr) Tmpnt2 Tmpnt3 Tmpnt4 Group1 Tm2:G1 Tm3:G1
Timepoint2        -0.627
Timepoint3        -0.636  0.493
Timepoint4        -0.627  0.486  0.493
Group1            -0.627  0.486  0.493  0.486
Timepoint2:Group1  0.443 -0.707 -0.349 -0.343 -0.707
Timepoint3:Group1  0.443 -0.341 -0.697 -0.344 -0.707  0.498
Timepoint4:Group1  0.437 -0.336 -0.343 -0.697 -0.697  0.491  0.494

Standardized Within-Group Residuals:
       Min         Q1        Med         Q3        Max
-2.2925009 -0.5807530  0.1936139  0.5692274  2.2519531

Number of Observations: 154
Number of Groups: 20
>
> residuals_model <- resid(mixed_model)
```

```
>
> hist(residuals_model, breaks = 20, main = "Histogram of Model Residuals
Median P50",
+      xlab = "Residuals", col = "skyblue", border = "black")
>
> # Perform the Shapiro-Wilk normality test
> shapiro_test <- shapiro.test(residuals_model)
> print(shapiro_test)

        Shapiro-Wilk normality test

data:  residuals_model
W = 0.98471, p-value = 0.08704

> #CI
> intervals(mixed_model_medianp50, which = "fixed", level = 0.95)
Approximate 95% confidence intervals

 Fixed effects:
                         lower         est.     upper
(Intercept)          8.0951481  8.68600000 9.2768519
Timepoint2          -0.5231347  0.22050000 0.9641347
Timepoint3          -0.6978013  0.04583333 0.7894680
Timepoint4          -1.1702785 -0.41602878 0.3382210
Group1              -0.3227180  0.42091667 1.1645513
Timepoint2:Group1 -1.4330749 -0.38141667 0.6702416
Timepoint3:Group1 -1.6786252 -0.61936991 0.4398853
Timepoint4:Group1 -1.2951535 -0.22001108 0.8551314
```

PBR 5 – 25

```
> outlier_rows <- c(9, 47, 72, 82, 111, 127)
> cleaned_data <- VO_combined_data[-outlier_rows, ]
>
> cleaned_data$Group <- relevel(as.factor(cleaned_data$Group), ref = "2")
>
> cleaned_data$Timepoint <- as.factor(cleaned_data$Timepoint)
>
> mixed_model <- lme(prb525 ~ Timepoint * Group, random = ~1 | ID1, data =
cleaned_data)
>
> summary(mixed_model)

Linear mixed-effects model fit by REML
  Data: cleaned_data
        AIC       BIC   logLik
  -92.08223 -62.52396 56.04111

Random effects:
 Formula: ~1 | ID1
        (Intercept)  Residual
StdDev:  0.04429173 0.1449313

Fixed effects:  prb525 ~ Timepoint * Group
                         Value  Std.Error  DF  t-value p-value
(Intercept)          2.5343619 0.03566565 123 71.05890  0.0000
Timepoint2           0.0450551 0.04778467 123  0.94288  0.3476
Timepoint3           0.1003047 0.04716148 123  2.12684  0.0354
Timepoint4          -0.0298586 0.04846786 123 -0.61605  0.5390
Group1               0.0529769 0.04846786 123  1.09303  0.2765
Timepoint2:Group1 -0.0145606 0.06714234 123 -0.21686  0.8287
Timepoint3:Group1 -0.0507174 0.06720350 123 -0.75468  0.4519
Timepoint4:Group1 -0.1036491 0.06860505 123 -1.51081  0.1334
 Correlation:
                  (Intr) Tmpnt2 Tmpnt3 Tmpnt4 Group1 Tm2:G1 Tm3:G1
Timepoint2        -0.689
Timepoint3        -0.698  0.521
Timepoint4        -0.680  0.507  0.514
```

```
Group1              -0.680  0.507  0.514  0.500
Timepoint2:Group1  0.491 -0.712 -0.371 -0.361 -0.722
Timepoint3:Group1  0.492 -0.367 -0.703 -0.362 -0.722  0.522
Timepoint4:Group1  0.482 -0.360 -0.364 -0.708 -0.708  0.511  0.512

Standardized Within-Group Residuals:
        Min          Q1         Med          Q3         Max
-2.69356837 -0.62498159  0.02852801  0.60741887  2.10199092

Number of Observations: 150
Number of Groups: 20
>
> residuals_model <- resid(mixed_model)
>
> hist(residuals_model, breaks = 20, main = "Histogram of Model Residuals
PBR 5-25",
+      xlab = "Residuals", col = "skyblue", border = "black")
>
> shapiro_test <- shapiro.test(residuals_model)
> print(shapiro_test)

        Shapiro-Wilk normality test

data:  residuals_model
W = 0.99091, p-value = 0.4496


> #CI
> intervals(mixed_model_prb525, which = "fixed", level = 0.95)
Approximate 95% confidence intervals

 Fixed effects:
                        lower         est.      upper
(Intercept)        2.40362235  2.484250000 2.56487765
Timepoint2        -0.04441286  0.068666667 0.18174619
Timepoint3         0.03733714  0.150416667 0.26349619
Timepoint4        -0.12191514 -0.007335325 0.10724449
Group1            -0.01807953  0.095000000 0.20807953
Timepoint2:Group1 -0.19000194 -0.030083333 0.12983527
Timepoint3:Group1 -0.25302432 -0.092041121 0.06894208
Timepoint4:Group1 -0.28035559 -0.117144679 0.04606623
```

PBR 5-9

```
> outlier_rows <- c(25, 26, 58, 111, 112, 127)
>
> cleaned_data <- VO_combined_data[-outlier_rows, ]
>
> cleaned_data$Group <- relevel(as.factor(cleaned_data$Group), ref = "2")
>
> cleaned_data$Timepoint <- as.factor(cleaned_data$Timepoint)
>
> mixed_model <- lme(prb59 ~ Timepoint * Group, random = ~1 | ID1, data =
cleaned_data)
>
> summary(mixed_model)

Linear mixed-effects model fit by REML
  Data: cleaned_data
        AIC       BIC   logLik
  -204.4868 -174.9285 112.2434

Random effects:
 Formula: ~1 | ID1
        (Intercept)   Residual
StdDev:  0.01968149 0.09935057

Fixed effects:  prb59 ~ Timepoint * Group
```

```
                      Value  Std.Error  DF  t-value p-value
(Intercept)        1.3389694 0.02386479 123 56.10648  0.0000
Timepoint2         0.0388067 0.03269269 123  1.18701  0.2375
Timepoint3         0.0858640 0.03230614 123  2.65782  0.0089
Timepoint4         0.0186063 0.03272089 123  0.56864  0.5706
Group1             0.0464825 0.03272138 123  1.42055  0.1580
Timepoint2:Group1 -0.0278418 0.04591095 123 -0.60643  0.5453
Timepoint3:Group1 -0.0934016 0.04567677 123 -2.04484  0.0430
Timepoint4:Group1 -0.0980645 0.04667672 123 -2.10093  0.0377
 Correlation: 
                  (Intr) Tmpnt2 Tmpnt3 Tmpnt4 Group1 Tm2:G1 Tm3:G1
Timepoint2        -0.704                                          
Timepoint3        -0.714  0.520                                   
Timepoint4        -0.705  0.514  0.521                            
Group1            -0.705  0.514  0.521  0.514                     
Timepoint2:Group1  0.501 -0.712 -0.370 -0.366 -0.711              
Timepoint3:Group1  0.506 -0.368 -0.708 -0.369 -0.717  0.510       
Timepoint4:Group1  0.496 -0.361 -0.366 -0.702 -0.702  0.499  0.504

Standardized Within-Group Residuals:
        Min          Q1         Med          Q3         Max 
-3.63568850 -0.55419040  0.09411043  0.67114210  2.85268746 

Number of Observations: 150
Number of Groups: 20 
> 
> residuals_model <- resid(mixed_model)
> 
> hist(residuals_model, breaks = 20, main = "Histogram of Model Residuals PBR 5-9",
+      xlab = "Residuals", col = "skyblue", border = "black")
> 
> shapiro_test <- shapiro.test(residuals_model)
> print(shapiro_test)

	Shapiro-Wilk normality test

data:  residuals_model
W = 0.98722, p-value = 0.184

#CI
> intervals(mixed_model_prb59, which = "fixed", level = 0.95)
Approximate 95% confidence intervals

 Fixed effects:
                        lower        est.       upper
(Intercept)        1.284371710  1.33616667  1.38796162
Timepoint2        -0.025954509  0.04633333  0.11862118
Timepoint3         0.016378824  0.08866667  0.16095451
Timepoint4        -0.052072065  0.02118193  0.09443592
Group1            -0.004037843  0.06825000  0.14053784
Timepoint2:Group1 -0.172480448 -0.07025000  0.03198045
Timepoint3:Group1 -0.218536754 -0.11562052 -0.01270429
Timepoint4:Group1 -0.242137263 -0.13778537 -0.03343348
```

PBR 10-19

```
> outlier_rows <- c(9, 47, 82, 127)
> 
> cleaned_data <- VO_combined_data[-outlier_rows, ]
> 
> cleaned_data$Group <- relevel(as.factor(cleaned_data$Group), ref = "2")
> 
> cleaned_data$Timepoint <- as.factor(cleaned_data$Timepoint)
> 
```

```
> mixed_model <- lme(prb525 ~ Timepoint * Group, random = ~1 | ID1, data =
cleaned_data)
>
> summary(mixed_model)

Linear mixed-effects model fit by REML
  Data: cleaned_data
      AIC       BIC   logLik
  -74.227 -44.52887 47.1135

Random effects:
 Formula: ~1 | ID1
        (Intercept)  Residual
StdDev:  0.03193331 0.1579199

Fixed effects:  prb525 ~ Timepoint * Group
                      Value  Std.Error  DF  t-value p-value
(Intercept)         2.5353681 0.03796076 125 66.78918  0.0000
Timepoint2          0.0175485 0.05135141 125  0.34173  0.7331
Timepoint3          0.0992985 0.05135141 125  1.93371  0.0554
Timepoint4         -0.0313859 0.05273486 125 -0.59516  0.5528
Group1              0.0789034 0.05201130 125  1.51704  0.1318
Timepoint2:Group1 -0.0139867 0.07210441 125 -0.19398  0.8465
Timepoint3:Group1 -0.0760279 0.07260580 125 -1.04713  0.2971
Timepoint4:Group1 -0.1283429 0.07410394 125 -1.73193  0.0858
 Correlation:
                  (Intr) Tmpnt2 Tmpnt3 Tmpnt4 Group1 Tm2:G1 Tm3:G1
Timepoint2        -0.713
Timepoint3        -0.713  0.527
Timepoint4        -0.695  0.513  0.513
Group1            -0.704  0.521  0.521  0.507
Timepoint2:Group1  0.508 -0.712 -0.375 -0.366 -0.721
Timepoint3:Group1  0.505 -0.373 -0.708 -0.364 -0.717  0.517
Timepoint4:Group1  0.495 -0.366 -0.366 -0.712 -0.703  0.507  0.504

Standardized Within-Group Residuals:
        Min          Q1         Med          Q3         Max
-2.93093660 -0.57748996  0.07355337  0.57202212  3.06513513

Number of Observations: 152
Number of Groups: 20
>
> residuals_model <- resid(mixed_model)
>
> hist(residuals_model, breaks = 20, main = "Histogram of Model Residuals
PBR 10-19",
+      xlab = "Residuals", col = "skyblue", border = "black")
>
> shapiro_test <- shapiro.test(residuals_model)
> print(shapiro_test)

        Shapiro-Wilk normality test

data:  residuals_model
W = 0.99207, p-value = 0.5617

> #CI
> intervals(mixed_model_prb1019, which = "fixed", level = 0.95)
Approximate 95% confidence intervals

 Fixed effects:
                        lower          est.      upper
(Intercept)         2.73503202  2.8258333333 2.91663465
Timepoint2         -0.03154700  0.0968333333 0.22521367
Timepoint3          0.04661966  0.1750000000 0.30338034
Timepoint4         -0.12992650  0.0001329166 0.13019233
Group1             -0.02038034  0.1080000000 0.23638034
Timepoint2:Group1 -0.21997388 -0.0384166667 0.14314055
Timepoint3:Group1 -0.29108141 -0.1083330506 0.07441531
```

```
Timepoint4:Group1 -0.32608831 -0.1408503430 0.04438763
```

PBR 20-25

```
> outlier_rows <- c(2, 9, 18, 47, 80, 155)
>
> cleaned_data <- VO_combined_data[-outlier_rows, ]
>
> cleaned_data$Group <- relevel(as.factor(cleaned_data$Group), ref = "2")
>
> cleaned_data$Timepoint <- as.factor(cleaned_data$Timepoint)
>
> mixed_model <- lme(prb2025 ~ Timepoint * Group, random = ~1 | ID1, data
= cleaned_data)
>
> summary(mixed_model)

Linear mixed-effects model fit by REML
  Data: cleaned_data
      AIC      BIC    logLik
  119.681 149.2392 -49.84049

Random effects:
 Formula: ~1 | ID1
        (Intercept)  Residual
StdDev:  0.06244765 0.3109527

Fixed effects:  prb2025 ~ Timepoint * Group
                      Value  Std.Error  DF  t-value p-value
(Intercept)       2.9580091 0.07274631 123 40.66198  0.0000
Timepoint2        0.0147643 0.10240863 123  0.14417  0.8856
Timepoint3        0.1621576 0.09965750 123  1.62715  0.1063
Timepoint4       -0.0329484 0.10096998 123 -0.32632  0.7447
Group1            0.0770296 0.10096998 123  0.76290  0.4470
Timepoint2:Group1  0.0090123 0.14381399 123  0.06267  0.9501
Timepoint3:Group1 -0.0713652 0.14289312 123 -0.49943  0.6184
Timepoint4:Group1 -0.0269702 0.14381499 123 -0.18753  0.8516
 Correlation: 
                  (Intr) Tmpnt2 Tmpnt3 Tmpnt4 Group1 Tm2:G1 Tm3:G1
Timepoint2        -0.684                                          
Timepoint3        -0.703  0.500                                   
Timepoint4        -0.694  0.493  0.507                            
Group1            -0.694  0.493  0.507  0.500                     
Timepoint2:Group1  0.487 -0.712 -0.356 -0.351 -0.702              
Timepoint3:Group1  0.490 -0.348 -0.697 -0.353 -0.707  0.496       
Timepoint4:Group1  0.487 -0.346 -0.356 -0.702 -0.702  0.493  0.497

Standardized Within-Group Residuals:
       Min         Q1        Med         Q3        Max 
-2.4892418 -0.5591579  0.1000045  0.5902624  2.2719072 

Number of Observations: 150
Number of Groups: 20 
>
> residuals_model <- resid(mixed_model)
>
> hist(residuals_model, breaks = 20, main = "Histogram of Model Residuals
PBR 20-25",
+      xlab = "Residuals", col = "skyblue", border = "black")
>
> shapiro_test <- shapiro.test(residuals_model)
> print(shapiro_test)

	Shapiro-Wilk normality test

data:  residuals_model
W = 0.98657, p-value = 0.1557
```

```
> #CI
> intervals(mixed_model_prb2025, which = "fixed", level = 0.95)
Approximate 95% confidence intervals

 Fixed effects:
                        lower         est.     upper
(Intercept)        2.75880310  2.917333333 3.0758636
Timepoint2        -0.15520411  0.065166667 0.2855374
Timepoint3        -0.01753745  0.202833333 0.4232041
Timepoint4        -0.21553764  0.007794417 0.2311265
Group1            -0.15403745  0.066333333 0.2867041
Timepoint2:Group1 -0.35181801 -0.040166667 0.2714847
Timepoint3:Group1 -0.33616399 -0.022410074 0.2913438
Timepoint4:Group1 -0.33388930 -0.015732581 0.3024241
```

Effects of PCV on Glycocheck parameters

VVD

```
> mixed_model_PCV_VVD <- lme(density ~ PCV, random = ~1 | ID1, data = VO_c
ombined_data)
> summary(mixed_model_PCV_VVD)
Linear mixed-effects model fit by REML
  Data: VO_combined_data
      AIC      BIC    logLik
  1872.66 1884.807 -932.3298

Random effects:
 Formula: ~1 | ID1
        (Intercept) Residual
StdDev:  0.01833351  98.8056

Fixed effects:  density ~ PCV
                Value Std.Error  DF
(Intercept) 260.02940  61.93762 135
PCV           5.74672   1.91663 135
             t-value p-value
(Intercept) 4.198247  0.0000
PCV         2.998349  0.0032
 Correlation:
    (Intr)
PCV -0.992

Standardized Within-Group Residuals:
        Min          Q1         Med
-2.88867993 -0.57838544  0.03585045
         Q3         Max
 0.68615048  2.54340532

Number of Observations: 156
Number of Groups: 20

> residuals_model <- resid(mixed_model_PCV_VVD)
> hist(residuals_model, breaks = 20, main = "Histogram of Model Residuals"
,xlab = "Residuals", col = "skyblue", border = "black")
> shapiro_test <- shapiro.test(residuals_model)
> print(shapiro_test)

        Shapiro-Wilk normality test

data:  residuals_model
W = 0.98773, p-value = 0.1882

> intervals(mixed_model_PCV_VVD, which = "fixed", level = 0.95)
Approximate 95% confidence intervals

 Fixed effects:
```

```
                  lower       est.
(Intercept) 137.535851 260.029401
PCV           1.956218   5.746716
                  upper
(Intercept) 382.522950
PCV           9.537214
```

RBC filling

```
> mixed_model_PCV_RBCfilling <- lme(rbcfilling ~ PCV, random = ~1 | ID1, d
ata = VO_combined_data)
> summary(mixed_model_PCV_RBCfilling)
Linear mixed-effects model fit by REML
  Data: VO_combined_data
        AIC       BIC   logLik
  -440.3902 -428.2424 224.1951

Random effects:
 Formula: ~1 | ID1
         (Intercept)   Residual
StdDev: 7.52156e-07 0.05410938

Fixed effects:  rbcfilling ~ PCV
                Value  Std.Error  DF
(Intercept) 0.4800164 0.03391919 135
PCV         0.0037592 0.00104961 135
              t-value p-value
(Intercept) 14.151766   0e+00
PCV          3.581535   5e-04
 Correlation:
    (Intr)
PCV -0.992

Standardized Within-Group Residuals:
        Min          Q1         Med
-2.54656506 -0.58371825 -0.04066759
         Q3         Max
 0.45729604  2.66886906

Number of Observations: 156
Number of Groups: 20
>
> residuals_model <- resid(mixed_model_PCV_RBCfilling)
> hist(residuals_model, breaks = 20, main = "Histogram of Model Residuals"
,xlab = "Residuals", col = "skyblue", border = "black")
> shapiro_test <- shapiro.test(residuals_model)
> print(shapiro_test)

        Shapiro-Wilk normality test

data:  residuals_model
W = 0.97828, p-value = 0.01454

>
> intervals(mixed_model_PCV_RBCfilling, which = "fixed", level = 0.95)
Approximate 95% confidence intervals

 Fixed effects:
                  lower       est.
(Intercept) 0.412934716 0.48001644
PCV         0.001683412 0.00375922
                  upper
(Intercept) 0.547098158
PCV         0.005835029
```

Median P50

```
> mixed_model_PCV_P50 <- lme(medianp50 ~ PCV, random = ~1 | ID1, data = VO
_combined_data)
```

```
> summary(mixed_model_PCV_P50)
Linear mixed-effects model fit by REML
  Data: VO_combined_data
       AIC      BIC    logLik
  532.0574 544.2052 -262.0287

Random effects:
 Formula: ~1 | ID1
        (Intercept) Residual
StdDev:   0.6326778 1.183843

Fixed effects:  medianp50 ~ PCV
                Value Std.Error  DF
(Intercept)  9.662917 0.8052249 135
PCV         -0.029552 0.0245266 135
              t-value p-value
(Intercept) 12.000271  0.0000
PCV         -1.204895  0.2304
 Correlation:
    (Intr)
PCV -0.977

Standardized Within-Group Residuals:
        Min          Q1         Med
-2.05986799 -0.54473689  0.05546778
         Q3         Max
 0.57631183  3.08544957

Number of Observations: 156
Number of Groups: 20
>
> residuals_model <- resid(mixed_model_PCV_P50)
> hist(residuals_model, breaks = 20, main = "Histogram of Model Residuals"
,xlab = "Residuals", col = "skyblue", border = "black")
> shapiro_test <- shapiro.test(residuals_model)
> print(shapiro_test)

	Shapiro-Wilk normality test

data:  residuals_model
W = 0.98431, p-value = 0.07439

>
> intervals(mixed_model_PCV_P50, which = "fixed", level = 0.95)
Approximate 95% confidence intervals

 Fixed effects:
                  lower        est.
(Intercept)  8.07042968  9.66291668
PCV         -0.07805805 -0.02955198
                  upper
(Intercept) 11.25540368
PCV          0.01895409
```

PBR 5-25

```
> mixed_model_PCV_PBR525 <- lme(prb525 ~ PCV, random = ~1 | ID1, data = VO
_combined_data)
> summary(mixed_model_PCV_PBR525)
Linear mixed-effects model fit by REML
  Data: VO_combined_data
        AIC       BIC   logLik
  -59.76226 -47.61445 33.88113

Random effects:
 Formula: ~1 | ID1
         (Intercept)  Residual
StdDev: 1.120532e-05 0.1861984
```

```
Fixed effects:  prb525 ~ PCV
                 Value  Std.Error  DF   t-value p-value
(Intercept)  2.908353 0.11672094 135 24.917152  0.0000
PCV         -0.010989 0.00361187 135 -3.042459  0.0028
 Correlation:
    (Intr)
PCV -0.992

Standardized Within-Group Residuals:
        Min          Q1         Med          Q3         Max
-3.05281196 -0.58601530  0.06637839  0.68439039  3.06634782

Number of Observations: 156
Number of Groups: 20

> residuals_model <- resid(mixed_model_PCV_PBR525)
> hist(residuals_model, breaks = 20, main = "Histogram of Model Residuals"
,xlab = "Residuals", col = "skyblue", border = "black")
> shapiro_test <- shapiro.test(residuals_model)
> print(shapiro_test)

        Shapiro-Wilk normality test

data:  residuals_model
W = 0.97148, p-value = 0.002544
> intervals(mixed_model_PCV_PBR525, which = "fixed", level = 0.95)
Approximate 95% confidence intervals

 Fixed effects:
                   lower         est.        upper
(Intercept)  2.67751529  2.90835339  3.139191497
PCV         -0.01813212 -0.01098896 -0.003845796
```

PBR 5-9

```
> mixed_model_PCV_PBR59 <- lme(prb59 ~ PCV, random = ~1 | ID1, data = VO_c
ombined_data)
> summary(mixed_model_PCV_PBR59)
Linear mixed-effects model fit by REML
  Data: VO_combined_data
        AIC       BIC    logLik
  -193.4122 -181.2644 100.7061

Random effects:
 Formula: ~1 | ID1
        (Intercept)  Residual
StdDev:  0.01532998 0.1197653

Fixed effects:  prb59 ~ PCV
                  Value  Std.Error  DF   t-value p-value
(Intercept)  1.4648313 0.07591188 135 19.296469  0.0000
PCV         -0.0029601 0.00234665 135 -1.261433  0.2093
 Correlation:
    (Intr)
PCV -0.991

Standardized Within-Group Residuals:
        Min          Q1         Med          Q3         Max
-3.54946021 -0.50247329  0.05649006  0.58473254  3.34889269

Number of Observations: 156
Number of Groups: 20
>
> residuals_model <- resid(mixed_model_PCV_PBR59)
> hist(residuals_model, breaks = 20, main = "Histogram of Model Residuals"
,xlab = "Residuals", col = "skyblue", border = "black")
> shapiro_test <- shapiro.test(residuals_model)
> print(shapiro_test)
```

```
        Shapiro-Wilk normality test

data:  residuals_model
W = 0.96698, p-value = 0.0008617

>
> intervals(mixed_model_PCV_PBR59, which = "fixed", level = 0.95)
Approximate 95% confidence intervals

 Fixed effects:
                   lower          est.        upper
(Intercept)  1.314700962  1.464831307 1.614961652
PCV         -0.007601103 -0.002960145 0.001680813
```

PBR 10-19

```
> mixed_model_PCV_PBR1019 <- lme(prb1019 ~ PCV, random = ~1 | ID1, data =
VO_combined_data)
> summary(mixed_model_PCV_PBR1019)
Linear mixed-effects model fit by REML
  Data: VO_combined_data
        AIC       BIC   logLik
  -23.55032 -11.40251 15.77516

Random effects:
 Formula: ~1 | ID1
         (Intercept)  Residual
StdDev: 6.793593e-06 0.2094288

Fixed effects:  prb1019 ~ PCV
                Value  Std.Error  DF   t-value p-value
(Intercept)  3.339763 0.13128325 135 25.439369  0.0000
PCV         -0.013303 0.00406249 135 -3.274577  0.0013
 Correlation:
    (Intr)
PCV -0.992

Standardized Within-Group Residuals:
        Min          Q1         Med          Q3         Max
-3.04439822 -0.59632246  0.09111463  0.65303523  2.71803120

Number of Observations: 156
Number of Groups: 20
>
> residuals_model <- resid(mixed_model_PCV_PBR1019)
> hist(residuals_model, breaks = 20, main = "Histogram of Model Residuals"
,xlab = "Residuals", col = "skyblue", border = "black")
> shapiro_test <- shapiro.test(residuals_model)
> print(shapiro_test)

        Shapiro-Wilk normality test

data:  residuals_model
W = 0.98333, p-value = 0.05683

>
> intervals(mixed_model_PCV_PBR1019, which = "fixed", level = 0.95)
Approximate 95% confidence intervals

 Fixed effects:
                 lower        est.        upper
(Intercept)  3.0801252  3.33976308  3.599400950
PCV         -0.0213373 -0.01330294 -0.005268584
```

PBR 20-25

```
> mixed_model_PCV_PBR2025 <- lme(prb2025 ~ PCV, random = ~1 | ID1, data =
VO_combined_data)
```

```
> summary(mixed_model_PCV_PBR2025)
Linear mixed-effects model fit by REML
  Data: VO_combined_data
       AIC      BIC    logLik
  140.2831 152.4309 -66.14156

Random effects:
 Formula: ~1 | ID1
        (Intercept)  Residual
StdDev:  0.06380548 0.3515423

Fixed effects:  prb2025 ~ PCV
                Value  Std.Error  DF   t-value p-value
(Intercept)  3.416042 0.22489648 135 15.189397  0.0000
PCV         -0.012676 0.00694523 135 -1.825134  0.0702
 Correlation:
    (Intr)
PCV -0.99

Standardized Within-Group Residuals:
       Min         Q1        Med         Q3        Max
-2.7739355 -0.6267111  0.1290047  0.6477150  2.6171260

Number of Observations: 156
Number of Groups: 20
>
> residuals_model <- resid(mixed_model_PCV_PBR2025)
> hist(residuals_model, breaks = 20, main = "Histogram of Model Residuals"
,xlab = "Residuals", col = "skyblue", border = "black")
> shapiro_test <- shapiro.test(residuals_model)
> print(shapiro_test)

        Shapiro-Wilk normality test

data:  residuals_model
W = 0.98171, p-value = 0.03652

>
> intervals(mixed_model_PCV_PBR2025, which = "fixed", level = 0.95)
Approximate 95% confidence intervals

 Fixed effects:
                  lower        est.       upper
(Intercept)  2.97126592  3.41604196 3.860817993
PCV         -0.02641151 -0.01267598 0.001059551
```

Correlation between PCV and GlycoCheck parameters

```
> correlation_test<-cor.test(VO_combined_data$density, VO_combined_data$PC
V)
> print(correlation_test)

        Pearson's product-moment correlation

data:  VO_combined_data$density and VO_combined_data$PCV
t = 2.9983, df = 154, p-value = 0.003166
alternative hypothesis: true correlation is not equal to 0
95 percent confidence interval:
 0.08069321 0.37804479
sample estimates:
      cor
0.2348559
```

```
> correlation_test<-cor.test(VO_combined_data$rbcfilling, VO_combined_data
$PCV)
> print(correlation_test)

        Pearson's product-moment correlation

data:  VO_combined_data$rbcfilling and VO_combined_data$PCV
t = 3.5815, df = 154, p-value = 0.0004575
alternative hypothesis: true correlation is not equal to 0
95 percent confidence interval:
 0.1256240 0.4162922
sample estimates:
      cor
0.2772909


> correlation_test<-cor.test(VO_combined_data$density, VO_combined_data$PC
V)
> print(correlation_test)

        Pearson's product-moment correlation

data:  VO_combined_data$density and VO_combined_data$PCV
t = 2.9983, df = 154, p-value = 0.003166
alternative hypothesis: true correlation is not equal to 0
95 percent confidence interval:
 0.08069321 0.37804479
sample estimates:
      cor
0.2348559

> correlation_test<-cor.test(VO_combined_data$medianp50, VO_combined_data$
PCV)
> print(correlation_test)

        Pearson's product-moment correlation

data:  VO_combined_data$medianp50 and VO_combined_data$PCV
t = -0.87717, df = 154, p-value = 0.3818
alternative hypothesis: true correlation is not equal to 0
95 percent confidence interval:
 -0.22515432  0.08760316
sample estimates:
        cor
-0.07050821


> correlation_test<-cor.test(VO_combined_data$prb525, VO_combined_data$PCV
)
> print(correlation_test)

        Pearson's product-moment correlation

data:  VO_combined_data$prb525 and VO_combined_data$PCV
t = -3.0425, df = 154, p-value = 0.002759
alternative hypothesis: true correlation is not equal to 0
95 percent confidence interval:
 -0.3810010 -0.0841234
sample estimates:
       cor
-0.2381164

> correlation_test<-cor.test(VO_combined_data$prb59, VO_combined_data$PCV)
> print(correlation_test)

        Pearson's product-moment correlation

data:  VO_combined_data$prb59 and VO_combined_data$PCV
t = -1.2773, df = 154, p-value = 0.2034
alternative hypothesis: true correlation is not equal to 0
```

```
95 percent confidence interval:
 -0.25541769  0.05564974
sample estimates:
       cor
-0.1023864
```

```
> correlation_test<-cor.test(VO_combined_data$prb1019, VO_combined_data$PC
V)
> print(correlation_test)

	Pearson's product-moment correlation

data:  VO_combined_data$prb1019 and VO_combined_data$PCV
t = -3.2746, df = 154, p-value = 0.001307
alternative hypothesis: true correlation is not equal to 0
95 percent confidence interval:
 -0.3963883 -0.1020923
sample estimates:
       cor
-0.2551399
```

```
> correlation_test<-cor.test(VO_combined_data$prb2025, VO_combined_data$PC
V)
> print(correlation_test)

	Pearson's product-moment correlation

data:  VO_combined_data$prb2025 and VO_combined_data$PCV
t = -1.8474, df = 154, p-value = 0.06661
alternative hypothesis: true correlation is not equal to 0
95 percent confidence interval:
 -0.29749971  0.01013276
sample estimates:
       cor
-0.1472424
```

Kendall Tau – PBR 20-25 not normally distributed (Spearman's Rank not appropriate because of repeated values)

```
> correlation_test <- cor.test(VO_combined_data$prb2025, VO_combined_data$
PCV, method = "kendall")
> print(correlation_test)

	Kendall's rank correlation tau

data:  VO_combined_data$prb2025 and VO_combined_data$PCV
z = -1.9801, p-value = 0.04769
alternative hypothesis: true tau is not equal to 0
sample estimates:
       tau
-0.1108105
```

Interactions PCV Timepoint and Group

```
> VO_combined_data$Group <- relevel(as.factor(VO_combined_data$Group), ref
= "2")
> VO_combined_data$Timepoint <- as.factor(VO_combined_data$Timepoint)
> mixed_modelPCV <- lme(PCV ~ Timepoint * Group, random = ~1 | ID1, data =
VO_combined_data)
> summary(mixed_modelPCV)
Linear mixed-effects model fit by REML
  Data: VO_combined_data
       AIC      BIC    logLik
  754.5449 784.517 -367.2724

Random effects:
 Formula: ~1 | ID1
        (Intercept) Residual
StdDev:    1.669725 2.417982
```

```
Fixed effects:  PCV ~ Timepoint * Group 
                     Value Std.Error  DF
(Intercept)       32.70000 0.6570625 129
Timepoint2        -3.35000 0.7646332 129
Timepoint3         1.95000 0.7646332 129
Timepoint4         2.23373 0.7757210 129
Group1             0.40000 0.7646332 129
Timepoint2:Group1 -4.20000 1.0813546 129
Timepoint3:Group1 -2.55405 1.0893184 129
Timepoint4:Group1 -0.92000 1.1059440 129
                   t-value p-value
(Intercept)       49.76696  0.0000
Timepoint2        -4.38119  0.0000
Timepoint3         2.55024  0.0119
Timepoint4         2.87956  0.0047
Group1             0.52313  0.6018
Timepoint2:Group1 -3.88402  0.0002
Timepoint3:Group1 -2.34463  0.0206
Timepoint4:Group1 -0.83187  0.4070
 Correlation: 
                  (Intr) Tmpnt2 Tmpnt3
Timepoint2        -0.582              
Timepoint3        -0.582  0.500       
Timepoint4        -0.574  0.493  0.493
Group1            -0.582  0.500  0.500
Timepoint2:Group1  0.411 -0.707 -0.354
Timepoint3:Group1  0.408 -0.351 -0.702
Timepoint4:Group1  0.402 -0.346 -0.346
                  Tmpnt4 Group1 Tm2:G1
Timepoint2                            
Timepoint3                            
Timepoint4                            
Group1             0.493              
Timepoint2:Group1 -0.348 -0.707       
Timepoint3:Group1 -0.346 -0.702  0.496
Timepoint4:Group1 -0.702 -0.691  0.489
                  Tm3:G1
Timepoint2              
Timepoint3              
Timepoint4              
Group1                  
Timepoint2:Group1       
Timepoint3:Group1       
Timepoint4:Group1  0.487

Standardized Within-Group Residuals:
        Min          Q1         Med 
-2.80147066 -0.63052345  0.01150098 
         Q3         Max 
 0.65710438  1.96987422 

Number of Observations: 156
Number of Groups: 20 

> residuals_model <- resid(mixed_modelPCV)
> hist(residuals_model, breaks = 20, main = "Histogram of Model Residuals"
,xlab = "Residuals", col = "skyblue", border = "black")
> shapiro_test <- shapiro.test(residuals_model)
> print(shapiro_test)

        Shapiro-Wilk normality test

data:  residuals_model
W = 0.99151, p-value = 0.4809

> intervals(mixed_modelPCV, which = "fixed", level = 0.95)
Approximate 95% confidence intervals

 Fixed effects:
```

```
                       lower       est.
(Intercept)       31.3999858 32.7000000
Timepoint2        -4.8628455 -3.3500000
Timepoint3         0.4371545  1.9500000
Timepoint4         0.6989503  2.2337332
Group1            -1.1128455  0.4000000
Timepoint2:Group1 -6.3394866 -4.2000000
Timepoint3:Group1 -4.7092962 -2.5540530
Timepoint4:Group1 -3.1081366 -0.9199993
                       upper
(Intercept)       34.0000142
Timepoint2        -1.8371545
Timepoint3         3.4628455
Timepoint4         3.7685162
Group1             1.9128455
Timepoint2:Group1 -2.0605134
Timepoint3:Group1 -0.3988098
Timepoint4:Group1  1.2681380
```

Interactions TS Timepoint and Group

```
> mixed_modelTS <- lme(TS ~ Timepoint * Group, random = ~1 | ID1, data = V
O_combined_data)
> summary(mixed_modelTS)
Linear mixed-effects model fit by REML
  Data: VO_combined_data
       AIC      BIC   logLik
  858.8401 888.8122 -419.42

Random effects:
 Formula: ~1 | ID1
        (Intercept) Residual
StdDev:    3.006815 3.344332

Fixed effects:  TS ~ Timepoint * Group
                     Value Std.Error  DF
(Intercept)       69.95000  1.005621 129
Timepoint2        -2.20000  1.057571 129
Timepoint3         2.60000  1.057571 129
Timepoint4         0.60549  1.073064 129
Group1             0.80000  1.057571 129
Timepoint2:Group1 -12.45000  1.495631 129
Timepoint3:Group1  -5.81218  1.506789 129
Timepoint4:Group1  -1.03350  1.530049 129
                   t-value p-value
(Intercept)       69.55898  0.0000
Timepoint2        -2.08024  0.0395
Timepoint3         2.45846  0.0153
Timepoint4         0.56426  0.5736
Group1             0.75645  0.4508
Timepoint2:Group1 -8.32425  0.0000
Timepoint3:Group1 -3.85733  0.0002
Timepoint4:Group1 -0.67547  0.5006
 Correlation:
                  (Intr) Tmpnt2 Tmpnt3
Timepoint2        -0.526
Timepoint3        -0.526  0.500
Timepoint4        -0.518  0.493  0.493
Group1            -0.526  0.500  0.500
Timepoint2:Group1  0.372 -0.707 -0.354
Timepoint3:Group1  0.369 -0.351 -0.702
Timepoint4:Group1  0.363 -0.346 -0.346
                  Tmpnt4 Group1 Tm2:G1
Timepoint2
Timepoint3
Timepoint4
Group1             0.493
Timepoint2:Group1 -0.348 -0.707
```

```
Timepoint3:Group1 -0.346 -0.702  0.496
Timepoint4:Group1 -0.702 -0.691  0.489
                  Tm3:G1
Timepoint2
Timepoint3
Timepoint4
Group1
Timepoint2:Group1
Timepoint3:Group1
Timepoint4:Group1  0.487

Standardized Within-Group Residuals:
         Min           Q1          Med
-2.699972293 -0.554314062  0.002080096
          Q3          Max
 0.498919846  2.554288080

Number of Observations: 156
Number of Groups: 20

> residuals_model <- resid(mixed_modelTS)
> hist(residuals_model, breaks = 20, main = "Histogram of Model Residuals"
,xlab = "Residuals", col = "skyblue", border = "black")
> shapiro_test <- shapiro.test(residuals_model)
> print(shapiro_test)

        Shapiro-Wilk normality test

data:  residuals_model
W = 0.98495, p-value = 0.08875

> intervals(mixed_modelTS, which = "fixed", level = 0.95)
Approximate 95% confidence intervals

 Fixed effects:
                        lower        est.
(Intercept)        67.9603534  69.9500000
Timepoint2         -4.2924293  -2.2000000
Timepoint3          0.5075707   2.6000000
Timepoint4         -1.5175929   0.6054905
Group1             -1.2924293   0.8000000
Timepoint2:Group1 -15.4091419 -12.4500000
Timepoint3:Group1  -8.7934007  -5.8121820
Timepoint4:Group1  -4.0607407  -1.0335005
                       upper
(Intercept)       71.9396466
Timepoint2        -0.1075707
Timepoint3         4.6924293
Timepoint4         2.7285739
Group1             2.8924293
Timepoint2:Group1 -9.4908581
Timepoint3:Group1 -2.8309633
Timepoint4:Group1  1.9937397
```

Anaesthetic variable analysis

Data normality

```
> # Shapiro-Wilk normality tests
> shapiro.test(na.omit(Glycocheck_volume_overload_cats_dataset$MAP))

	Shapiro-Wilk normality test

data:  na.omit(Glycocheck_volume_overload_cats_dataset$MAP)
W = 0.97501, p-value = 0.006925

> shapiro.test(na.omit(Glycocheck_volume_overload_cats_dataset$HR))

	Shapiro-Wilk normality test

data:  na.omit(Glycocheck_volume_overload_cats_dataset$HR)
W = 0.98504, p-value = 0.09309

> shapiro.test(na.omit(Glycocheck_volume_overload_cats_dataset$Temp))

	Shapiro-Wilk normality test

data:  na.omit(Glycocheck_volume_overload_cats_dataset$Temp)
W = 0.97829, p-value = 0.01726

> shapiro.test(na.omit(Glycocheck_volume_overload_cats_dataset$SAP))

	Shapiro-Wilk normality test

data:  na.omit(Glycocheck_volume_overload_cats_dataset$SAP)
W = 0.96646, p-value = 0.0008786

> shapiro.test(na.omit(Glycocheck_volume_overload_cats_dataset$DAP))

	Shapiro-Wilk normality test

data:  na.omit(Glycocheck_volume_overload_cats_dataset$DAP)
W = 0.97026, p-value = 0.002149


> hist(Glycocheck_volume_overload_cats_dataset$MAP,
+      main = "MAP",
+      xlab = "MAP")
>
> hist(Glycocheck_volume_overload_cats_dataset$HR,
+      main = "HR",
+      xlab = "HR")
>
> hist(Glycocheck_volume_overload_cats_dataset$Temp,
+      main = "Temperature",
+      xlab = "Temp")

> hist(Glycocheck_volume_overload_cats_dataset$SAP,
+      main = "SAP",
+      xlab = "SAP")
```

hist(Glycocheck_volume_overload_cats_dataset$SAP,

main = "SAP",

xlab = "SAP")

Linear mixed effects modelling for effects of HR, MAP and Temp on PBRs

```
> model_HR <- lme(
+   fixed = prb525 ~ HR,
+   random = ~1 | ID1,
+   data = Glycocheck_volume_overload_cats_dataset,
+   na.action = na.omit
+ )
> summary(model_HR)
Linear mixed-effects model fit by REML
  Data: Glycocheck_volume_overload_cats_dataset
        AIC       BIC   logLik
  -46.33512 -34.21337 27.16756

Random effects:
 Formula: ~1 | ID1
        (Intercept) Residual
StdDev: 0.007610988 0.192078

Fixed effects:  prb525 ~ HR
                Value  Std.Error  DF   t-value p-value
(Intercept) 2.5210308 0.09767330 134 25.810850  0.0000
HR          0.0002762 0.00076492 134  0.361064  0.7186
 Correlation:
   (Intr)
HR -0.987

Standardized Within-Group Residuals:
       Min         Q1        Med         Q3        Max
-3.2038357 -0.5392576  0.1226721  0.5969402  2.9228299

Number of Observations: 155
Number of Groups: 20
> res_HR   <- residuals(model_HR)
> shapiro.test(res_HR)

        Shapiro-Wilk normality test

data:  res_HR
W = 0.96537, p-value = 0.0006224

> hist(res_HR,
+      main = "HR model residuals",
+      xlab = "Normalized residuals")

#CI - HR
> coef_HR <- summary(model_HR)$tTable
```

```
> coef_HR <- cbind(
+   coef_HR,
+   Lower_CI = coef_HR[, "Value"] - 1.96 * coef_HR[, "Std.Error"],
+   Upper_CI = coef_HR[, "Value"] + 1.96 * coef_HR[, "Std.Error"]
+ )
> coef_HR
                   Value    Std.Error  DF    t-value      p-value     Lowe
r_CI
(Intercept) 2.5210308434 0.0976732963 134 25.8108504 7.558806e-54  2.32959
1183
HR          0.0002761855 0.0007649205 134  0.3610643 7.186200e-01 -0.00122
3059
              Upper_CI
(Intercept) 2.71247050
HR          0.00177543


> model_MAP <- lme(
+   fixed = prb525 ~ MAP,
+   random = ~1 | ID1,
+   data = Glycocheck_volume_overload_cats_dataset,
+   na.action = na.omit
+ )
> summary(model_MAP)
Linear mixed-effects model fit by REML
  Data: Glycocheck_volume_overload_cats_dataset
        AIC       BIC   logLik
  -46.82595 -34.75683 27.41298

Random effects:
 Formula: ~1 | ID1
         (Intercept)  Residual
StdDev: 2.551946e-05 0.1920238

Fixed effects:  prb525 ~ MAP
                Value  Std.Error  DF   t-value p-value
(Intercept)  2.652574 0.11117654 132 23.859114  0.0000
MAP         -0.001177 0.00131653 132 -0.894048  0.3729
 Correlation:
    (Intr)
MAP -0.99

Standardized Within-Group Residuals:
        Min          Q1         Med          Q3         Max
-3.34715114 -0.48699235  0.09205891  0.58318402  2.95626184

Number of Observations: 153
Number of Groups: 20

> res_MAP  <- residuals(model_MAP)
> shapiro.test(res_MAP)

	Shapiro-Wilk normality test

data:  res_MAP
W = 0.96268, p-value = 0.0003748

> hist(res_MAP,
```

```
+      main = "MAP model residuals",
+      xlab = "Normalized residuals")

#CI - MAP
> coef_MAP <- summary(model_MAP)$tTable
> coef_MAP <- cbind(
+   coef_MAP,
+   Lower_CI = coef_MAP[, "Value"] - 1.96 * coef_MAP[, "Std.Error"],
+   Upper_CI = coef_MAP[, "Value"] + 1.96 * coef_MAP[, "Std.Error"]
+ )
> coef_MAP
                   Value   Std.Error  DF   t-value      p-value     Lower_
CI
(Intercept)  2.652573689 0.111176537 132 23.859114 1.035946e-49  2.4346676
76
MAP         -0.001177041 0.001316531 132 -0.894048 3.729234e-01 -0.0037574
41
               Upper_CI
(Intercept) 2.870479702
MAP         0.001403358



> model_Temp <- lme(
+   fixed = prb525 ~ Temp,
+   random = ~1 | ID1,
+   data = Glycocheck_volume_overload_cats_dataset,
+   na.action = na.omit
+ )
> summary(model_Temp)
Linear mixed-effects model fit by REML
  Data: Glycocheck_volume_overload_cats_dataset
        AIC       BIC   logLik
  -47.67264 -35.65685 27.83632

Random effects:
 Formula: ~1 | ID1
        (Intercept)  Residual
StdDev:  0.01859019 0.1935464

Fixed effects:  prb525 ~ Temp
                 Value Std.Error  DF   t-value p-value
(Intercept)  2.5912237 0.7288050 130  3.555442  0.0005
Temp        -0.0009956 0.0196507 130 -0.050666  0.9597
 Correlation:
     (Intr)
Temp -1

Standardized Within-Group Residuals:
       Min         Q1        Med         Q3        Max
-3.2149253 -0.5302588  0.1196210  0.6063320  2.8754645

Number of Observations: 151
Number of Groups: 20
> res_Temp <- residuals(model_Temp)
> hist(res_Temp,
+      main = "Temp model residuals",
+      xlab = "Normalized residuals")
```

```
> qqnorm(res_MAP,
+        main = "Q-Q plot: MAP model residuals")
> qqline(res_MAP)
> model59_HR <- lme(
+   fixed = prb59 ~ HR,
+   random = ~1 | ID,
+   data = Glycocheck_volume_overload_cats_dataset,
+   na.action = na.omit
+ )

> coef_Temp <- summary(model_Temp)$tTable
> coef_Temp <- cbind(
+   coef_Temp,
+   Lower_CI = coef_Temp[, "Value"] - 1.96 * coef_Temp[, "Std.Error"],
+   Upper_CI = coef_Temp[, "Value"] + 1.96 * coef_Temp[, "Std.Error"]
+ )
> coef_Temp
                     Value  Std.Error  DF     t-value      p-value    Lower
_CI
(Intercept)  2.5912237120 0.72880497 130  3.55544189 0.0005269625  1.16276
597
Temp        -0.0009956239 0.01965073 130 -0.05066599 0.9596694421 -0.03951
106
              Upper_CI
(Intercept) 4.01968145
Temp        0.03751981


> model59_HR <- lme(
+   fixed = prb59 ~ HR,
+   random = ~1 | ID1,
+   data = Glycocheck_volume_overload_cats_dataset,
+   na.action = na.omit
+ )
> summary(model59_HR)
Linear mixed-effects model fit by REML
  Data: Glycocheck_volume_overload_cats_dataset
        AIC       BIC   logLik
  -188.8573 -176.7355 98.42863

Random effects:
 Formula: ~1 | ID1
        (Intercept)  Residual
StdDev:  0.02039192 0.1191452

Fixed effects:  prb59 ~ HR
                 Value  Std.Error  DF   t-value p-value
(Intercept)  1.4483052 0.06206874 134 23.333892  0.0000
HR          -0.0006152 0.00048512 134 -1.268183  0.2069
 Correlation:
   (Intr)
HR -0.985

Standardized Within-Group Residuals:
        Min          Q1         Med          Q3         Max
-3.54947208 -0.47175482  0.02210588  0.59136243  3.27758901

Number of Observations: 155
```

```
Number of Groups: 20
> res59_HR   <- residuals(model59_HR)
> shapiro.test(res59_HR)

	Shapiro-Wilk normality test

data:  res59_HR
W = 0.96238, p-value = 0.0003168

> hist(res59_HR,
+      main = "HR model residuals",
+      xlab = "Residuals")

> coef_59HR <- summary(model59_HR)$tTable
> coef_59HR <- cbind(
+   coef_59HR,
+   Lower_CI = coef_59HR[, "Value"] - 1.96 * coef_59HR[, "Std.Error"],
+   Upper_CI = coef_59HR[, "Value"] + 1.96 * coef_59HR[, "Std.Error"]
+ )
> coef_59HR
                    Value    Std.Error  DF   t-value      p-value     Lowe
r_CI
(Intercept)  1.4483052065 0.0620687359 134 23.333892 4.875490e-49  1.32665
0484
HR          -0.0006152176 0.0004851172 134 -1.268183 2.069315e-01 -0.00156
6047
                Upper_CI
(Intercept) 1.5699599287
HR          0.0003356122


> model59_MAP <- lme(
+   fixed = prb59 ~ MAP,
+   random = ~1 | ID1,
+   data = Glycocheck_volume_overload_cats_dataset,
+   na.action = na.omit
+ )
> summary(model59_MAP)
Linear mixed-effects model fit by REML
  Data: Glycocheck_volume_overload_cats_dataset
        AIC       BIC   logLik
  -183.8893 -171.8201 95.94463

Random effects:
 Formula: ~1 | ID1
        (Intercept)  Residual
StdDev:  0.01787931 0.1208004

Fixed effects:  prb59 ~ MAP
                Value  Std.Error  DF   t-value p-value
(Intercept)  1.423549 0.07120815 132 19.991379  0.0000
MAP         -0.000629 0.00084205 132 -0.747011  0.4564
 Correlation:
    (Intr)
MAP -0.989

Standardized Within-Group Residuals:
        Min          Q1         Med          Q3         Max
```

```
-3.62331028 -0.47746800  0.05849491  0.53662735  3.29305265

Number of Observations: 153
Number of Groups: 20
> res59_MAP  <- residuals(model59_MAP)
> shapiro.test(res59_MAP)

        Shapiro-Wilk normality test

data:  res59_MAP
W = 0.96482, p-value = 0.0006043

> hist(res59_MAP,
+      main = "MAP model residuals",
+      xlab = "Residuals")

> coef59_MAP <- summary(model59_MAP)$tTable
> coef59_MAP <- cbind(
+   coef59_MAP,
+   Lower_CI = coef59_MAP[, "Value"] - 1.96 * coef59_MAP[, "Std.Error"],
+   Upper_CI = coef59_MAP[, "Value"] + 1.96 * coef59_MAP[, "Std.Error"]
+ )
> coef59_MAP
                    Value    Std.Error  DF    t-value      p-value     Low
er_CI
(Intercept)  1.4235491694 0.0712081528 132 19.9913790 9.290351e-42  1.2839
81190
MAP         -0.0006290187 0.0008420478 132 -0.7470107 4.563856e-01 -0.0022
79432
               Upper_CI
(Intercept) 1.563117149
MAP         0.001021395


> model59_Temp <- lme(
+   fixed = prb59 ~ Temp,
+   random = ~1 | ID1,
+   data = Glycocheck_volume_overload_cats_dataset,
+   na.action = na.omit
+ )
> summary(model59_Temp)
Linear mixed-effects model fit by REML
  Data: Glycocheck_volume_overload_cats_dataset
        AIC       BIC   logLik
  -186.6603 -174.6446 97.33017

Random effects:
 Formula: ~1 | ID1
        (Intercept)  Residual
StdDev:  0.02806201 0.1192679

Fixed effects:  prb59 ~ Temp
                 Value Std.Error  DF   t-value p-value
(Intercept)  1.6604626 0.4629266 130  3.586881  0.0005
Temp        -0.0078946 0.0124810 130 -0.632527  0.5282
 Correlation: 
     (Intr)
Temp -1
```

```
Standardized Within-Group Residuals:
        Min          Q1         Med          Q3         Max 
-3.38840771 -0.47924189  0.04070407  0.58385676  3.29517755 

Number of Observations: 151
Number of Groups: 20 

> res59_Temp <- residuals(model59_Temp)
> shapiro.test(res59_Temp)

	Shapiro-Wilk normality test

data:  res59_Temp
W = 0.96548, p-value = 0.0007728

> hist(res59_Temp,
+      main = "Temp model residuals",
+      xlab = "Residuals")

> qqnorm(res59_HR,
+        main = "HR model residuals")
> qqline(res59_HR)
> qqnorm(res59_MAP,
+        main = "MAP model residuals")
> qqline(res59_MAP)
> qqnorm(res59_Temp,
+        main = "Temp model residuals")
> qqline(res59_Temp)
> par(mfrow = c(1, 1))

> coef59_Temp <- summary(model59_Temp)$tTable
> coef59_Temp <- cbind(
+   coef59_Temp,
+   Lower_CI = coef59_Temp[, "Value"] - 1.96 * coef59_Temp[, "Std.Error"],
+   Upper_CI = coef59_Temp[, "Value"] + 1.96 * coef59_Temp[, "Std.Error"]
+ )
> coef59_Temp
                   Value Std.Error  DF    t-value      p-value    Lower_CI
(Intercept)  1.660462594 0.4629266 130  3.5868809 0.0004724281  0.75312646
Temp        -0.007894575 0.0124810 130 -0.6325273 0.5281533503 -0.03235734
              Upper_CI
(Intercept) 2.56779873
Temp        0.01656819


> # HR
> model1019_HR <- lme(
+   fixed = prb1019 ~ HR,
+   random = ~1 | ID1,
+   data = Glycocheck_volume_overload_cats_dataset,
+   na.action = na.omit
+ )
> summary(model1019_HR)
Linear mixed-effects model fit by REML
  Data: Glycocheck_volume_overload_cats_dataset 
        AIC      BIC   logLik
  -9.214072 2.90768 8.607036
```

```
Random effects:
 Formula: ~1 | ID1
        (Intercept)  Residual
StdDev: 1.087236e-05 0.2170121

Fixed effects:  prb1019 ~ HR
                Value  Std.Error  DF   t-value p-value
(Intercept) 2.8930157 0.11017427 134 26.258543  0.0000
HR          0.0001548 0.00086292 134  0.179352  0.8579
 Correlation:
   (Intr)
HR -0.987

Standardized Within-Group Residuals:
        Min          Q1         Med          Q3         Max
-3.28891573 -0.55306820  0.02595405  0.71602256  2.57318503

Number of Observations: 155
Number of Groups: 20
> res1019_HR <- residuals(model1019_HR)
> shapiro.test(res1019_HR)

	Shapiro-Wilk normality test

data:  res1019_HR
W = 0.97141, p-value = 0.00261

>
> hist(res1019_HR,
+      main = "HR model residuals",
+      xlab = "Residuals")
>
> qqnorm(res1019_HR,
+        main = "HR model residuals")
> qqline(res1019_HR)

> coef1019_HR <- summary(model1019_HR)$tTable
> coef1019_HR <- cbind(
+   coef1019_HR,
+   Lower_CI = coef1019_HR[, "Value"] - 1.96 * coef1019_HR[, "Std.Error"],
+   Upper_CI = coef1019_HR[, "Value"] + 1.96 * coef1019_HR[, "Std.Error"]
+ )
> coef1019_HR
                   Value    Std.Error  DF    t-value      p-value     Lowe
r_CI
(Intercept) 2.8930157329 0.1101742672 134 26.2585430 1.100757e-54  2.67707
4169
HR          0.0001547665 0.0008629185 134  0.1793524 8.579319e-01 -0.00153
6554
               Upper_CI
(Intercept) 3.108957297
HR          0.001846087


> model1019_MAP <- lme(
+   fixed = prb1019 ~ MAP,
+   random = ~1 | ID1,
```

```
+   data = Glycocheck_volume_overload_cats_dataset,
+   na.action = na.omit
+ )
>
> summary(model1019_MAP)
Linear mixed-effects model fit by REML
  Data: Glycocheck_volume_overload_cats_dataset
        AIC       BIC   logLik
  -13.13849 -1.069366 10.56924

Random effects:
 Formula: ~1 | ID1
         (Intercept) Residual
StdDev: 7.031949e-06 0.214684

Fixed effects:  prb1019 ~ MAP
                 Value  Std.Error  DF  t-value p-value
(Intercept)  3.0781217 0.12429618 132 24.76441  0.0000
MAP         -0.0020107 0.00147189 132 -1.36606  0.1742
 Correlation:
    (Intr)
MAP -0.99

Standardized Within-Group Residuals:
        Min          Q1         Med          Q3         Max
-3.35968173 -0.46784728  0.05142554  0.68063025  2.64956473

Number of Observations: 153
Number of Groups: 20
> res1019_MAP <- residuals(model1019_MAP)
> shapiro.test(res1019_MAP)

        Shapiro-Wilk normality test

data:  res1019_MAP
W = 0.96831, p-value = 0.001351

>
> hist(res1019_MAP,
+      main = "MAP model residuals",
+      xlab = "Residuals")
> qqnorm(res1019_MAP,
+        main = "MAP model residuals")
> qqline(res1019_MAP)

> coef1019_MAP <- summary(model1019_MAP)$tTable
> coef1019_MAP <- cbind(
+   coef1019_MAP,
+   Lower_CI = coef1019_MAP[, "Value"] - 1.96 * coef1019_MAP[, "Std.Error"
],
+   Upper_CI = coef1019_MAP[, "Value"] + 1.96 * coef1019_MAP[, "Std.Error"
]
+ )
> coef1019_MAP
                   Value   Std.Error  DF  t-value      p-value     Lower_C
I
(Intercept)  3.078121669 0.124296179 132 24.76441 1.851027e-51  2.83450115
8
```

```
MAP         -0.002010691 0.001471891 132 -1.36606 1.742429e-01 -0.00489559
7
                Upper_CI
(Intercept) 3.3217421811
MAP         0.0008742145


> model1019_Temp <- lme(
+   fixed = prb1019 ~ Temp,
+   random = ~1 | ID1,
+   data = Glycocheck_volume_overload_cats_dataset,
+   na.action = na.omit
+ )
> summary(model1019_Temp)
Linear mixed-effects model fit by REML
  Data: Glycocheck_volume_overload_cats_dataset
        AIC       BIC   logLik
  -11.78166 0.2341227 9.890831

Random effects:
 Formula: ~1 | ID1
         (Intercept)  Residual
StdDev: 1.588186e-05 0.2192453

Fixed effects:  prb1019 ~ Temp
                 Value Std.Error  DF   t-value p-value
(Intercept)  3.0491320 0.8187091 130  3.724317  0.0003
Temp        -0.0036985 0.0220751 130 -0.167540  0.8672
 Correlation:
     (Intr)
Temp -1

Standardized Within-Group Residuals:
        Min          Q1         Med          Q3         Max
-3.26571033 -0.53412102  0.04868995  0.70246926  2.55052654

Number of Observations: 151
Number of Groups: 20
> res1019_Temp <- residuals(model1019_Temp)
> shapiro.test(res1019_Temp)

        Shapiro-Wilk normality test

data:  res1019_Temp
W = 0.97193, p-value = 0.003497

>
> hist(res1019_Temp,
+      main = "Temp model residuals",
+      xlab = "Residuals")
>
> qqnorm(res1019_Temp,
+        main = "Temp model residuals")
> qqline(res1019_Temp)

> coef1019_Temp <- summary(model1019_Temp)$tTable
> coef1019_Temp <- cbind(
+   coef1019_Temp,
```

```
+   Lower_CI = coef1019_Temp[, "Value"] - 1.96 * coef1019_Temp[, "Std.Erro
r"],
+   Upper_CI = coef1019_Temp[, "Value"] + 1.96 * coef1019_Temp[, "Std.Erro
r"]
+ )
> coef1019_Temp
                   Value  Std.Error  DF    t-value      p-value    Lower_C
I
(Intercept)  3.049132009 0.81870911 130  3.7243167 0.0002907357  1.4444621
6
Temp        -0.003698475 0.02207513 130 -0.1675403 0.8672052661 -0.0469657
3
              Upper_CI
(Intercept) 4.65380185
Temp        0.03956878
```

```
#PBR 20-25
> # HR
> model2025_HR <- lme(
+   fixed = prb2025 ~ HR,
+   random = ~1 | ID1,
+   data = Glycocheck_volume_overload_cats_dataset,
+   na.action = na.omit
+ )
> summary(model2025_HR)
Linear mixed-effects model fit by REML
  Data: Glycocheck_volume_overload_cats_dataset
       AIC      BIC    logLik
  143.5448 155.6665 -67.77238

Random effects:
 Formula: ~1 | ID1
        (Intercept)  Residual
StdDev:  0.05813095 0.3533625

Fixed effects:  prb2025 ~ HR
                Value  Std.Error  DF   t-value p-value
(Intercept) 2.6734683 0.18377415 134 14.547576  0.0000
HR          0.0026656 0.00143657 134  1.855515  0.0657
 Correlation:
   (Intr)
HR -0.985

Standardized Within-Group Residuals:
       Min         Q1        Med         Q3        Max
-2.5630983 -0.5559883  0.1349147  0.6678240  2.7662136

Number of Observations: 155
Number of Groups: 20
>
> res2025_HR <- residuals(model2025_HR)
> shapiro.test(res2025_HR)

        Shapiro-Wilk normality test

data:  res2025_HR
W = 0.97598, p-value = 0.008237
```

```
>
> hist(res2025_HR,
+      main = "HR model residuals",
+      xlab = "Residuals")
>
> qqnorm(res2025_HR,
+        main = "HR model residuals")
> qqline(res2025_HR)

> coef2025_HR <- summary(model2025_HR)$tTable
> coef2025_HR <- cbind(
+   coef2025_HR,
+   Lower_CI = coef2025_HR[, "Value"] - 1.96 * coef2025_HR[, "Std.Error"],
+   Upper_CI = coef2025_HR[, "Value"] + 1.96 * coef2025_HR[, "Std.Error"]
+ )
> coef2025_HR
                  Value   Std.Error  DF   t-value     p-value      Lower_C
I
(Intercept) 2.673468331 0.183774146 134 14.547576 2.34955e-29  2.313271005
1
HR          0.002665584 0.001436574 134  1.855515 6.57198e-02 -0.000150100
4
               Upper_CI
(Intercept) 3.033665658
HR          0.005481268


> # MAP
> model2025_MAP <- lme(
+   fixed = prb2025 ~ MAP,
+   random = ~1 | ID1,
+   data = Glycocheck_volume_overload_cats_dataset,
+   na.action = na.omit
+ )
> summary(model2025_MAP)
Linear mixed-effects model fit by REML
  Data: Glycocheck_volume_overload_cats_dataset
       AIC      BIC    logLik
  145.3186 157.3877 -68.65928

Random effects:
 Formula: ~1 | ID1
        (Intercept) Residual
StdDev:  0.06802481 0.357327

Fixed effects:  prb2025 ~ MAP
                Value  Std.Error  DF   t-value p-value
(Intercept) 2.8525385 0.21266706 132 13.413166  0.0000
MAP         0.0018629 0.00251256 132  0.741419  0.4598
 Correlation:
    (Intr)
MAP -0.988

Standardized Within-Group Residuals:
       Min         Q1        Med         Q3        Max
-2.6349697 -0.5984296  0.1468773  0.6468396  2.6135313
```

```
Number of Observations: 153
Number of Groups: 20
>
> res2025_MAP <- residuals(model2025_MAP)
> shapiro.test(res2025_MAP)

        Shapiro-Wilk normality test

data:  res2025_MAP
W = 0.98258, p-value = 0.05032

>
> hist(res2025_MAP,
+      main = "MAP model residuals",
+      xlab = "Residuals")
>
> qqnorm(res2025_MAP,
+        main = "MAP model residuals")
> qqline(res2025_MAP)

> coef2025_MAP <- summary(model2025_MAP)$tTable
> coef2025_MAP <- cbind(
+   coef2025_MAP,
+   Lower_CI = coef2025_MAP[, "Value"] - 1.96 * coef2025_MAP[, "Std.Error"
],
+   Upper_CI = coef2025_MAP[, "Value"] + 1.96 * coef2025_MAP[, "Std.Error"
]
+ )
> coef2025_MAP
                  Value   Std.Error  DF    t-value      p-value     Lower_
CI
(Intercept) 2.852538479 0.212667056 132 13.4131658 2.040036e-26  2.4357110
49
MAP         0.001862862 0.002512564 132  0.7414186 4.597567e-01 -0.0030617
64
               Upper_CI
(Intercept) 3.269365909
MAP         0.006787487


> # Temp
> model2025_Temp <- lme(
+   fixed = prb2025 ~ Temp,
+   random = ~1 | ID1,
+   data = Glycocheck_volume_overload_cats_dataset,
+   na.action = na.omit
+ )
> summary(model2025_Temp)
Linear mixed-effects model fit by REML
  Data: Glycocheck_volume_overload_cats_dataset
       AIC      BIC    logLik
  137.0027 149.0185 -64.50136

Random effects:
 Formula: ~1 | ID1
        (Intercept)  Residual
StdDev:  0.03089581 0.3599857
```

```
Fixed effects:  prb2025 ~ Temp
                Value Std.Error  DF   t-value p-value
(Intercept) 0.6543641 1.3533666 130 0.4835084  0.6295
Temp        0.0634690 0.0364909 130 1.7393113  0.0843
 Correlation:
     (Intr)
Temp -1

Standardized Within-Group Residuals:
       Min         Q1        Med         Q3        Max
-2.5078855 -0.5172752  0.1608677  0.6463299  2.7450624

Number of Observations: 151
Number of Groups: 20
>
> res2025_Temp <- residuals(model2025_Temp)
> shapiro.test(res2025_Temp)

        Shapiro-Wilk normality test

data:  res2025_Temp
W = 0.98262, p-value = 0.05367

>
> hist(res2025_Temp,
+      main = "Temp model residuals",
+      xlab = "Residuals")
>
> qqnorm(res2025_Temp,
+        main = "Temp model residuals")
> qqline(res2025_Temp)

> coef2025_Temp <- summary(model2025_Temp)$tTable
> coef2025_Temp <- cbind(
+   coef2025_Temp,
+   Lower_CI = coef2025_Temp[, "Value"] - 1.96 * coef2025_Temp[, "Std.Erro
r"],
+   Upper_CI = coef2025_Temp[, "Value"] + 1.96 * coef2025_Temp[, "Std.Erro
r"]
+ )
> coef2025_Temp
                 Value  Std.Error  DF   t-value    p-value     Lower_CI   U
pper_CI
(Intercept) 0.65436407 1.35336659 130 0.4835084 0.62954821 -1.998234440 3.
3069626
Temp        0.06346897 0.03649086 130 1.7393113 0.08434704 -0.008053122 0.
1349911
```

Summary stats anaesthetic variables

```
> library(dplyr)

> Glycocheck_volume_overload_cats_dataset <-
+   Glycocheck_volume_overload_cats_dataset %>%
+   mutate(
+     across(c(HR, SAP, DAP, MAP, Temp), ~ as.numeric(as.character(.)))
+   )
```

```
> summary_stats <- Glycocheck_volume_overload_cats_dataset %>%
+   select(Group, Timepoint, HR, SAP, DAP, MAP, Temp) %>%
+   pivot_longer(
+     cols = c(HR, SAP, DAP, MAP, Temp),
+     names_to = "Variable",
+     values_to = "Value"
+   ) %>%
+   group_by(Group, Timepoint, Variable) %>%
+   summarise(
+     n = sum(!is.na(Value)),
+     Min = ifelse(all(is.na(Value)), NA, min(Value, na.rm = TRUE)),
+     Max = ifelse(all(is.na(Value)), NA, max(Value, na.rm = TRUE)),
+     Median = ifelse(all(is.na(Value)), NA, median(Value, na.rm = TRUE)),
+     IQR = ifelse(all(is.na(Value)), NA, IQR(Value, na.rm = TRUE)),
+     Mean = ifelse(all(is.na(Value)), NA, mean(Value, na.rm = TRUE)),
+     SD = ifelse(sum(!is.na(Value)) > 1, sd(Value, na.rm = TRUE), NA),
+     .groups = "drop"
+   ) %>%
+   mutate(
+     across(c(Min, Max, Median, IQR, Mean, SD), ~ round(.x, 2))
+   )
> write_xlsx(
+   summary_stats,
+   "Summary_statistics_by_group_time.xlsx"
+ )
> # Shapiro-Wilk normality tests
> shapiro.test(na.omit(Glycocheck_volume_overload_cats_dataset$MAP))

        Shapiro-Wilk normality test

data:  na.omit(Glycocheck_volume_overload_cats_dataset$MAP)
W = 0.97501, p-value = 0.006925

> shapiro.test(na.omit(Glycocheck_volume_overload_cats_dataset$HR))

        Shapiro-Wilk normality test

data:  na.omit(Glycocheck_volume_overload_cats_dataset$HR)
W = 0.98504, p-value = 0.09309

> shapiro.test(na.omit(Glycocheck_volume_overload_cats_dataset$Temp))

        Shapiro-Wilk normality test

data:  na.omit(Glycocheck_volume_overload_cats_dataset$Temp)
W = 0.97829, p-value = 0.01726

> hist(Glycocheck_volume_overload_cats_dataset$MAP,
+      main = "MAP",
+      xlab = "MAP")
>
> hist(Glycocheck_volume_overload_cats_dataset$HR,
+      main = "HR",
+      xlab = "HR")
>
> hist(Glycocheck_volume_overload_cats_dataset$Temp,
+      main = "Temperature",
+      xlab = "Temp")
```

Linear mixed effect modelling for group and timepoint effects on anaesthetic variables

```
> # HR
> model_HR <- lme(
+   HR ~ Group * Timepoint,
+   random = ~1 | ID1,
+   data = Glycocheck_volume_overload_cats_dataset,
+   na.action = na.omit
+ )
>
> summary(model_HR)
Linear mixed-effects model fit by REML
  Data: Glycocheck_volume_overload_cats_dataset
       AIC      BIC    logLik
  1255.565 1285.469 -617.7823

Random effects:
 Formula: ~1 | ID1
        (Intercept) Residual
StdDev:    9.730233 13.44277

Fixed effects:  HR ~ Group * Timepoint
                      Value Std.Error  DF   t-value p-value
(Intercept)       116.13333  3.710698 128 31.296895  0.0000
Group1             -0.35833  4.250979 128 -0.084294  0.9330
Timepoint2         -4.07500  4.250979 128 -0.958603  0.3396
Timepoint3         11.17500  4.250979 128  2.628806  0.0096
Timepoint4         32.17791  4.312752 128  7.461109  0.0000
Group1:Timepoint2   5.95269  6.055629 128  0.983000  0.3275
Group1:Timepoint3   5.19683  6.056184 128  0.858104  0.3924
Group1:Timepoint4  -5.97748  6.148830 128 -0.972133  0.3328
 Correlation:
                  (Intr) Group1 Tmpnt2 Tmpnt3 Tmpnt4 Gr1:T2 Gr1:T3
Group1            -0.573
Timepoint2        -0.573  0.500
Timepoint3        -0.573  0.500  0.500
Timepoint4        -0.565  0.493  0.493  0.493
Group1:Timepoint2  0.402 -0.702 -0.702 -0.351 -0.346
Group1:Timepoint3  0.402 -0.702 -0.351 -0.702 -0.346  0.493
Group1:Timepoint4  0.396 -0.691 -0.346 -0.346 -0.702  0.485  0.487

Standardized Within-Group Residuals:
        Min          Q1         Med          Q3         Max
-2.38837839 -0.60338871 -0.07380036  0.55180066  2.91650759

Number of Observations: 155
Number of Groups: 20

> res_HR <- residuals(model_HR)
> shapiro.test(res_HR)

        Shapiro-Wilk normality test

data:  res_HR
W = 0.99189, p-value = 0.5266
```

```
> hist(res_HR, main = "HR residuals", xlab = "Residuals")
> qqnorm(res_HR, main = "HR residuals")
> qqline(res_HR)

#Confidence intervals
> # HR
> coef_HR <- summary(model_HR)$tTable
> coef_HR <- cbind(
+   coef_HR,
+   Lower_CI = coef_HR[, "Value"] - 1.96 * coef_HR[, "Std.Error"],
+   Upper_CI = coef_HR[, "Value"] + 1.96 * coef_HR[, "Std.Error"]
+ )
> coef_HR
                        Value Std.Error  DF     t-value      p-value    Low
er_CI
(Intercept)       116.1333333  3.710698 128 31.29689545 7.893488e-62 108.8
60365
Group1             -0.3583333  4.250979 128 -0.08429432 9.329541e-01  -8.6
90251
Timepoint2         -4.0750000  4.250979 128 -0.95860282 3.395660e-01 -12.4
06918
Timepoint3         11.1750000  4.250979 128  2.62880650 9.617511e-03   2.8
43082
Timepoint4         32.1779081  4.312752 128  7.46110857 1.155048e-11  23.7
24915
Group1:Timepoint2   5.9526858  6.055629 128  0.98300037 3.274617e-01  -5.9
16347
Group1:Timepoint3   5.1968339  6.056184 128  0.85810374 3.924391e-01  -6.6
73286
Group1:Timepoint4  -5.9774823  6.148830 128 -0.97213330 3.328177e-01 -18.0
29189
                    Upper_CI
(Intercept)       123.406302
Group1              7.973585
Timepoint2          4.256918
Timepoint3         19.506918
Timepoint4         40.630901
Group1:Timepoint2  17.821719
Group1:Timepoint3  17.066954
Group1:Timepoint4   6.074224
```

#SAP

```
> # SAP
> model_SAP <- lme(
+   SAP ~ Group * Timepoint,
+   random = ~1 | ID1,
+   data = Glycocheck_volume_overload_cats_dataset,
+   na.action = na.omit
+ )
>
> summary(model_SAP)
Linear mixed-effects model fit by REML
  Data: Glycocheck_volume_overload_cats_dataset
       AIC      BIC    logLik
  1213.204 1242.972 -596.6021
```

```
Random effects:
 Formula: ~1 | ID1
        (Intercept) Residual
StdDev:    3.853892 13.21671

Fixed effects:  SAP ~ Group * Timepoint
                      Value Std.Error  DF  t-value p-value
(Intercept)       121.46667  3.078423 126 39.45743  0.0000
Group1            -11.02050  4.369735 126 -2.52201  0.0129
Timepoint2         -9.14583  4.179489 126 -2.18827  0.0305
Timepoint3         -6.64167  4.179489 126 -1.58911  0.1145
Timepoint4          6.65683  4.237021 126  1.57111  0.1187
Group1:Timepoint2  11.72050  6.046711 126  1.93833  0.0548
Group1:Timepoint3  12.56003  6.082348 126  2.06500  0.0410
Group1:Timepoint4   8.47314  6.166079 126  1.37415  0.1718
 Correlation: 
                  (Intr) Group1 Tmpnt2 Tmpnt3 Tmpnt4 Gr1:T2 Gr1:T3
Group1            -0.649                                          
Timepoint2        -0.679  0.478                                   
Timepoint3        -0.679  0.478  0.500                            
Timepoint4        -0.670  0.471  0.493  0.493                     
Group1:Timepoint2  0.469 -0.723 -0.691 -0.346 -0.341              
Group1:Timepoint3  0.466 -0.717 -0.344 -0.687 -0.339  0.518       
Group1:Timepoint4  0.460 -0.708 -0.339 -0.339 -0.687  0.511  0.508

Standardized Within-Group Residuals:
       Min         Q1        Med         Q3        Max 
-2.0480089 -0.5767180 -0.1486577  0.4852999  4.2018588 

Number of Observations: 153
Number of Groups: 20 

> res_SAP <- residuals(model_SAP)
> shapiro.test(res_SAP)

	Shapiro-Wilk normality test

data:  res_SAP
W = 0.95684, p-value = 0.0001075

> hist(res_SAP, main = "SAP residuals", xlab = "Residuals")
> qqnorm(res_SAP, main = "SAP residuals")
> qqline(res_SAP)

> # SAP
> coef_SAP <- summary(model_SAP)$tTable
> coef_SAP <- cbind(
+   coef_SAP,
+   Lower_CI = coef_SAP[, "Value"] - 1.96 * coef_SAP[, "Std.Error"],
+   Upper_CI = coef_SAP[, "Value"] + 1.96 * coef_SAP[, "Std.Error"]
+ )
> coef_SAP
                       Value Std.Error  DF    t-value      p-value    Lower
_CI
(Intercept)       121.466667  3.078423 126 39.457427 8.899683e-73 115.4329
568
Group1            -11.020499  4.369735 126 -2.522006 1.291506e-02 -19.5851
793
```

```
Timepoint2          -9.145833  4.179489 126 -2.188266 3.049440e-02 -17.3376
327
Timepoint3          -6.641667  4.179489 126 -1.589110 1.145418e-01 -14.8334
661
Timepoint4           6.656832  4.237021 126  1.571111 1.186646e-01  -1.6477
291
Group1:Timepoint2   11.720499  6.046711 126  1.938326 5.481987e-02  -0.1310
550
Group1:Timepoint3   12.560029  6.082348 126  2.064997 4.097422e-02   0.6386
274
Group1:Timepoint4    8.473139  6.166079 126  1.374154 1.718335e-01  -3.6123
753
                     Upper_CI
(Intercept)       127.5003765
Group1             -2.4558184
Timepoint2         -0.9540339
Timepoint3          1.5501327
Timepoint4         14.9613938
Group1:Timepoint2  23.5720527
Group1:Timepoint3  24.4814301
Group1:Timepoint4  20.5586530

> # MAP
> model_MAP <- lme(
+   MAP ~ Group * Timepoint,
+   random = ~1 | ID1,
+   data = Glycocheck_volume_overload_cats_dataset,
+   na.action = na.omit
+ )
>
> summary(model_MAP)
Linear mixed-effects model fit by REML
  Data: Glycocheck_volume_overload_cats_dataset
       AIC      BIC    logLik
  1144.299 1174.066 -562.1493

Random effects:
 Formula: ~1 | ID1
        (Intercept) Residual
StdDev:    4.688102 10.10528

Fixed effects:  MAP ~ Group * Timepoint
                      Value Std.Error  DF  t-value p-value
(Intercept)        88.73333  2.490934 126 35.62252  0.0000
Group1             -5.81078  3.345887 126 -1.73669  0.0849
Timepoint2        -10.33333  3.195570 126 -3.23364  0.0016
Timepoint3         -7.05000  3.195570 126 -2.20618  0.0292
Timepoint4          2.38280  3.240913 126  0.73522  0.4636
Group1:Timepoint2   4.54828  4.626730 126  0.98304  0.3275
Group1:Timepoint3   6.11952  4.652683 126  1.31527  0.1908
Group1:Timepoint4   2.73293  4.718770 126  0.57916  0.5635
 Correlation:
                  (Intr) Group1 Tmpnt2 Tmpnt3 Tmpnt4 Gr1:T2 Gr1:T3
Group1            -0.613
Timepoint2        -0.641  0.478
Timepoint3        -0.641  0.478  0.500
Timepoint4        -0.632  0.470  0.493  0.493
Group1:Timepoint2  0.443 -0.723 -0.691 -0.345 -0.340
```

```
Group1:Timepoint3  0.441 -0.717 -0.343 -0.687 -0.338  0.519
Group1:Timepoint4  0.434 -0.707 -0.339 -0.339 -0.687  0.511  0.509

Standardized Within-Group Residuals:
       Min         Q1        Med         Q3        Max
-1.9786487 -0.6755583 -0.1174402  0.5102296  2.8821243

Number of Observations: 153
Number of Groups: 20

> res_MAP <- residuals(model_MAP)
> shapiro.test(res_MAP)

	Shapiro-Wilk normality test

data:  res_MAP
W = 0.97799, p-value = 0.01491

> hist(res_MAP, main = "MAP residuals", xlab = "Residuals")
> qqnorm(res_MAP, main = "MAP residuals")
> qqline(res_MAP)

> # MAP
> coef_MAP <- summary(model_MAP)$tTable
> coef_MAP <- cbind(
+   coef_MAP,
+   Lower_CI = coef_MAP[, "Value"] - 1.96 * coef_MAP[, "Std.Error"],
+   Upper_CI = coef_MAP[, "Value"] + 1.96 * coef_MAP[, "Std.Error"]
+ )
> coef_MAP
                       Value Std.Error  DF    t-value      p-value    Lower
_CI
(Intercept)        88.733333  2.490934 126 35.6225207 1.221770e-67   83.851
104
Group1             -5.810778  3.345887 126 -1.7366929 8.488539e-02 -12.368
716
Timepoint2        -10.333333  3.195570 126 -3.2336429 1.560604e-03 -16.596
651
Timepoint3         -7.050000  3.195570 126 -2.2061789 2.918415e-02 -13.313
318
Timepoint4          2.382798  3.240913 126  0.7352242 4.635692e-01  -3.969
391
Group1:Timepoint2   4.548278  4.626730 126  0.9830438 3.274698e-01  -4.520
112
Group1:Timepoint3   6.119520  4.652683 126  1.3152670 1.908087e-01  -2.999
738
Group1:Timepoint4   2.732932  4.718770 126  0.5791619 5.635139e-01  -6.515
858
                    Upper_CI
(Intercept)       93.6155632
Group1             0.7471603
Timepoint2        -4.0700154
Timepoint3        -0.7866821
Timepoint4         8.7349862
Group1:Timepoint2 13.6166682
Group1:Timepoint3 15.2387781
Group1:Timepoint4 11.9817213
>
```

```
> # DAP
> model_DAP <- lme(
+   DAP ~ Group * Timepoint,
+   random = ~1 | ID1,
+   data = Glycocheck_volume_overload_cats_dataset,
+   na.action = na.omit
+ )
>
> summary(model_DAP)
Linear mixed-effects model fit by REML
  Data: Glycocheck_volume_overload_cats_dataset
       AIC      BIC    logLik
  1179.926 1209.694 -579.9632

Random effects:
 Formula: ~1 | ID1
        (Intercept) Residual
StdDev:    5.948637 11.30449

Fixed effects:  DAP ~ Group * Timepoint
                     Value Std.Error  DF   t-value p-value
(Intercept)       71.58333  2.856377 126 25.060883  0.0000
Group1            -4.41787  3.744397 126 -1.179861  0.2403
Timepoint2        -7.90417  3.574794 126 -2.211083  0.0288
Timepoint3        -6.20000  3.574794 126 -1.734366  0.0853
Timepoint4         1.02973  3.625907 126  0.283993  0.7769
Group1:Timepoint2 -1.43213  5.176839 126 -0.276642  0.7825
Group1:Timepoint3  3.91255  5.205463 126  0.751624  0.4537
Group1:Timepoint4  1.10850  5.279989 126  0.209943  0.8341
 Correlation:
                  (Intr) Group1 Tmpnt2 Tmpnt3 Tmpnt4 Gr1:T2 Gr1:T3
Group1            -0.597
Timepoint2        -0.626  0.477
Timepoint3        -0.626  0.477  0.500
Timepoint4        -0.617  0.470  0.493  0.493
Group1:Timepoint2  0.432 -0.723 -0.691 -0.345 -0.340
Group1:Timepoint3  0.430 -0.717 -0.343 -0.687 -0.338  0.519
Group1:Timepoint4  0.424 -0.707 -0.339 -0.339 -0.687  0.511  0.509

Standardized Within-Group Residuals:
       Min         Q1        Med         Q3        Max
-1.7401143 -0.7155888 -0.1580445  0.6754516  2.8134677

Number of Observations: 153
Number of Groups: 20

> res_DAP <- residuals(model_DAP)
> shapiro.test(res_DAP)

        Shapiro-Wilk normality test

data:  res_DAP
W = 0.97132, p-value = 0.002775

> hist(res_DAP, main = "DAP residuals", xlab = "Residuals")
> qqnorm(res_DAP, main = "DAP residuals")
```

```
> qqline(res_DAP)

> # DAP
> coef_DAP <- summary(model_DAP)$tTable
> coef_DAP <- cbind(
+   coef_DAP,
+   Lower_CI = coef_DAP[, "Value"] - 1.96 * coef_DAP[, "Std.Error"],
+   Upper_CI = coef_DAP[, "Value"] + 1.96 * coef_DAP[, "Std.Error"]
+ )
> coef_DAP
                      Value Std.Error  DF    t-value      p-value   Lower_
CI
(Intercept)       71.583333  2.856377 126 25.0608830 8.652290e-51  65.9848
34
Group1            -4.417869  3.744397 126 -1.1798610 2.402783e-01 -11.7568
88
Timepoint2        -7.904167  3.574794 126 -2.2110831 2.883410e-02 -14.9107
63
Timepoint3        -6.200000  3.574794 126 -1.7343656 8.529885e-02 -13.2065
96
Timepoint4         1.029733  3.625907 126  0.2839933 7.768817e-01  -6.0770
45
Group1:Timepoint2 -1.432131  5.176839 126 -0.2766421 7.825083e-01 -11.5787
37
Group1:Timepoint3  3.912551  5.205463 126  0.7516240 4.536788e-01  -6.2901
56
Group1:Timepoint4  1.108499  5.279989 126  0.2099434 8.340510e-01  -9.2402
80
                    Upper_CI
(Intercept)       77.1818325
Group1             2.9211505
Timepoint2        -0.8975703
Timepoint3         0.8065964
Timepoint4         8.1365114
Group1:Timepoint2  8.7144736
Group1:Timepoint3 14.1152574
Group1:Timepoint4 11.4572771
>


> # Temp
> model_Temp <- lme(
+   Temp ~ Group * Timepoint,
+   random = ~1 | ID1,
+   data = Glycocheck_volume_overload_cats_dataset,
+   na.action = na.omit
+ )
>
> summary(model_Temp)
Linear mixed-effects model fit by REML
  Data: Glycocheck_volume_overload_cats_dataset
       AIC      BIC    logLik
  338.2117 367.8402 -159.1059

Random effects:
 Formula: ~1 | ID1
        (Intercept)  Residual
StdDev:   0.3315791 0.6292234
```

```
Fixed effects:  Temp ~ Group * Timepoint
                      Value Std.Error  DF   t-value p-value
(Intercept)        36.80000 0.1590388 124 231.39008  0.0000
Group1              0.24198 0.2018487 124   1.19883  0.2329
Timepoint2         -0.29500 0.1989779 124  -1.48258  0.1407
Timepoint3          0.42544 0.2018233 124   2.10797  0.0370
Timepoint4          0.70149 0.2018233 124   3.47575  0.0007
Group1:Timepoint2 -0.18921 0.2850758 124  -0.66372  0.5081
Group1:Timepoint3  0.03730 0.2921973 124   0.12765  0.8986
Group1:Timepoint4 -0.11787 0.2897123 124  -0.40684  0.6848
 Correlation: 
                  (Intr) Group1 Tmpnt2 Tmpnt3 Tmpnt4 Gr1:T2 Gr1:T3
Group1            -0.617                                          
Timepoint2        -0.626  0.493                                   
Timepoint3        -0.617  0.486  0.493                            
Timepoint4        -0.617  0.486  0.493  0.486                     
Group1:Timepoint2  0.437 -0.706 -0.698 -0.344 -0.344              
Group1:Timepoint3  0.426 -0.691 -0.340 -0.691 -0.336  0.488       
Group1:Timepoint4  0.430 -0.697 -0.343 -0.339 -0.697  0.492  0.483

Standardized Within-Group Residuals:
        Min          Q1         Med          Q3         Max 
-2.67954612 -0.53445054  0.08114471  0.60090189  2.85579397 

Number of Observations: 151
Number of Groups: 20 

> res_Temp <- residuals(model_Temp)
> shapiro.test(res_Temp)

	Shapiro-Wilk normality test

data:  res_Temp
W = 0.98668, p-value = 0.1571

> hist(res_Temp, main = "Temperature residuals", xlab = "Residuals")
> qqnorm(res_Temp, main = "Temperature residuals")
> qqline(res_Temp)

> # Temperature
> coef_Temp <- summary(model_Temp)$tTable
> coef_Temp <- cbind(
+   coef_Temp,
+   Lower_CI = coef_Temp[, "Value"] - 1.96 * coef_Temp[, "Std.Error"],
+   Upper_CI = coef_Temp[, "Value"] + 1.96 * coef_Temp[, "Std.Error"]
+ )
> coef_Temp
                        Value Std.Error  DF     t-value       p-value    Lo
wer_CI
(Intercept)        36.8000000 0.1590388 124 231.3900758 2.546584e-165 36.48
828395
Group1              0.2419827 0.2018487 124   1.1988323  2.328792e-01 -0.15
364070
Timepoint2         -0.2950000 0.1989779 124  -1.4825766  1.407240e-01 -0.68
499672
Timepoint3          0.4254370 0.2018233 124   2.1079679  3.704704e-02  0.02
986338
```

```
Timepoint4         0.7014874 0.2018233 124   3.4757507  7.027536e-04  0.30
591378
Group1:Timepoint2 -0.1892105 0.2850758 124  -0.6637200  5.081017e-01 -0.74
795914
Group1:Timepoint3  0.0372976 0.2921973 124   0.1276453  8.986365e-01 -0.53
540907
Group1:Timepoint4 -0.1178669 0.2897123 124  -0.4068411  6.848261e-01 -0.68
570295
                     Upper_CI
(Intercept)       37.11171605
Group1             0.63760613
Timepoint2         0.09499672
Timepoint3         0.82101065
Timepoint4         1.09706106
Group1:Timepoint2  0.36953809
Group1:Timepoint3  0.61000427
Group1:Timepoint4  0.44996920
```
