# Supplementary material for: Effects of transient intravascular volume overload on endothelial glycocalyx thickness in healthy anesthetized cats
Source: Front Vet Sci. 2026 Oct 14;13:1742967. doi: 10.3389/fvets.2026.1742967 (PMC13713276; doi:10.3389/fvets.2026.1742967)

Supplementary material 4. Individual changes in perfused boundary regions over time in vessels with diameters of 5 – 9 µm (A), 10 – 19 µm (B) and 20 – 25 µm (C) in 20 healthy anaesthetized cats with transient intravascular volume overload or control

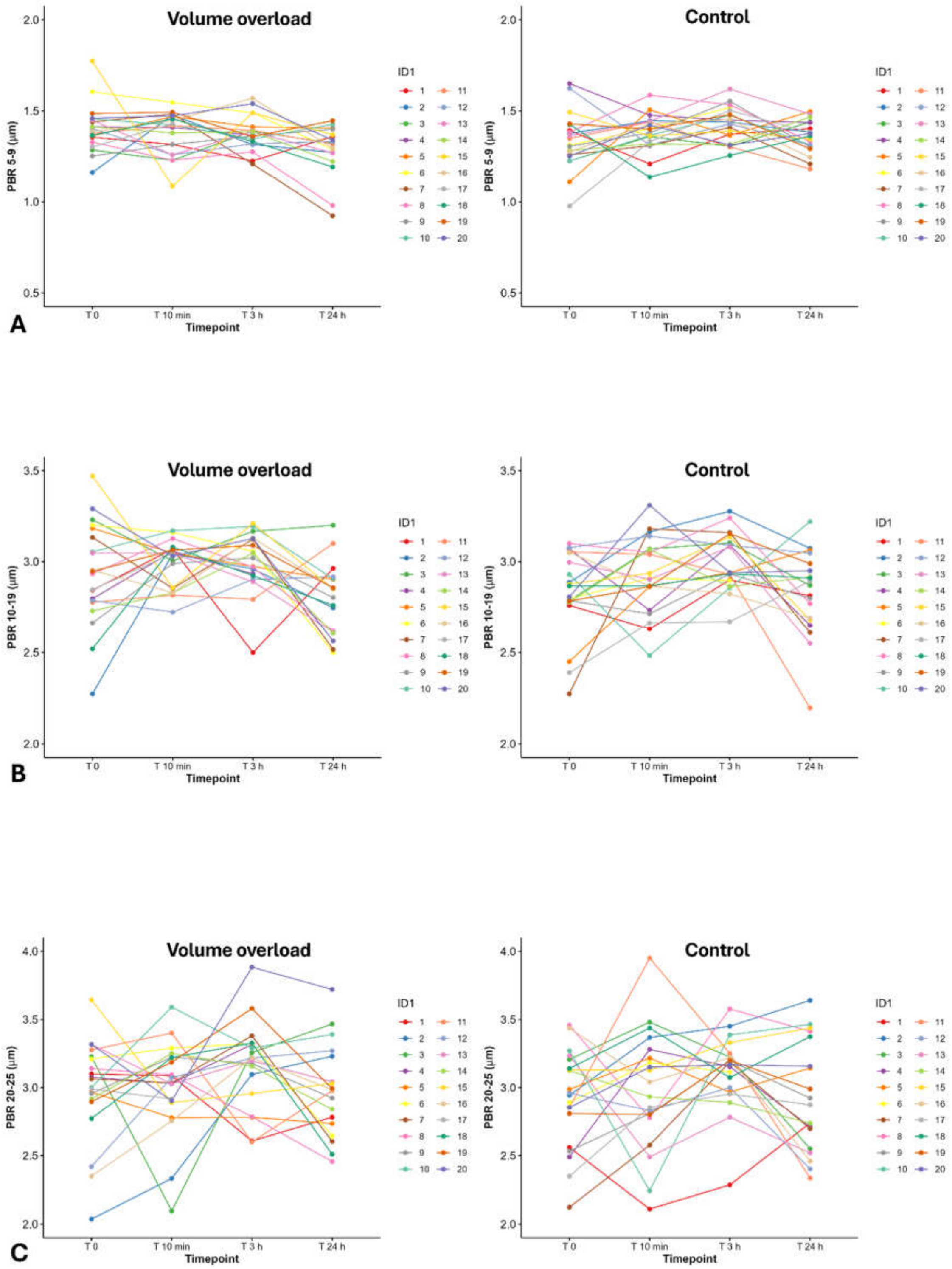

Supplement: Supplementary file 3 [file Data_Sheet_3.PDF]
