# Supplementary material for: Effects of transient intravascular volume overload on endothelial glycocalyx thickness in healthy anesthetized cats
Source: Front Vet Sci. 2026 Oct 14;13:1742967. doi: 10.3389/fvets.2026.1742967 (PMC13713276; doi:10.3389/fvets.2026.1742967)
Supplement: Supplementary file 4 [file Data_Sheet_4.PDF]

Supplementary Table 1. Summary of linear mixed-effects models used in the statistical analysis.

Analysis	Dependent variable(s)	Independent variable(s)	Random effect
Model 1 – GlycoCheck™ vs group x timepoint	GlycoCheck™ parameters	Group × Timepoint	Cat
Model 1.1	Valid vessel density	〃	〃
Model 1.2	Percentage RBC filling	〃	〃
Model 1.3	Median P50	〃	〃
Model 1.4	PBR 5 - 25	〃	〃
Model 1.5	PBR 5 – 9	〃	〃
Model 1.6	PBR 10 – 19	〃	〃
Model 1.7	PBR 20 – 25	〃	〃
Model 2 - GlycoCheck™ vs PCV	GlycoCheck™ parameters	PCV	Cat
Model 2.1	Valid vessel density	〃	〃
Model 2.2	Percentage RBC filling	〃	〃
Model 2.3	Median P50	〃	〃
Model 2.4	PBR 5 - 25	〃	〃
Model 2.5	PBR 5 – 9	〃	〃
Model 2.6	PBR 10 – 19	〃	〃
Model 2.7	PBR 20 – 25	〃	〃
Model 3 – PCV vs group x timepoint	PCV	Group × Timepoint	Cat
Model 4 – TS vs group x timepoint	TS	Group × Timepoint	Cat
Model 5 – PBR vs PCV sensitivity analysis	PBR	Group × Timepoint + PCV	Cat
Model 5.1	PBR 5 - 25	〃	〃
Model 5.2	PBR 5 – 9	〃	〃
Model 5.3	PBR 10 – 19	〃	〃
Model 5.4	PBR 20 – 25	〃	〃
Model 6 – PBR vs anesthetic variable HR	PBR	HR	Cat
Model 6.1	PBR 5 - 25	〃	〃
Model 6.2	PBR 5 – 9	〃	〃
Model 6.3	PBR 10 – 19	〃	〃
Model 6.4	PBR 20 – 25	〃	〃
Model 7 - PBR vs anesthetic variable MAP	PBR	MAP	Cat
Model 7.1	PBR 5 - 25	〃	〃
Model 7.2	PBR 5 – 9	〃	〃
Model 7.3	PBR 10 – 19	〃	〃
Model 7.4	PBR 20 – 25	〃	〃

Analysis	Dependent variable(s)	Independent variable(s)	Random effect
Model 8 - PBR vs anesthetic variable Temperature	PBR	Temperature	Cat
Model 8.1	PBR 5 - 25	〃	〃
Model 8.2	PBR 5 – 9	〃	〃
Model 8.3	PBR 10 – 19	〃	〃
Model 8.4	PBR 20 – 25	〃	〃
Model 9 – anesthetic variables vs group x timepoint	Anaesthetic variables	Group × Timepoint	Cat
Model 9.1	HR	〃	〃
Model 9.2	SAP	〃	〃
Model 9.3	DAP	〃	〃
Model 9.4	MAP	〃	〃
Model 9.5	Temperature	〃	〃
